# Insight and equality: A systematic review and meta-analysis of socio-demographic associations

**DOI:** 10.1177/00207640211036174

**Published:** 2021-08-04

**Authors:** Kevin Ariyo, Alex Ruck Keene, Anthony S David, Gareth S Owen

**Affiliations:** 1 Department of Psychological Medicine, Institute of Psychiatry, Psychology and Neuroscience, King’s College London, UK; 2 Dickson Poon School of Law, King’s College London, UK; 3 Division of Psychiatry, UCL Institute of Mental Health, University College London, UK

**Keywords:** Mental health, mental health law, systematic review, meta analysis, cognitive psychology, neuropsychiatry

## Abstract

**Background:**

Insight into illness is often used in clinical and legal contexts, for example, as evidence of decision-making capacity. However, it is unclear whether this disadvantages certain groups protected under equality legislation. To our knowledge, this question has yet to be addressed systematically. Therefore, the present study reviews empirical studies that look at the relationship between insight and sociodemographic variables.

**Methods:**

A systematic search of six bibliographic databases (CENTRAL, CINAHL, Cochrane Library of Systematic Reviews, EMBASE, MEDLINE and PsycINFO) was conducted, which yielded 6,192 results. Study characteristics and outcomes (associations between insight and socio-demographic variables) were then extracted from 207 eligible studies. This included protected characteristics under the Equality Act (2010): age, sex, ethnicity, marital status and religion. Weighted confidence estimates were calculated and relevant moderators included in a random effects meta-analysis. A study protocol was registered prospectively on PROSPERO, ID: CRD42019120117.

**Results:**

Insight was not strongly associated with any sociodemographic variable. Better insight was weakly but significantly associated with white ethnicity, being employed, younger age and more years of education. The age associations were mostly explained by relevant moderating variables. For people with schizophrenia, the associations between sociodemographic variables and insight were comparable to associations with decision making capacity.

**Conclusions:**

Our results suggest that insight is not strongly associated with any sociodemographic variables. Further research is needed to clarify potential associations, particularly with non-white ethnicity and proxies for social support.

## Introduction

Although assessments of mental capacity (or competence) and assessments of compulsory treatment for mental disorder are a core part of a healthcare professional’s duty (Appelbaum & Grisso, 1988; Johnston & Liddle, 2007), they are inherently legal assessments (Allen, 2014). In contrast, the concept of insight is inherently clinical: it is based on a judgement made by a clinician about the patient as part of making a diagnosis and planning treatment (David, 2020).

Clinical insight may be defined as the degree of awareness a person has that they may be suffering from a biomedical condition and its effects, and the person’s willingness or ability to reflect on this (David, 1990, 2020). This concept is often influential in clinical assessment of mental capacity (Allen, 2014). For example, a recent review has found that one third of reported health and welfare cases pertaining to capacity decided by the Court of Protection in England and Wales referenced a person’s ‘lack of insight’ (Case, 2016). It is thought that insight is relevant to terms commonly used in the functional test of capacity, such as ‘appreciation’ and ‘using or weighing’ (Ruck Keene et al., 2019). There is particularly strong empirical evidence in psychosis for an association between insight and capacity to consent to treatment decisions (Owen et al., 2009; Spencer et al., 2017). ‘Lack of insight’ is also frequently used as partial justification for compulsory detention under the traditional mental health laws (Cairns et al., 2005) and is often associated with a greater likelihood of being admitted (Kelly et al., 2004; Walker et al., 2019). A better understanding of sociodemographic associations may therefore be relevant to concerns in Europe and the USA, about higher detention rates in people of African or Caribbean descent (Barnett et al., 2019; Snowden et al., 2009), and in men of all ethnic backgrounds (NHS Digital, 2020).

Owing to its multiply-determined and dynamic nature, there are several conceptual models of insight that have resulted in different measures and these must be taken into account. Five main procedures exist for operationalising or quantifying insight (Marková, 2005) (i) a clinician’s statement on a patient ‘having’ or ‘lacking’ insight in a clinical report; (ii) a clinician’s rating on a validated scale, based on a routine, structured or semi structured interview; (iii) a person’s rating on self-report instruments; (iv) the discrepancy between the individual’s own and caregiver ratings on a list of behaviours and abilities; and (v) the difference between subjective ratings and objective scores on a neuropsychological test.

Critics have suggested that insight may discriminate, because it may serve as a proxy for existing value judgements that are susceptible to biases against those with protected characteristics like age, gender, ethnicity and religion or belief (Department for Health and Social Care, 2018; Mishra et al., 2009). This is a concern, not least because judges in the courts must interpret clinical evidence from a lay perspective. Such discrimination is likely to be indirect – in other words – the application of a legally neutral concept in such a way as to produce an unjustified and adverse difference in treatment on the basis of a particular status. For example, a GP practice that requires proof of address may be found to indirectly discriminate against Gypsies and Travellers, if such rules render it more difficult for them to access their service. Likewise, if the application of the concept of insight in practice leads to indirect discrimination, then reliance upon it to justify either a finding of mental incapacity or detention under mental health legislation would be questionable.

Before we question whether insight is an indirectly discriminatory construct we must first identify where disparities exist. That is, are certain groups more likely to be judged as having poor insight? Previous attempts to address the relationship between insight and sociodemographic variables have been unsystematic and partial. A comprehensive but non-systematic narrative literature review of clinical studies 15 years ago found mixed results, with no conclusive evidence of major demographic influences on insight (Marková, 2005). Although many of the studies cited found no significant relationship between insight and sociodemographic factors, some significant relationships were reported, and the overall strength of each effect remains unquantified.

If sociodemographic factors predict insight, then this may raise concerns around discrimination. To properly address whether the application of insight is discriminatory, further contextual information would be needed. For example, increasing age is strongly associated with the incidence and severity of Alzheimer’s disease symptoms (Dukart et al., 2013; Wilson et al., 2000), but not schizophrenia symptoms (Díaz-Caneja et al., 2015). As such, illness severity may be a more plausible explanation for age disparities in insight for dementia, but not for schizophrenia. Most clinical studies do not explicitly test hypotheses based on sociodemographic factors and insight, so these contextual factors have been neglected. Indeed, it is common practice for such studies to partition out the effects of variables such as age, gender and socioeconomic status, rather than to consider them as predictors (Schandrin et al., 2019; Shaked et al., 2019). It is therefore relevant to consider not just the magnitude of any observed disparities, but also whether intermediary variables may help to explain why these exist.

Similarly, there remains a question as to whether using insight as legal evidence would lead to more discriminatory capacity assessments. One systematic review explored sociodemographic associations with capacity to consent to treatment (age, gender, ethnicity, socioeconomic status and education), which only found some evidence for more years of education predicting better capacity (Spencer et al., 2017). However, to our knowledge, no study has compared sociodemographic associations with insight and capacity within the same sample.

We sought to determine whether insight is associated with such sociodemographic variables by means of a systematic review and meta-analysis of the scientific literature. If insight is not applied in a discriminatory fashion, we would not expect to find large disparities between socio-demographic groups, or between physically disabled and non-disabled groups. Furthermore, any observed disparities would be capable of an explanation and/or justification based upon a factor relevant to that group. We hypothesised that:

(a) No sociodemographic factor will strongly predict insight across all clinical populations.
(b) If a sociodemographic factor predicts insight, a conceptually relevant variable to that factor will explain some of the variance in this relationship.
(c) No sociodemographic factor will predict insight to a significantly stronger extent than it predicts mental capacity.

## Methods

### Search strategy

For the present review, we developed a tailored search strategy based on relevant keywords, headings and subject headings, using six online bibliographic databases: CENTRAL, CINAHL, Cochrane Library of Systematic Reviews, EMBASE, MEDLINE, PsycINFO. Example search terms for insight included ‘clinical insight’, ‘anosognosia’, ‘awareness of deficit’ and several common insight measures. We conducted the initial search on 1st March 2019 and updated this on 2nd October 2020. Time restrictions were not set.

### Selection criteria

One reviewer (K.A) led the study selection and another reviewer (G.O) conducted reliability checks for 10% of full text articles. This was done according to a prospectively published protocol (see Supplemental Appendix). We achieved a kappa statistic of 0.8, indicating very substantial inter-rater agreement (Landis & Koch, 1977).

In brief, peer-reviewed journal articles were selected if they reported (a) in English (b) using a cross-sectional or more robust design (c) a categorical or continuous measure of clinical insight (d) the results of a statistical test comparing insight and at least one socio-demographic or disability variable of interest (age, sex, ethnicity, religion, marital status, education, employment or socioeconomic status, physical health disability) and (e) the relevant analysis included at least 20 participants (or 10 per group). We retrospectively excluded samples with neurodevelopmental and rare genetic disorders, or with children samples, due to minimal results from these populations.

**Figure 1.**
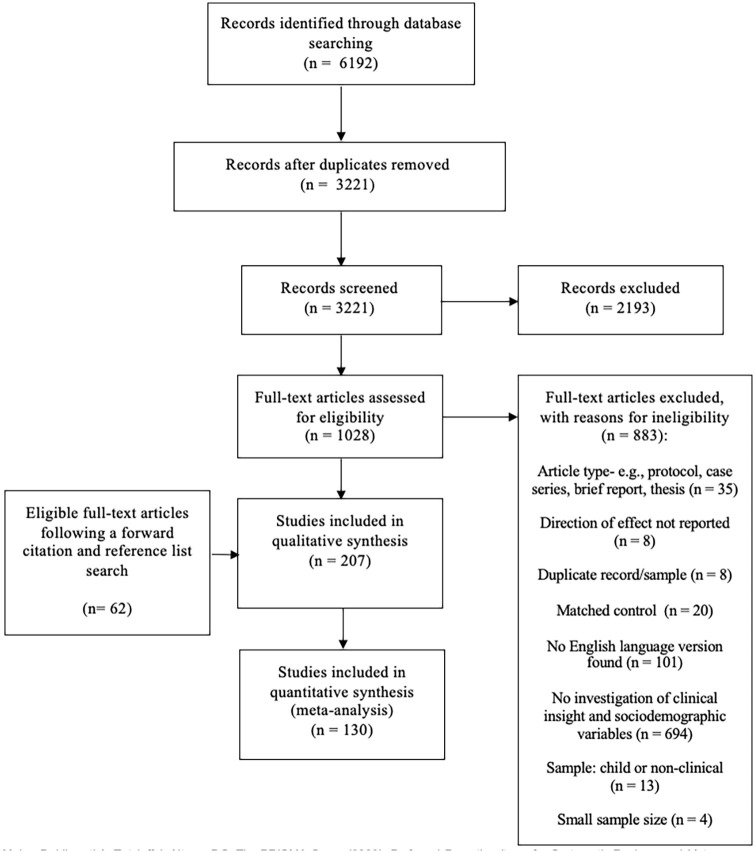
A PRISMA flow diagram outlining the study selection procedure.

The initial database search produced 6,192 results, of which 1,028 potentially relevant studies were screened at full text level and 145 were deemed eligible for inclusion (see Figure 1). A forward citation and backward reference search of eligible studies was conducted using SCOPUS, which yielded a further 62 eligible studies. All 207 eligible studies were included for qualitative synthesis and 130 studies were also selected for meta-analysis, based on further eligibility criteria (see Supplemental Appendix).

We modified our protocol following its initial registration to remove social influence variables from our criteria, as scoping searches revealed this would yield few results. We also restricted our criteria to include only complete or well validated clinical insight scales, to improve specificity. Selection queries were taken to G.O in the first instance, with A.D the final arbiter if there was a disagreement.

### Data extraction

One reviewer (K.A) extracted relevant data from all eligible studies, recording this on a spreadsheet. G.O independently extracted data from 10% of eligible studies, to evaluate consistency. The primary outcome of interest was the association of sociodemographic data (age, sex, ethnicity, marital status, religion, age at onset, education, employment status and socioeconomic status) with insight (measured either continuously or categorically). This was used to calculate the proportion of studies that found at least one significant association with the protected characteristic, the group rated as having significantly better insight (if any) and the method of insight assessment most often associated with these changes. In the event of duplicate datasets, in the first instance we prioritised the article that reported the most detailed statistical test. Alternatively, we excluded the most recently published article. For ethnicity, we considered either white versus non-white where possible, or alternatively white versus the most populous non-white group. We also extracted potentially relevant moderator variables (see below).

### Data analysis

A random effects meta-analysis was conducted using *R* Statistics. We used the Restricted Maximum Likelihood (REML) method and effect sizes were weighted by inverse sampling variance. We calculated Cohen’s *d* when insight was measured as a continuous variable, using established conversion metrics (see Supplemental Appendix for overview).

We calculated Odds Ratios (ORs) where insight was measured as a binary judgement, although artificially dichotomised sociodemographic variables (age, education, etc.) were excluded. For samples in which two or more different insight measures were reported, we calculated a single mean effect size to adhere to the independence assumption. For eligible studies that did not report sufficient information, only adjusted effect sizes were included in the qualitative synthesis only. Each study required a minimum of 20 participants for inclusion into the meta-synthesis and at least ten participants from each group to provide a minimum degree of statistical power.

Then, as pre-specified in our protocol, we selected relevant variables for moderator and subgroup analyses. We considered, for example, whether associations may vary between people with psychiatric (schizophrenia, depression, bipolar disorder or obsessive-compulsive disorder) or neurological diagnoses (dementia, stroke, brain injury or mild cognitive impairment). We also contrasted different types of insight scales, including self-report scales, clinical judgement scales and dual-rater discrepancy scales.

Finally, we conducted sensitivity analyses to assess risk of bias, including heterogeneity (*I*^2^ statistic), influential cases, publication bias and analytic decisions. Our criteria for heterogeneity and influential cases were based on I^2^ statistic (50%–75% = moderate, >75% = substantial) (Higgins et al., 2003) and Cook’s distance (d < 4/k = outlier) (Cohen et al., 2014). We calculated leave-one-out diagnostics for each influential case and decided whether to retain or exclude these on a case-by-case basis. For funnel plots, we judged potentially for funnel plot asymmetry by observation and using Egger’s regression test (Sterne et al., 2011). Full details for analytic decisions are available in the Supplemental Appendix.

## Results

### Characteristics of included studies

A final 207 studies were eligible for inclusion. Seventy-five studies were included in the meta-analysis where insight was expressed as a continuous variable (see Table 1), and 56 studies were included in meta-analysis when insight was expressed as a categorical variable (see Table 2). Three hundred one effect sizes were derived from these studies (See Supplemental Appendix for an overview). An estimated 16,522 (M = 79.82) participants were included in the meta-analysis, with 1,432 participants in the largest independent sample. In addition, 77 studies were retained for qualitative synthesis only.

**Table 1.** The association of sociodemographic factors with insight when measured as a continuous variable.

Demographic	*k*	Weighted mean *d*	95% CI	*p*	*I*^2^ (%)
Younger age	67	0.21***	[0.15, 0.28]	<.0001	52.22
Better educated	43	0.16**	[0.07, 0.25]	.005	57.59
Employed	7	0.23*	[0.06, 0.39]	.007	14.34
White ethnicity	3	0.25*	[0.04, 0.47]	.018	0
Married/Relationship	12	0.14	[−0.08, 0.37]	.209	84.29
Female	27	0.05	[−0.06, 0.16]	.359	61.60

* *p* < .05 **p < .01 *** *p*< .001.

**Table 2.** The odds associated with better insight when measured as a dichotomous variable.

Demographic	*K*	Odds ratio	95% CI	*p*	*1*^2^ (%)
Younger age	47	0.24***	[0.13, 0.34]	<.0001	4.29
Better educated	30	0.17*	[0.04, 0.30]	.012	0.00
Employed	3	0.38	[−0.48, 1.24]	.386	58.72
Married/Relationship	7	0.20	[−0.46, 0.86]	.556	75.70
White ethnicity	3	0.27	[−0.86, 1.39]	.640	47.46
Female	49	0.15*	[0.02, 0.29]	.026	10.85

* *p* < .05 ***p < .0001.

We adopted a modified version of Cohen’s criteria to interpret effect sizes (no effect = below 0.1, small effect = 0.1–0.3, moderate effect = 0.3–0.5, large effect = 0.5 or above), in line with similar research (Spencer et al., 2017). Just over half of the reported effect sizes suggested either small or zero association between sociodemographic characteristics and insight (See Supplemental Appendix). We were unable to find sufficient samples for many of the sociodemographic variable of interest (religion or belief, sexual orientation, pregnancy and maternity and gender reassignment).

For a full overview of included studies, analytical decisions and sensitivity analyses, see the Supplemental Appendix. Two effect sizes were excluded as outliers from each meta-analysis (Ampalam et al., 2012; De Carolis et al., 2015). Heterogeneity was low-to-moderate for each variable (other than for marital status, which was high) and there was little evidence of publication bias. One study contributed 49.22% of the sample for the marital status analysis (*k* = 12), but there was no evidence that this study was particularly influential (Mohamed et al., 2009).

### Sociodemographic predictors of insight

When expressed as a continuous variable, white ethnicity (*d* = 0.25), being employed (*d* = 0.23), younger age (*d* = 0.21) and more years of education (*d* = 0.16) significantly predicted poorer insight to a weak degree. We found no evidence of marital status (*d* = 0.14) or sex (*d* = 0.05) predicting insight. According to our effect size criteria, these sociodemographic variables seem to contribute to a small proportion of the variance in insight across populations.

When expressed as a dichotomous variable, people were more likely to be judged as having good insight if they were younger (OR = 0.24, *p* < .0001) and had more years of education (OR = 0.17, *p* < .05). This is similar to the analysis for continuous variables (see Table 1). Females were also more likely to be judged as having good insight (OR = 0.15, *p* < .05), although this result may have been affected by influential cases (see below). The effects sizes were all small. No other odds ratio reached statistical significance, possibly due to the low sample sizes.

### Moderation and subgroup analyses

**Figure 2.**
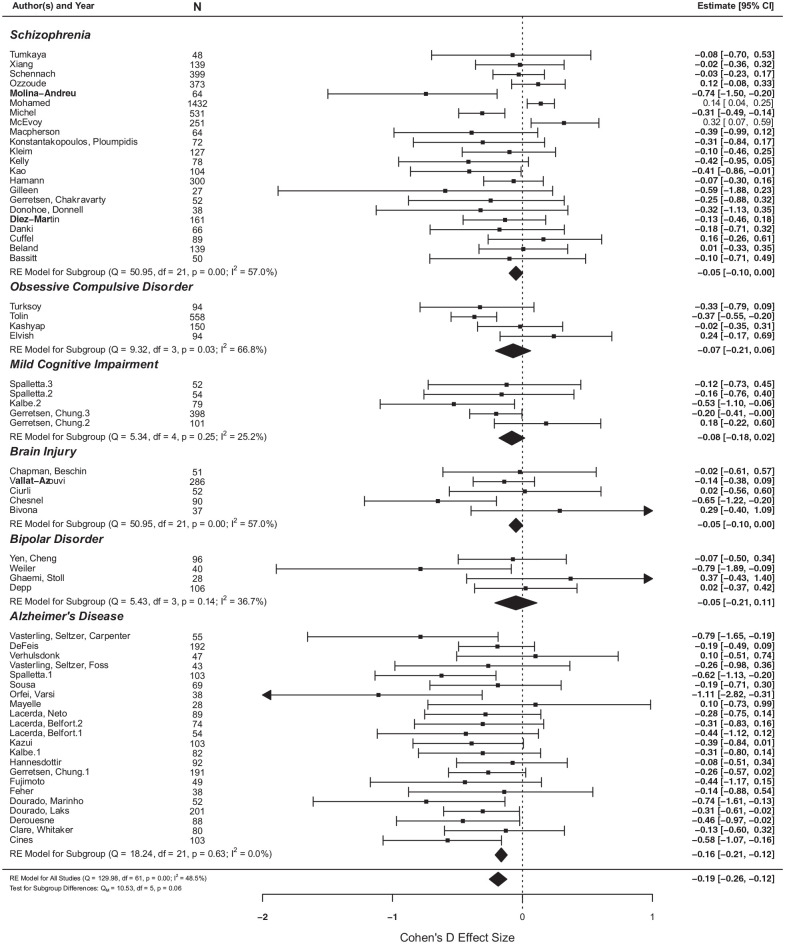
The effect of age on insight, between different diagnostic groups.

We found some evidence that these effects may be context specific, when insight was measured as a continuous variable. The association of age with insight was significantly moderated by the type of diagnosis – that is, whether it was a psychiatric or neurological population (*p* < .001). Subgroup analyses revealed that older age was moderately associated with poor insight in neurological populations (*d* = 0.31, *p* < .0001) (see Figure 2). Conversely, the association between younger age and good insight was far weaker in psychiatric samples (*d* = 0.12, *p* < 0.05).

**Figure 3.**
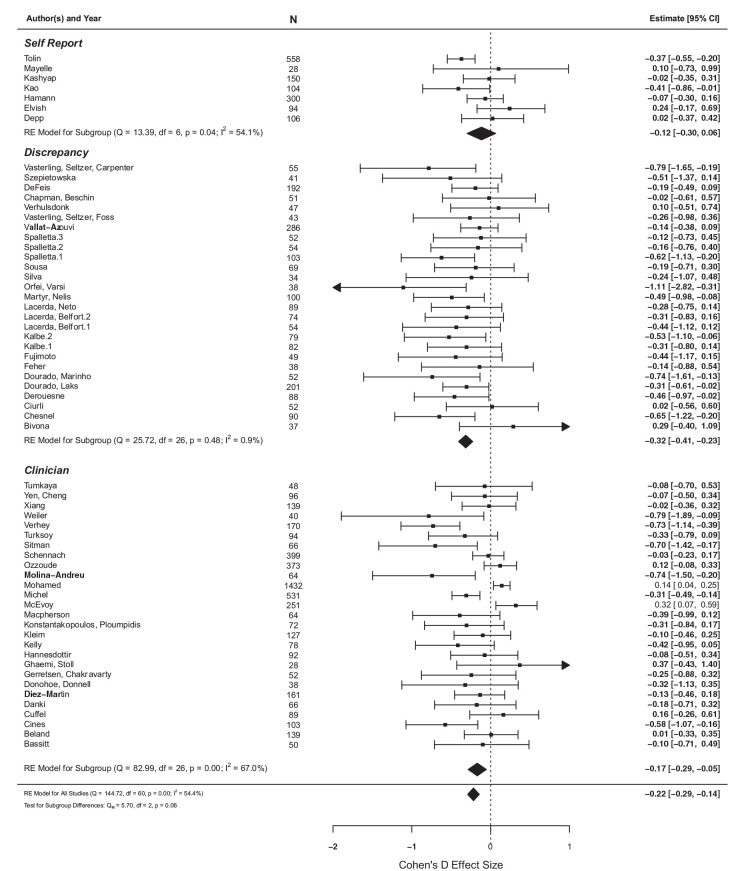
The effect of age on insight between different types of measurement scales.

The association of age with insight was also (marginally) significantly moderated by the type of insight scale used (*p* = .057). Older age significantly associated with poor insight when discrepancy scales (*d* = 0.16, *p* < .05) and clinician-rated scales were used (*d* = 0.16, *p* < 0.05) but not for clinical self-report scales (see Figure 3).

These subgroup analyses are reported in more detail in the Supplemental Appendix.

As shown in Table 3, we also conducted a subgroup analysis of schizophrenia samples, in order to test our third hypothesis comparing insight (as a continuous measure) with mental capacity. We observed a stronger effect of more years of education (*k* = 15, *d* = 0.22, CI [0.05, 0.39]) predicting better insight. However, this effect was not significantly greater than in the non-schizophrenia samples (*k* = 28, *d* = 0.12, CI [0.02, 0.23). We found no evidence for age, marital status or sex predicting insight in Schizophrenia samples (all *p* < .05.) and we did not have sufficient data to include employment or ethnicity in this analysis. These results are broadly similar to associations found with mental capacity.

**Table 3.** Sociodemographic predictors of insight (continuous) for the Schizophrenia sub-sample.

Demographic	*N*	*d*	95% CI	*p*
Younger age	22	0.10	[−0.00, 0.21]	.059
Better educated	15	0.22	[0.05, 0.39]	.012
Married/relationship	8	0.17	[−0.02, 0.37]	.077
Female	18	0.00	[−0.12, 0.13]	.983

### Qualitative synthesis

We undertook a qualitative synthesis of studies included in the meta-analysis (*n* = 130), as well as studies that fit our eligibility criteria, which could not be meta-analysed (*n* = 77). We provide a full overview of these studies in the Supplemental Appendix.

We also explored possible explanations for relationships between insight and the sociodemographic factors included in the meta-analysis. Only a minority of authors proposed an explanation in their manuscript for a significant result. Most of these explanations were psychosocial, for example, relating to access to information, coping mechanisms, behavioural norms and cultural constructions of illness. Some clinical, methodological and neurocognitive explanations were also proposed. See Table 4 for more information.

**Table 4.** Possible explanations (in italics) for the relationship between insight and the main sociodemographic variables, as outlined by study authors.

Predictor of good insight	Proposed explanations			
	Clinical	Psychosocial	Methodological	Neurocognitive
Older age	*Prolonged experience with schizophrenia or bipolar* (Sitman et al., 2012)	*Age-related psychological functioning in traumatic brain injury* (Sherer et al., 2003) *Coping mechanisms for brain injury* (Zimmermann et al., 2017)	*Sampling bias* (Braw et al., 2012)	
Younger age	*Neurodegeneration following stroke* (Kortte et al., 2015) *Severity of dementia and functional impairment* (Spalletta et al., 2012) *Better prognosis due to earlier diagnosis of schizophrenia* (Rathod et al., 2005) *Illness onset of delusional disorder* (Molina-Andreu et al., 2014), *Illness duration of schizophrenia* (Ampalam et al., 2012)	*Younger people with memory impairment over-estimating their deficits* (Kalbe et al., 2005) *Younger bipolar patients having better access to information* (Dias et al., 2008) *Older people with schizophrenia perceived as less self-questioning* (Gilleen et al., 2012)	*Sampling bias* (Kazui et al., 2006)	*Higher premorbid functioning in younger MCI patients* (Kalbe et al., 2005)
Better education		*Education leading to better compensatory mechanisms for, and recognition of, dementia* (Mograbi et al., 2012), *Education leading to better access to information for bipolar disorder* (Dias et al., 2008) *Education leading to better knowledge of dementia* (Aalten et al., 2006)		*Education increasing cognitive reserve in dementia patients* (Castrillo Sanz et al., 2016)
Female gender		*Gender differences in coping mechanisms for schizophrenia* (Pruß et al., 2012), *Gendered cultural norms in help seeking for memory impairment* (Liu et al., 2017)		*Gender differences in verbal memory* (Pruß et al., 2012)
Male gender	*Gender differences during depressive phase of bipolar* (de Assis da Silva et al., 2015),			
White ethnicity		*Cultural differences in conceptualisations of, or help seeking for severe mental illness* (Goldberg et al., 2001; McEvoy et al., 2006; Rathod et al., 2005) *Client-patient interaction confounded by cultural differences* (McEvoy et al., 2006)	*Sampling bias and high dropout rates of African American schizophrenia patients* (Rathod et al., 2005), *Racial bias in evaluation of non-white people with severe mental illness* (McEvoy et al., 2006; Rathod et al., 2005)	

In addition, some sociodemographic variables were only assessed in the qualitative synthesis studies. These included socioeconomic status (*n* = 8), urban status (*n* = 4), migration status (*n* = 3) and body mass index (BMI) (*n* = 2) and religion (*n* = 2).

Two Indian studies of psychosis patients found that people from urban areas had significantly better insight than people in rural areas (both *p* < .005) (Ampalam et al., 2012; Garg et al., 2018). The latter suggested that this was due to variations in cultural beliefs surrounding illness, although this was only supported with secondary evidence. However, a further two studies, of psychiatric inpatients in China (Fu et al., 2017) and OCD patients in India (Ravi Kishore et al., 2004), found no significant differences in insight between urban and rural populations.

Two studies also found that higher socioeconomic status predicted better insight in psychosis samples (Aalten et al., 2006; David et al., 1995). Each of these effects were relatively strong (*p* < .01.). However, the remaining six studies did not find a significant relationship between socioeconomic measures and insight. These included two dementia (Martyr et al., 2012; Mograbi et al., 2012) and four psychosis samples (Fennig et al., 1996; Garg et al., 2018; Heinrichs et al., 1985; Moore et al., 1999).

The remaining sociodemographic variables were less common. Three studies investigated migration status in psychosis samples, with only finding migrants to have better insight (Berg et al., 2018) and two finding no significant relationship (A. David et al., 1995; Klaas et al., 2017). One positive relationship was found between BMI and insight in a bipolar disorder sample (Welten et al., 2016) but not in an eating disorder sample (Arbel et al., 2013). Finally, two studies found no significant association between insight and religion, within in sample of patients with alcohol use disorder (Kim et al., 2007) and social anxiety disorder (Vigne et al., 2014).

## Discussion

The present review and meta-analysis largely corroborate previous findings from non-systematic research (Marková, 2005). That is, more often than not, empirical studies find no association between insight and major socio-demographic variables. In order of strength, we found weak but significant effects for white ethnicity, being employed, younger age and better education predicting better insight scores (all effect sizes < 0.3). When we had sufficient statistical power, we found similar results when insight was measured as a binary clinical judgement as when measured as a dimension although the association with insight and ethnicity and insight and employment went away when binary judgement was used. These findings suggest that none of the protected characteristics relevant to equality legislation that we were able to analyse is strongly associated with insight.

The only moderate association was found within a subgroup of patients and this was explained by conceptually relevant variables. Older age only significantly predicted poorer insight in neurological samples, with a moderately strong effect size (*d* = 0.31) (Chesnel et al., 2018; Dourado et al., 2019; Lacerda et al., 2020; Mayelle et al., 2019; Ozzoude et al., 2019). To our knowledge, this is the first empirical analysis to investigate this. The relationship between age and insight is usually attributed to dementia severity and age-related cognitive decline (McDaniel et al., 1995; Ott & Fogel, 1992). This may explain why the age effect was mostly found in discrepancy scales, which are predominantly used for people with neurological conditions. Neurodegeneration would therefore seem a plausible explanation for these age-related disparities.

Some studies found positive associations of continuous measures of insight with white ethnicity and being employed, in samples of patients with severe mental illnesses. These could not be explained by conceptually relevant variables in the present study. This is partly because the number of samples was relatively low, therefore it was difficult to detect variations in a relatively small and homogenous effect. It is plausible that people with poor insight in the employment studies, who had schizophrenia, bipolar disorder or OCD, may have had greater functional impairment (Lysaker et al., 1998; Poon et al., 2010; Schennach et al., 2012). On average, these conditions have a relatively young onset age (Solmi et al., 2021), which could potentially affect longer-term education and career prospects, especially without measures to compensate for any disruptions.

In the studies that found greater insight in white patients, the only explanation that was supported by primary evidence was for sampling bias, as a result of higher dropout rates in African American schizophrenia patients (Rathod et al., 2005). As a result, we cannot conclude from the current literature whether the association between insight scales and ethnicity is explained. Other possible explanations, such as racial bias in assessment (Goldberg et al., 2001; Rathod et al., 2005) and cultural differences in illness conceptualisation (McEvoy et al., 2006), or help-seeking (Rathod et al., 2005), merit further investigation.

Both our moderator analysis and qualitative synthesis indicate the potential for contextual effects. In order to understand fully whether insight is discriminatory it will be necessary to investigate these associations further. This requires the acquisition of more data on protected characteristics and more qualitative studies to get inside contextual effects. Most of the studies reviewed contained no explanation for any observed disparity. In fact, the most common explanations were primarily psychosocial, relating to social norms (Liu et al., 2017; Pruß et al., 2012), socioeconomic privileges (Dias et al., 2008; Mograbi et al., 2012), cultural bias (Rathod et al., 2005) amongst other factors. These psychosocial explanations should be explored further, and may be considered especially relevant when informant reports are used to provide a discrepancy rating of insight.

Finally, we compared our findings to a systematic review of decision-making capacity (DMC) for people with schizophrenia (Spencer et al., 2017). Similar to the results presented here, that review found almost no evidence of age and sex predicting DMC, and some weak evidence of positive associations with white ethnicity and more years of education. This would suggest that insight is no more discriminatory than capacity on these variables. It should be noted that our study of insight and (Spencer et al., 2017) did not measure use of compulsory treatment for mental disorder where assessments involve the concept of risk rather than just insight or mental capacity.

### Strengths

This is the first meta-analysis and meta-synthesis, to our knowledge, to address the question of whether insight assessments are potentially discriminatory. This directly addresses a recommendation made by the UK government’s recent Independent Review of the Mental Health Act (1983) (Department for Health and Social Care, 2018) but is by no means of UK relevance only. We believe the present review to be the most comprehensive of sociodemographic predictors of insight to date. Meta-analytic methods are frequently applied to neurocognitive associations of insight; however, the present paper is novel in that it extends this approach to social variables. Our mixed methods approach has also enabled us to investigate the issue from different perspectives and the results were mostly consistent. Our moderator analysis was able to shed light on these associations in greater detail, where they may otherwise have been overlooked, while our qualitative analysis also benefits from the additional studies that could not be included in the meta synthesis. Finally, we found no indication that our meta-analysis was biased towards significant effects.

### Limitations

Although we sought to evaluate a range of associations, few studies reported on physical health disabilities, and most of the protected characteristics under the Equality Act (2010) were rarely measured (religion or belief, sexual orientation, pregnancy and maternity and gender reassignment). We also note that the General Comment 1 from the Committee on the Rights of Persons with Disabilities considers that the very concept of DMC is discriminatory (Committee on the Rights of Persons with Disabilities, 2018), which would mean that any reliance on a concept such as insight which would underpin a finding of a lack of DMC would, equally, and by definition, be discriminatory on the basis of disability. Even without taking this radical interpretative step, it is crucial to keep the possibility of discrimination in such an ethically important area under continuing review. Furthermore, this study is limited to measures of insight in research studies rather than use of insight in practice. A recent review in England’s Court of Protection, building on previous work (Case, 2016), found that insight measures have not been mentioned as evidence in any published legal case (Gurbai et al., 2020).

We were often limited to post-hoc examinations, because sociodemographic factors were rarely a focus of individual studies. For this reason, we were relatively inclusive in our sampling criteria. This potentially increased the risk of bias but also enabled us to test for moderators. Despite this, heterogeneity was mostly kept to moderate levels.

## Conclusion

Our study demonstrates that none of the included sociodemographic variables relevant to equality legislation were strongly associated with insight across all populations. These results should give some reassurance that insight does not indirectly discriminate in important ethical and legal assessments like mental capacity, but only if used as intended. We raise some possible concern for people from black and minority ethnic backgrounds or who are either unemployed or have received less education. These subgroups were somewhat more likely to have ratings of poor insight, which could place them at increased risk of indirect discrimination. Future research should explore disparities in groups that have been poorly represented in empirical and qualitative research on insight.

## Supplemental Material

sj-pdf-1-isp-10.1177_00207640211036174 – Supplemental material for Insight and equality: A systematic review and meta-analysis of socio-demographic associations Supplemental material, sj-pdf-1-isp-10.1177_00207640211036174 for Insight and equality: A systematic review and meta-analysis of socio-demographic associations by Kevin Ariyo, Alex Ruck Keene, Anthony S David and Gareth S Owen in International Journal of Social Psychiatry

sj-pdf-2-isp-10.1177_00207640211036174 – Supplemental material for Insight and equality: A systematic review and meta-analysis of socio-demographic associations Supplemental material, sj-pdf-2-isp-10.1177_00207640211036174 for Insight and equality: A systematic review and meta-analysis of socio-demographic associations by Kevin Ariyo, Alex Ruck Keene, Anthony S David and Gareth S Owen in International Journal of Social Psychiatry

## References

[bibr1-00207640211036174] Aalten P. van Valen E. de Vugt M. E. Lousberg R. Jolles J. Verhey F. R. (2006). Awareness and behavioral problems in dementia patients: A prospective study. International Psychogeriatrics, 18(1), 3–17. 10.1017/S1041610205002772 16388704

[bibr2-00207640211036174] Allen N. (2014). Is capacity in sight”? International Journal of Mental Health and Capacity Law, 19, 165.

[bibr3-00207640211036174] Ampalam P. Deepthi R. Vadaparty P. (2012). Schizophrenia - insight, depression: A correlation study. Indian Journal of Psychological Medicine, 34(1), 44–48. 10.4103/0253-7176.96158 22661807 PMC3361842

[bibr4-00207640211036174] Appelbaum P. S. Grisso T. (1988). Assessing patients’ capacities to consent to treatment. New England Journal of Medicine, 319(25), 1635–1638. 3200278 10.1056/NEJM198812223192504

[bibr5-00207640211036174] Arbel R. Koren D. Klein E. Latzer Y. (2013). The neurocognitive basis of insight into illness in anorexia nervosa: A pilot metacognitive study. Psychiatry Research, 209(3), 604–610. 10.1016/j.psychres.2013.01.009 23433946

[bibr6-00207640211036174] Barnett P. Mackay E. Matthews H. Gate R. Greenwood H. Ariyo K. Bhui K. Halvorsrud K. Pilling S. Smith S. (2019). Ethnic variations in compulsory detention under the Mental Health Act: A systematic review and meta-analysis of international data. The Lancet Psychiatry, 6(4), 305–317. 30846354 10.1016/S2215-0366(19)30027-6 PMC6494977

[bibr7-00207640211036174] Berg A. O. Barrett E. A. Nerhus M. Büchman C. Simonsen C. Faerden A. Andreassen O. A. Melle I. (2018). Psychosis: Clinical insight and beliefs in immigrants in their first episode. Early Intervention in Psychiatry, 12(2), 185–192. 10.1111/eip.12297 26663787

[bibr8-00207640211036174] Braw Y. Sitman R. Sela T. Erez G. Bloch Y. Levkovitz Y. (2012). Comparison of insight among schizophrenia and bipolar disorder patients in remission of affective and positive symptoms: Analysis and critique. European Psychiatry, 27(8), 612–618. 21565466 10.1016/j.eurpsy.2011.02.002

[bibr9-00207640211036174] Cairns R. Maddock C. Buchanan A. David A. S. Hayward P. Richardson G. Szmukler G. Hotopf M. (2005). Prevalence and predictors of mental incapacity in psychiatric in-patients. The British Journal of Psychiatry, 187(4), 379–385. 16199799 10.1192/bjp.187.4.379

[bibr10-00207640211036174] Case P. (2016). Dangerous liaisons? Psychiatry and law in the court of protection—expert discourses of ‘insight’ (and ‘compliance’). Medical Law Review, 24(3), 360–378. 28007808 10.1093/medlaw/fww027 PMC5178320

[bibr11-00207640211036174] Castrillo Sanz A. Andrés Calvo M. Repiso Gento I. Izquierdo Delgado E. Gutierrez Ríos R. Rodríguez Herrero R. Rodríguez Sanz F. Tola-Arribas M. A . (2016). Anosognosia in Alzheimer disease: Prevalence, associated factors, and influence on disease progression. Neurologia, 31(5), 296–304. 10.1016/j.nrl.2015.03.006 25976940

[bibr12-00207640211036174] Chesnel C. Jourdan C. Bayen E. Ghout I. Darnoux E. Azerad S. Charanton J. Aegerter P. Pradat-Diehl P. Ruet A. Azouvi P. Vallat-Azouvi C. (2018). Self-awareness four years after severe traumatic brain injury: Discordance between the Patient’s and Relative’s complaints. Results from the PariS-TBI study. Clinical Rehabilitation, 32(5), 692–704. 10.1177/0269215517734294 28982252

[bibr13-00207640211036174] Cohen P. Cohen P. West S. G. Aiken L. S. (2014). Applied multiple regression/correlation analysis for the behavioral sciences. Psychology Press.

[bibr14-00207640211036174] Committee on the Rights of Persons with Disabilities. (2018). General comment no. 6 (2018) on equality and non-discrimination. https://ec.europa.eu/info/sites/info/files/combatting_disabiliy_discrimination.pdf

[bibr15-00207640211036174] David A. van Os J. Jones P. Harvey I. Foerster A. Fahy T. (1995). Insight and psychotic illness. Cross-sectional and longitudinal associations. The British Journal of Psychiatry, 167(5), 621–628. 10.1192/bjp.167.5.621 8564318

[bibr16-00207640211036174] David A. S. (1990). Insight and psychosis. The British Journal of Psychiatry, 156, 798–808. 10.1192/bjp.156.6.798 2207510

[bibr17-00207640211036174] David A. S. (2020). Insight and psychosis: The next 30 years. The British Journal of Psychiatry, 217, 521–523. 31685039 10.1192/bjp.2019.217

[bibr18-00207640211036174] de Assis da Silva R. Mograbi D. C. Silveira L. A. Nunes A. L. Novis F. D. Landeira-Fernandez J. Cheniaux E . (2015). Insight across the different mood states of bipolar disorder. Psychiatric Quarterly, 86(3), 395–405. 10.1007/s11126-015-9340-z 25597029

[bibr19-00207640211036174] De Carolis A. Cipollini V. Corigliano V. Comparelli A. Sepe-Monti M. Orzi F. Ferracuti S. Giubilei F . (2015). Anosognosia in people with cognitive impairment: Association with cognitive deficits and behavioral disturbances. Dementia and Geriatric Cognitive Disorders Extra, 5(1), 42–50. 10.1159/000367987 25852731 PMC4361910

[bibr20-00207640211036174] Department for Health and Social Care. (2018). Modernising the mental health act: Increasing choice, reducing compulsion. https://www.gov.uk/government/publications/modernising-the-mental-health-act-final-report-from-the-independent-review

[bibr21-00207640211036174] Dias V. V. Brissos S. Carita A. I. (2008). Clinical and neurocognitive correlates of insight in patients with bipolar I disorder in remission. Acta Psychiatrica Scandinavica, 117, 28–34. 17970840 10.1111/j.1600-0447.2007.01110.x

[bibr22-00207640211036174] Díaz-Caneja C. M. Pina-Camacho L. Rodríguez-Quiroga A. Fraguas D. Parellada M. Arango C. (2015). Predictors of outcome in early-onset psychosis: A systematic review. npj Schizophrenia, 1(1), 14005–14010. 27336027 10.1038/npjschz.2014.5 PMC4849440

[bibr23-00207640211036174] Dourado M. C. N. Laks J. Mograbi D. C. (2019). Awareness in dementia. Alzheimer Disease and Associated Disorders, 33(3), 220–225. 10.1097/wad.0000000000000306 30958416

[bibr24-00207640211036174] Dukart J. Mueller K. Villringer A. Kherif F. Draganski B. Frackowiak R. Schroeter M. L. (2013). Relationship between imaging biomarkers, age, progression and symptom severity in Alzheimer’s disease. NeuroImage Clinical, 3, 84–94. 24179852 10.1016/j.nicl.2013.07.005 PMC3791277

[bibr25-00207640211036174] Fennig S. Everett E. Bromet E. J. Jandorf L. Fennig S. R. Tanenberg-Karant M. Craig T. J. (1996). Insight in first-admission psychotic patients. Schizophrenia Research, 22(3), 257–263. 9000323 10.1016/s0920-9964(96)00077-1

[bibr26-00207640211036174] Fu Y.-N. Cao X.-L. Hou C.-L. Ng C. H. Ungvari G. S. Chiu H. F. K. Lin Y. Q. Wang L. Zheng X. Jia F. J. Xiang Y. T. (2017). Comparison of insight and clinical variables in homeless and non-homeless psychiatric inpatients in China. Psychiatry Research, 255, 13–16. 28505468 10.1016/j.psychres.2017.04.066

[bibr27-00207640211036174] Garg R. Cheema S. K. Raj R. (2018). Psychometric properties of the insight in psychosis questionnaire and its correlation to psychopathology in Indian population. Indian Journal of Psychological Medicine, 40(2), 113–120. 10.4103/IJPSYM.IJPSYM_112_17 29962566 PMC6009001

[bibr28-00207640211036174] Gilleen J. Greenwood K. Archer N. Lovestone S. David A. S. (2012). The role of premorbid personality and cognitive factors in awareness of illness, memory, and behavioural functioning in Alzheimer’s disease. Cognitive Neuropsychiatry, 17(3), 227–245. 10.1080/13546805.2011.588007 21929281

[bibr29-00207640211036174] Goldberg R. W. Green-Paden L. D. Lehman A. F. Gold J. M. (2001). Correlates of insight in serious mental illness. The Journal of Nervous and Mental Disease, 189(3), 137–145. 11277349 10.1097/00005053-200103000-00001

[bibr30-00207640211036174] Gurbai S. Fitton E. Martin W. (2020). Insight under scrutiny in the Court of Protection: A case law survey. Frontiers in Psychiatry, 11, 560329. 33061918 10.3389/fpsyt.2020.560329 PMC7518213

[bibr31-00207640211036174] Heinrichs D. W. Cohen B. P. Carpenter Wt Jr. (1985). Early insight and the management of schizophrenic decompensation. The Journal of Nervous and Mental Disease, 173(3), 133–138. 2857763 10.1097/00005053-198503000-00001

[bibr32-00207640211036174] Higgins J. P. Thompson S. G. Deeks J. J. Altman D. G. (2003). Measuring inconsistency in meta-analyses. BMJ, 327(7414), 557–560. 12958120 10.1136/bmj.327.7414.557 PMC192859

[bibr33-00207640211036174] Johnston C. Liddle J. (2007). The Mental Capacity Act 2005: A new framework for healthcare decision making. Journal of Medical Ethics, 33(2), 94–97. 17264196 10.1136/jme.2006.016972 PMC2598235

[bibr34-00207640211036174] Kalbe E. Salmon E. Perani D. Holthoff V. Sorbi S. Elsner A. Weisenbach S. Brand M. Lenz O. Kessler J. Luedecke S. Ortelli P. Herholz K. (2005). Anosognosia in very mild Alzheimer’s disease but not in mild cognitive impairment. Dementia and Geriatric Cognitive Disorders, 19(5-6), 349–356. 10.1159/000084704 15802909

[bibr35-00207640211036174] Kazui H. Hirono N. Hashimoto M. Nakano Y. Matsumoto K. Takatsuki Y. Mori E. Ikejiri Y. Takeda M. (2006). Symptoms underlying unawareness of memory impairment in patients with mild Alzheimer’s disease. Journal of Geriatric Psychiatry and Neurology, 19(1), 3–12. 10.1177/0891988705277543 16449753

[bibr36-00207640211036174] Kelly B. D. Clarke M. Browne S. McTigue O. Kamali M. Gervin M. Kinsella A. Lane A. Larkin C. O'Callaghan E . (2004). Clinical predictors of admission status in first episode schizophrenia. European Psychiatry, 19(2), 67–71. 15051104 10.1016/j.eurpsy.2003.07.009

[bibr37-00207640211036174] Kim J. S. Park B. K. Kim G. J. Kim S. S. Jung J. G. Oh M. K. Oh J. K. (2007). The role of alcoholics’ insight in abstinence from alcohol in male Korean alcohol dependents. Journal of Korean Medical Science, 22, 132–137. 17297266 10.3346/jkms.2007.22.1.132 PMC2693550

[bibr38-00207640211036174] Klaas H. S. Clémence A. Marion-Veyron R. Antonietti J. P. Alameda L. Golay P. Conus P. (2017). Insight as a social identity process in the evolution of psychosocial functioning in the early phase of psychosis. Psychological Medicine, 47(4), 718–729. 10.1017/S0033291716002506 27866482 PMC5426321

[bibr39-00207640211036174] Kortte K. B. McWhorter J. W. Pawlak M. A. Slentz J. Sur S. Hillis A. E. (2015). Anosognosia for hemiplegia: The contributory role of right inferior frontal gyrus. Neuropsychology, 29(3), 421–432. 10.1037/neu0000135 25133319 PMC4333109

[bibr40-00207640211036174] Lacerda I. B. Santos R. L. Belfort T. Neto J. P. S. Dourado M. C. N. (2020). Patterns of discrepancies in different objects of awareness in mild and moderate Alzheimer’s disease. Aging & Mental Health, 24, 789–796. 10.1080/13607863.2018.1544219 30474400

[bibr41-00207640211036174] Landis J. R. Koch G. G. (1977). The measurement of observer agreement for categorical data. Biometrics, 33, 159–174. 843571

[bibr42-00207640211036174] Liu J. Abdin E. Vaingankar J. A. Shafie S. B. Jeyagurunathan A. Shahwan S. Magadi H. Ng L. L. Chong S. A. Subramaniam M. (2017). The relationship among unawareness of memory impairment, depression, and dementia in older adults with memory impairment in Singapore. Psychogeriatrics, 17(6), 430–438. 10.1111/psyg.12270 28580705

[bibr43-00207640211036174] Lysaker P. H. Bell M. D. Bryson G. J. Kaplan E. (1998). Insight and interpersonal function in schizophrenia. The Journal of Nervous and Mental Disease, 186(7), 432–436. 9680045 10.1097/00005053-199807000-00008

[bibr44-00207640211036174] Marková I. (2005). Insight in psychiatry. Cambridge University Press.

[bibr45-00207640211036174] Martyr A. Clare L. Nelis S. M. Marková I. S. Roth I. Woods R. T. Whitaker C. J. Morris R. G. (2012). Verbal fluency and awareness of functional deficits in early-stage dementia. Clinical Neuropsychologist, 26(3), 501–519. 10.1080/13854046.2012.665482 22394254

[bibr46-00207640211036174] Mayelle A. El Haj M. Antoine P. (2019). Awareness of self and disease assessment: Development and validation of a subjective measure in people with Alzheimer’s disease. Journal of Alzheimer's Disease, 71(3), 841–850. 10.3233/JAD-190371 31450499

[bibr47-00207640211036174] McDaniel K. D. Edland S. D. Heyman A. (1995). Relationship between level of insight and severity of dementia in Alzheimer disease. CERAD clinical investigators. Consortium to establish a registry for Alzheimer’s disease. Alzheimer Disease and Associated Disorders, 9(2), 101–104. 7662321 10.1097/00002093-199509020-00007

[bibr48-00207640211036174] McEvoy J. P. Johnson J. Perkins D. Lieberman J. A. Hamer R. M. Keefe R. S. Tohen M. Glick I. D. Sharma T. (2006). Insight in first-episode psychosis. Psychological Medicine, 36(10), 1385–1393. 10.1017/S0033291706007793 16740175

[bibr49-00207640211036174] Mishra D. K. Alreja S. Sengar K. S. Singh A. R. (2009). Insight and its relationship with stigma in psychiatric patients. Industrial Psychiatry Journal, 18(1), 39. 21234161 10.4103/0972-6748.57858 PMC3016698

[bibr50-00207640211036174] Mograbi D. C. Ferri C. P. Sosa A. L. Stewart R. Laks J. Brown R. Morris R. G. (2012). Unawareness of memory impairment in dementia: A population-based study. International Psychogeriatrics, 24(6), 931–939. 10.1017/S1041610211002730 22251835

[bibr51-00207640211036174] Mohamed S. Rosenheck R. McEvoy J. Swartz M. Stroup S. Lieberman J. A. (2009). Cross-sectional and longitudinal relationships between insight and attitudes toward medication and clinical outcomes in chronic schizophrenia. Schizophr Bull, 35(2), 336–346. 10.1093/schbul/sbn067 18586692 PMC2659303

[bibr52-00207640211036174] Molina-Andreu O. González-Rodríguez A. Villanueva A. P. Penadés R. Catalán R. Bernardo M. (2014). Awareness of illness and suicidal behavior in delusional disorder patients. Revista de Psiquiatria Clinica, 41, 156–158.

[bibr53-00207640211036174] Moore O. Cassidy E. Carr A. O’Callaghan E. (1999). Unawareness of illness and its relationship with depression and self-deception in schizophrenia. Eur Psychiatry, 14(5), 264–269. 10572356 10.1016/s0924-9338(99)00172-8

[bibr54-00207640211036174] NHS Digital. (2020). Mental health act statistics, annual figures 2019-20. https://files.digital.nhs.uk/99/3916C8/ment-heal-act-stat-eng-2019-20-summ-rep%20v1.1.pdf

[bibr55-00207640211036174] Ott B. R. Fogel B. S. (1992). Measurement of depression in dementia: Self vs clinician rating. International Journal of Geriatric Psychiatry, 7(12), 899–904.

[bibr56-00207640211036174] Owen G. S. Freyenhagen F. Richardson G. Hotopf M. (2009). Mental capacity and decisional autonomy: An interdisciplinary challenge. Inquiry, 52(1), 79–107.

[bibr57-00207640211036174] Ozzoude M. Nakajima S. Plitman E. Chung J. K. Kim J. Iwata Y. Caravaggio F. Takeuchi H. Uchida H. Graff-Guerrero A. Gerretsen P. (2019). The effects of illness severity, cognition, and estimated antipsychotic dopamine receptor occupancy on insight into the illness in schizophrenia: An analysis of clinical antipsychotic trials of intervention effectiveness (CATIE) data. Progress in Neuro-Psychopharmacology & Biological Psychiatry, 89, 207–213. 10.1016/j.pnpbp.2018.08.033 30172739

[bibr58-00207640211036174] Poon M. Y. Siu A. M. Ming S. Y. (2010). Outcome analysis of occupational therapy programme for persons with early psychosis. Work, 37(1), 65–70. 20858988 10.3233/WOR-2010-1057

[bibr59-00207640211036174] Pruß L. Wiedl K. H. Waldorf M. (2012). Stigma as a predictor of insight in schizophrenia. Psychiatry Research, 198, 187–193. 22401972 10.1016/j.psychres.2011.12.012

[bibr60-00207640211036174] Rathod S. Kingdon D. Smith P. Turkington D. (2005). Insight into schizophrenia: The effects of cognitive behavioural therapy on the components of insight and association with sociodemographics–data on a previously published randomised controlled trial. Schizophrenia Research, 74(2-3), 211–219. 15722001 10.1016/j.schres.2004.07.003

[bibr61-00207640211036174] Ravi Kishore V. Samar R. Janardhan Reddy Y. C. Chandrasekhar C. R. Thennarasu K . (2004). Clinical characteristics and treatment response in poor and good insight obsessive-compulsive disorder. European Psychiatry, 19(4), 202–208. 10.1016/j.eurpsy.2003.12.005 15196601

[bibr62-00207640211036174] Ruck Keene A. Kane N. B. Kim S. Y. H. Owen G. S . (2019). Taking capacity seriously? Ten years of mental capacity disputes before England’s court of protection. International Journal of Law and Psychiatry, 62, 56–76. 30616855 10.1016/j.ijlp.2018.11.005 PMC6338675

[bibr63-00207640211036174] Schandrin A. Norton J. Raffard S. Aouizerate B. Berna F. Brunel L. Chereau-Boudet I. D'Amato T. Denizot H. Dubertret C. Dubreucq J. Faget C. Fond G. Gabayet F. Llorca P. M. Mallet J. Misdrahi D. Passerieux C. Rey R. . . . Capdevielle D. (2019). A multi-dimensional approach to the relationship between insight and aggressiveness in schizophrenia: Findings from the FACE-SZ cohort. Schizophrenia Research, 204, 38–45. 30082179 10.1016/j.schres.2018.07.029

[bibr64-00207640211036174] Schennach R. Meyer S. Seemüller F. Jäger M. Schmauss M. Laux G. Pfeiffer H. Naber D. Schmidt L. G. Gaebel W. Klosterkötter J. Heuser I. Maier W. Lemke M. R. Rüther E. Klingberg S. Gastpar M. Möller H. J. Riedel M. (2012). Insight in schizophrenia-course and predictors during the acute treatment phase of patients suffering from a schizophrenia spectrum disorder. European Psychiatry, 27(8), 625–633. 10.1016/j.eurpsy.2012.01.001 22542652

[bibr65-00207640211036174] Shaked D. Sunderaraman P. Piscitello J. Cines S. Hale C. Devanand D. Karlawish J. Cosentino S. (2019). Modification of everyday activities and its association with self-awareness in cognitively diverse older adults. PLoS One, 14(11), e0222769. 31697690 10.1371/journal.pone.0222769 PMC6837494

[bibr66-00207640211036174] Sherer M. Hart T. Nick T. G. Whyte J. Thompson R. N. Yablon S. A. (2003). Early impaired self-awareness after traumatic brain injury. Archives of Physical Medicine and Rehabilitation, 84(2), 168–176. 10.1053/apmr.2003.50045 12601646

[bibr67-00207640211036174] Snowden L. R. Hastings J. F. Alvidrez J. (2009). Overrepresentation of Black Americans in psychiatric inpatient care. Psychiatric Services, 60(6), 779–785. 19487347 10.1176/ps.2009.60.6.779

[bibr68-00207640211036174] Solmi M. Radua J. Olivola M. Croce E. Soardo L. Salazar de Pablo G. Il Shin J. Kirkbride J. B. Jones P. Kim J. H. Kim J. Y. Carvalho A. F. Seeman M. V. Correll C. U. Fusar-Poli P. (2021). Age at onset of mental disorders worldwide: Large-scale meta-analysis of 192 epidemiological studies. Molecular Psychiatry, 1–15. Advance Online Publication. 10.1038/s41380-021-01161-7 PMC8960395 34079068

[bibr69-00207640211036174] Spalletta G. Girardi P. Caltagirone C. Orfei M. D. (2012). Anosognosia and neuropsychiatric symptoms and disorders in mild alzheimer disease and mild cognitive impairment. Journal of Alzheimer's Disease, 29(4), 761–772. 10.3233/JAD-2012-111886 22349686

[bibr70-00207640211036174] Spencer B. W. J. Shields G. Gergel T. Hotopf M. Owen G. S. (2017). Diversity or disarray? A systematic review of decision-making capacity for treatment and research in schizophrenia and other non-affective psychoses. Psychological Medicine, 47(11), 1906–1922. 28441976 10.1017/S0033291717000502

[bibr71-00207640211036174] Sterne J. A. Sutton A. J. Ioannidis J. P. Terrin N. Jones D. R. Lau J. Carpenter J. Rücker G. Harbord R. M. Schmid C. H. Tetzlaff J. Deeks J. J. Peters J. Macaskill P. Schwarzer G. Duval S. Altman D. G. Moher D. Higgins J. P. (2011). Recommendations for examining and interpreting funnel plot asymmetry in meta-analyses of randomised controlled trials. BMJ, 343, d4002. 21784880 10.1136/bmj.d4002

[bibr72-00207640211036174] Vigne P. de Menezes G. B. Harrison B. J. Fontenelle L. F. (2014). A study of poor insight in social anxiety disorder. Psychiatry Research, 219(3), 556–561. 10.1016/j.psychres.2014.05.033 24972547

[bibr73-00207640211036174] Walker S. Mackay E. Barnett P. Sheridan Rains L. Leverton M. Dalton-Locke C. Trevillion K. Lloyd-Evans B. Johnson S. (2019). Clinical and social factors associated with increased risk for involuntary psychiatric hospitalisation: A systematic review, meta-analysis, and narrative synthesis. The Lancet Psychiatry, 6(12), 1039–1053. 31777340 10.1016/S2215-0366(19)30406-7 PMC7029280

[bibr74-00207640211036174] Welten C. C. Koeter M. W. Wohlfarth T. D. Storosum J. G. van Den Brink W. Gispen-de Wied C. C. Leufkens H. G. Denys D. A. (2016). Does insight affect the efficacy of antipsychotics in acute mania?: An individual patient data regression meta-analysis. Journal of Clinical Psychopharmacology, 36(1), 71–76. 10.1097/JCP.0000000000000435 26647231

[bibr75-00207640211036174] Wilson R. S. Gilley D. W. Bennett D. A. Beckett L. A. Evans D. A. (2000). Person-specific paths of cognitive decline in Alzheimer’s disease and their relation to age. Psychology and Aging, 15(1), 18. 10755286 10.1037//0882-7974.15.1.18

[bibr76-00207640211036174] Zimmermann N. Mograbi D. C. Hermes-Pereira A. Fonseca R. P. Prigatano G. P. (2017). Memory and executive functions correlates of self-awareness in traumatic brain injury. Cognitive Neuropsychiatry, 22(4), 346–360. 10.1080/13546805.2017.1330191 28566003

