# Supplementary material for: Insight and equality: A systematic review and meta-analysis of socio-demographic associations
Source: Int J Soc Psychiatry. 2021 Aug 4;68(7):1494–506. doi: 10.1177/00207640211036174 (PMC9549177; doi:10.1177/00207640211036174)
Supplement: sj-pdf-1-isp-10.1177_00207640211036174 – Supplemental material for Insight and equality: A systematic review and meta-analysis of socio-demographic associations [file sj-pdf-1-isp-10.1177_00207640211036174.pdf]

1. STUDY CHARACTERISTICS

1.1 Meta-Analysis (Continuous Outcome)

Auth	Year	Country	Measure	Type	N	DV	Cohen's d	Direction	Diagnosis	Hofstede
Bassitt	2007	Brazil	SUMD	Clinician	50	Age	-0.10	Younger age	Schizophrenia	38
Beland	2017	Canada	SAI-E	Clinician	139	Age	0.01	Older age	Schizophrenia	80
Bivona	2008	Italy	AQ-D	Discrepancy	37	Age	0.28	Older age	Brain injury	76
Chapman, Beschin	2019	UK	VAT Amem	Discrepancy	51	Age	-0.02	Younger age	Brain injury	89
Chesnel	2018	France	BICoQ	Discrepancy	90	Age	-0.63	Younger age	Brain injury	71
Cines	2015	USA	ARS	Clinician	103	Age	-0.56	Younger age	Alzheimer's	91
Ciurli	2010	Italy	PCRS	Discrepancy	52	Age	0.02	Older age	Brain injury	76
Clare, Whitaker	2010	UK	MARS	SRAny	80	Age	-0.13	Younger age	Alzheimer's	89
Cuffel	1996	USA	AII	Clinician	89	Age	-0.16	Younger age	Schizophrenia	91
Danki	2007	Turkey	SATCI	Clinician	66	Age	-0.18	Younger age	Schizophrenia	37
DeFeis	2019	USA	Complaint interview	Discrepancy	192	Age	-0.19	Younger age	Alzheimer's	91
Depp	2014	USA	MDIS	SRClinical	106	Age	0.02	Older age	Bipolar disorder	91
Derouesne	1999	France	CDS	Discrepancy	88	Age	-0.45	Younger age	Alzheimer's	71
Diez-Martin	2014	Spain	SUMD	Clinician	161	Age	-0.13	Younger age	Schizophrenia	51
Donohoe, Donnell	2004	Ireland	SAI	Clinician	38	Age	-0.32	Younger age	Schizophrenia	70
Dourado, Laks	2019	Brazil	ASPIDD	Discrepancy	201	Age	0.30	Younger age	Alzheimer's	38
Dourado, Marinho	2007	Brazil	ASPIDD	Discrepancy	52	Age	-0.71	Younger age	Alzheimer's	38
Elvish	2010	UK	OVIS	SRClinical	94	Age	0.24	Older age	OCD	89
Feher	1991	USA	MAC-S	Discrepancy	38	Age	-0.14	Younger age	Alzheimer's	91
Fujimoto	2017	Japan	SRSMF	Discrepancy	49	Age	-0.44	Younger age	Alzheimer's	46
Gerretsen, Chakravarty	2013	Canada	PANSS G12	Clinician	52	Age	-0.24	Younger age	Schizophrenia	80
Gerretsen, Chung	2017	Various	Ecog	SRAny	191	Age	-0.26	Younger age	Alzheimer's	
Gerretsen, Chung	2017	Various	Ecog	SRAny	398	Age	-0.20	Younger age	MCI	
Gerretsen, Chung	2017	Various	Ecog	SRAny	101	Age	0.18	Older age	MCI	
Ghaemi, Stoll	1995	USA	ITAQ	Clinician	28	Age	0.37	Older age	Bipolar disorder	91
Gilleen	2012	UK	*Multiple*		27	Age	-0.58	Younger age	Schizophrenia	89
Hamann	2010	Germany	BIS	SRClinical	300	Age	-0.07	Younger age	Schizophrenia	67
Hannesdottir	2007	Iceland	BAS	Clinician	92	Age	-0.08	Younger age	Alzheimer's	60
Kalbe	2005	Germany	Complaint interview	Discrepancy	79	Age	-0.52	Younger age	MCI	67
Kalbe	2005	Germany	Complaint interview	Discrepancy	82	Age	-0.30	Younger age	Alzheimer's	67
Kao	2010	Taiwan	SAIQ	SRClinical	104	Age	-0.40	Younger age	Schizophrenia	17
Kashyap	2012	India	BABS	SRClinical	150	Age	-0.02	Younger age	OCD	48
Kazui	2006	Japan	EMC	SRAny	103	Age	-0.39	Younger age	Alzheimer's	46
Kelly	2004	Ireland	PANSS G12	Clinician	78	Age	-0.41	Younger age	Schizophrenia	70
Kleim	2008	Various	ITAQ	Clinician	127	Age	-0.10	Younger age	Schizophrenia	
Konstantakopoulos, Ploumpidis	2013	Greece	SAI-E	Clinician	72	Age	-0.30	Younger age	Schizophrenia	35
Lacerda, Belfort	2018	Brazil	ASPIDD	Discrepancy	54	Age	-0.43	Younger age	Alzheimer's	38
Lacerda, Belfort	2018	Brazil	ASPIDD	Discrepancy	74	Age	-0.30	Younger age	Alzheimer's	38
Lacerda, Neto	2017	Brazil	ASPIDD	Discrepancy	89	Age	-0.28	Younger age	Alzheimer's	38
Macpherson	1996	UK	SAI	Clinician	64	Age	-0.39	Younger age	Schizophrenia	89
Martyr, Nelis	2014	UK	FAQ	Discrepancy	100	Age	-0.48	Younger age	Mixed (AD+ Other Dementia)	89
Mayelle	1996	France	ASDA	SRClinical	28	Age	.10	Older age	Alzheimer's	71
McEvoy	2006	USA	ITAQ	Clinician	251	Age	0.32	Older age	Schizophrenia	91

Insight Systematic Review: Appendix

Michel	2013	France	SUMD	Clinician	531	Age	-0.31	Younger age	Schizophrenia	71
Mohamed	2009	USA	ITAQ	Clinician	1432	Age	0.14	Older age	Schizophrenia	91
Molina-Andreu	2014	Brazil	SUMD	Clinician	64	Age	-0.71	Younger age	Schizophrenia	38
Orfei, Varsi	2010	Italy	AQ-D	Discrepancy	38	Age	-1.01	Younger age	Alzheimer's	76
Ozzoude	2019	Canada	PANSS G12	Clinician	373	Age	0.12	Older age	Schizophrenia	80
Schennach	2012	Germany	PANSS G12	Clinician	399	Age	-0.03	Younger age	Schizophrenia	67
Silva	2016	Austria	FAI	Discrepancy	34	Age	-0.24	Younger age	Mixed (MCI + AD)	55
Sitman	2012	Israel	PANSS G12	Clinician	66	Age	-0.68	Younger age	Mixed (Scz + Bipolar)	54
Sousa	2015	Brazil	ASPIDD	Discrepancy	69	Age	-0.19	Younger age	Alzheimer's	38
Spalletta	2012	Italy	AQ-D	Discrepancy	103	Age	-0.60	Younger age	Alzheimer's	76
Spalletta	2012	Italy	AQ-D	Discrepancy	54	Age	-0.16	Younger age	MCI	76
Spalletta	2012	Italy	AQ-D	Discrepancy	52	Age	-0.12	Younger age	MCI	76
Szepietowska	2019	Poland	DEX	Discrepancy	41	Age	-0.50	Younger age	Mixed (Dementia)	
Tolin	2010	USA	Y-BOCS	SRClinical	558	Age	-0.37	Younger age	OCD	91
Tumkaya	2018	Turkey	SAI	Clinician	48	Age	-0.08	Younger age	Schizophrenia	37
Turksoy	2002	Turkey	Unspecified Clinical interview		94	Age	-0.32	Younger age	OCD	37
Vallat-Azouvi	2017	France	BICoQ	Discrepancy	286	Age	-0.14	Younger age	Brain injury	71
Vasterling, Seltzer, Carpenter	1997	USA	PCRS	Discrepancy	55	Age	-0.75	Younger age	Alzheimer’s	91
Vasterling, Seltzer, Carpenter	1997	USA	PCRS	Discrepancy	55	Education	0.65	Better educated	Alzheimer’s	91
Vasterling, Seltzer, Foss	1995	USA	EMQ	Discrepancy	43	Age	-0.26	Younger age	Alzheimer's	91
Verhey	1993	Netherlands	Unspecified Clinical interview		170	Age	-0.70	Younger age		80
Verhulsdonk	2013	Germany	AQ-D	Discrepancy	47	Age	0.10	Older age	Alzheimer's	67
Weiler	2000	USA	ITAQ	Clinician	40	Age	-0.75	Younger age	Bipolar disorder	91
Xiang	2012	China	ITAQ	Clinician	139	Age	-0.02	Younger age	Schizophrenia	20
Yen, Cheng	2008	Taiwan	SAI-E	Clinician	96	Age	-0.07	Younger age	Bipolar disorder	17
Bassitt	2007	Brazil	SUMD	Clinician	50	Education	-0.02	Less educated	Schizophrenia	38
Bladzinski	2019	Poland	MTAF	SRClinical	51	Education	-0.47	Less educated	Schizophrenia	60
Cernovsky	2004	Canada	Unspecified Clinical interview	Clinician	111	Education	0.70	Better educated	Schizophrenia	80
Chapman, Beschin	2019	UK	VATAmem	Discrepancy	51	Education	-0.06	Less educated	Brain injury	89
Danki	2007	Turkey	SATCI	Clinician	66	Education	0.66	Better educated	Schizophrenia	37
DeFeis	2019	USA	Complaint interview	Discrepancy	192	Education	-0.05	Less educated	Alzheimer’s	91
Depp	2014	USA	MDIS	SRClinical	106	Education	0.30	Better educated	Bipolar disorder	91
Diez-Martin	2014	Spain	SUMD	Clinician	161	Education	0.42	Better educated	Schizophrenia	51
Dourado, Laks	2019	Brazil	ASPIDD	Discrepancy	201	Education	0.26	Less educated	Alzheimer’s	38
Elvish	2010	UK	OVIS	SRClinical	94	Education	0.33	Better educated	OCD	89
Feher	1991	USA	MAC-S	Discrepancy	38	Education	0.39	Better educated	Alzheimer's	91
Fujimoto	2017	Japan	SRSMF	Discrepancy	49	Education	0.34	Better educated	Alzheimer's	46
Gerretsen, Chung	2017	Various	Ecog	SRAny	101	Education	-0.22	Less educated	MCI	
Gerretsen, Chung	2017	Various	Ecog	SRAny	398	Education	0.14	Better educated	MCI	
Gerretsen, Chung	2017	Various	Ecog	SRAny	191	Education	0.49	Better educated	Alzheimer's	
Gilleen	2012	UK	*Multiple*		27	Education	0.30	Better educated	Schizophrenia	89
Hamann	2010	Germany	BIS	SRClinical	300	Education	0.01	Better educated	Schizophrenia	67
Kalbe	2005	Germany	Complaint interview	Discrepancy	79	Education	0.20	Better educated	MCI	67
Kalbe	2005	Germany	Complaint interview	Discrepancy	82	Education	-0.26	Better educated	Alzheimer's	67
Kao	2010	Taiwan	SAIQ	SRClinical	104	Education	0.04	Better educated	Schizophrenia	17
Kashyap	2012	India	BABS	SRClinical	147	Education	-0.03	Less educated	OCD	48
Kazui	2006	Japan	EMC	SRAny	103	Education	0.22	Better educated	Alzheimer's	46
Konstantakopoulos, Ploumpidis	2013	Greece	SAI-E	Clinician	72	Education	0.43	Better educated	Schizophrenia	35
Lacerda, Belfort	2018	Brazil	ASPIDD	Discrepancy	74	Education	-0.30	Less educated	Alzheimer's	38
Lacerda, Belfort	2018	Brazil	ASPIDD	Discrepancy	54	Education	0.62	Better educated	Alzheimer's	38
Lacerda, Neto	2017	Brazil	ASPIDD	Discrepancy	89	Education	0.14	Better educated	Alzheimer's	38
Macpherson	1996	UK	SAI	Clinician	64	Education	0.75	Better educated	Schizophrenia	89
Mohamed	2009	USA	ITAQ	Clinician	1432	Education	0.08	Better educated	Schizophrenia	91
Ozzoude	2019	Canada	PANSS G12	Clinician	373	Education	0.22	Better educated	Schizophrenia	80
Rossell	2003	UK	SAI-E	Clinician	78	Education	0.63	Better educated	Schizophrenia	89

Silva	2016	Austria	FAI	Discrepancy	34	Education	-0.16	Less educated	Mixed (MCI + AD)	55
Sousa	2015	Brazil	ASPIDD	Discrepancy	69	Education	0.14	Better educated	Alzheimer's	38
Spalletta	2012	Italy	AQ-D	Discrepancy	54	Education	-0.51	Less educated	MCI	76
Spalletta	2012	Italy	AQ-D	Discrepancy	103	Education	-0.05	Less educated	Alzheimer's	76
Spalletta	2012	Italy	AQ-D	Discrepancy	52	Education	0.01	Better educated	MCI	76
Tumkaya	2018	Turkey	SAI	Clinician	48	Education	-.40	Less educated	Schizophrenia	37
Vallat-Azouvi	2017	France	BICoQ	Discrepancy	286	Education	0.16	Better educated	Brain injury	71
Vasterling, Seltzer, Foss	1995	USA	EMQ	Discrepancy	43	Education	0.37	Better educated	Alzheimer's	91
Verhulsdonk	2013	Germany	AQ-D	Discrepancy	47	Education	0.61	Better educated	Alzheimer's	67
Xiang	2012	China	ITAQ	Clinician	139	Education	-0.06	Less educated	Schizophrenia	20
Yeh	2014	Taiwan	DDS	Discrepancy	36	Education	0.43	Better educated	Alzheimer's	17
Yen, Cheng	2008	Taiwan	SAI-E	Clinician	96	Education	0.42	Better educated	Bipolar disorder	17
Bellino	2005	Italy	OVIS	SRClinical	74	Employment	0.62	Employed	OCD	76
Bladzinski	2019	Poland	*Multiple*		51	Employment	0.71	Employed	Schizophrenia	60
Elvish	2010	UK	OVIS	SRClinical	94	Employment	0.28	Employed	OCD	89
Ghaemi, Stoll	1995	USA	ITAQ	Clinician	28	Employment	0.04	Employed	Bipolar disorder	91
Othman, Huri	2017	Malaysia	ITAQ	Clinician	42	Employment	-0.25	Unemployed	Schizophrenia	46
Schennach	2012	Germany	PANSS G12	Clinician	399	Employment	0.15	Employed	Schizophrenia	67
Xiang	2012	China	ITAQ	Clinician	139	Employment	0.12	Employed	Schizophrenia	20
Elvish	2010	UK	OVIS	SRClinical	94	Ethnicity	0.23	White British	OCD	89
McEvoy	2006	USA	ITAQ	Clinician	226	Ethnicity	0.27	White ethnicity (vs. black)	Schizophrenia	91
Rathod	2005	UK	SAI	Clinician	42	Ethnicity	0.20	White (vs. African-Caribbean)	Schizophrenia	89
Bellino	2005	Italy	OVIS	SRClinical	74	Marital Status	0.22	Married	OCD	76
Depp	2014	USA	MDIS	SRClinical	106	Marital Status	-0.68	Unmarried	Bipolar disorder	91
Elvish	2010	UK	OVIS	SRClinical	94	Marital Status	0.03	Married/Partnership	OCD	89
Garg	2018	India	IP	SRClinical	53	Marital Status	-0.48	Single	Schizophrenia	48
Karow	2008	Germany	SUMD	Clinician	59	Marital Status	0.68	Married/Relationship	Schizophrenia	67
Kelly	2004	Ireland	PANSS G12	Clinician	78	Marital Status	0.61	Married	Schizophrenia	70
Mohamed	2009	USA	ITAQ	Clinician	1432	Marital Status	0.17	Married/Partnership	Schizophrenia	91
Othman, Huri	2017	Malaysia	ITAQ	Clinician	57	Marital Status	0.27	Married	Schizophrenia	26
Rathod	2005	UK	SAI	Clinician	358	Marital Status	0.12	Married	Schizophrenia	89
Schennach	2012	Germany	PANSS G12	Clinician	399	Marital Status	-0.09	Unmarried	Schizophrenia	67
Shimshoni	2011	Israel	DSM-IV Scale		60	Marital Status	0.79	Married/Relationship	OCD	54
Xiang	2012	China	ITAQ	Clinician	139	Marital Status	0.28	Married	Schizophrenia	20
Ampalam	2012	India	SAI	Clinician	60	Sex	0.12	Males	Schizophrenia	48
Bassitt	2007	Brazil	SUMD	Clinician	50	Sex	-0.05	Females	Schizophrenia	38
Bellino	2005	Italy	OVIS	SRClinical	74	Sex	0.12	Males	OCD	76
Chapman, Cosentino	2020	UK	VAT Amem	Discrepancy	35	Sex	-0.67	Females	Stroke	89
Chesnel	2018	France	BICoQ	Discrepancy	90	Sex	-0.40	Females	Brain injury	71
Cobo	2020	Spain	SUMD	Clinician	516	Sex	0.14	Males	Schizophrenia	51
Cuffel	1996	USA	AII	Clinician	89	Sex	0.63	Males	Schizophrenia	91
Derouesne	1999	France	CDS	Discrepancy	88	Sex	-0.17	Females	Alzheimer's	71
Elvish	2010	UK	OVIS	SRClinical	94	Sex	-0.28	Married/Partnership	OCD	89
Fujimoto	2017	Japan	SRSMF	Discrepancy	49	Sex	0.08	Males	Alzheimer's	46
Garg	2018	India	IP	SRClinical	53	Sex	0.34	Males	Schizophrenia	48
Gerretsen, Chakravarty	2013	Canada	PANSS G12	Clinician	52	Sex	0.30	Males	Schizophrenia	80
Ghaemi, Stoll	1995	USA	ITAQ	Clinician	28	Sex	-0.35	Females	Bipolar disorder	91
Hamann	2010	Germany	BIS	SRClinical	300	Sex	-0.02	Females	Schizophrenia	67
Jeong	2017	South Korea	PANSS G12	Clinician	41	Sex	-0.57	Females	Schizophrenia	18
Kao	2010	Taiwan	SAIQ	SRClinical	104	Sex	-0.30	Females	Schizophrenia	17
Kelly	2004	Ireland	PANSS G12	Clinician	78	Sex	-0.25	Females	Schizophrenia	70
McEvoy	2006	USA	ITAQ	Clinician	251	Sex	-0.34	Females	Schizophrenia	91
Michel	2013	France	SUMD	Clinician	531	Sex	-0.07	Females	Schizophrenia	71
Othman, Huri	2017	Malaysia	ITAQ	Clinician	70	Sex	0.39	Males	Schizophrenia	26
Ozzoude	2019	Canada	PANSS G12	Clinician	373	Sex	0.20	Males	Schizophrenia	80
Prus	2012	Germany	SUMD	Clinician	111	Sex	-0.56	Females	Schizophrenia	67
Rathod	2005	UK	SAI	Clinician	422	Sex	0.04	Males	Schizophrenia	89
Schennach	2012	Germany	PANSS G12	Clinician	399	Sex	0.05	Males	Schizophrenia	67
Shimshoni	2011	Israel	DSM-IV Scale	Clinician	60	Sex	-0.67	Females	OCD	54
Xiang	2012	China	ITAQ	Clinician	139	Sex	0.14	Males	Schizophrenia	20
Yen, Cheng	2008	Taiwan	SAI-E	Clinician	96	Sex	0.20	Males	Bipolar disorder	17

Table A.1. Study characteristics of all continuous effect sizes included in the meta-analysis

1.2 Meta-Analysis (Dichotomous Outcome)

Auth	Year	Country	Measure	Type	N	DV	Cohen's d	Variance	Odds	Hofstede	Direction	Diagnosis
Amanzio	2011	Italy	AQ-D	Discrepancy	29	Age	-0.35	0.38	-0.63	76	Younger age	Alzheimer's
Ayesa-Arriola	2014	Spain	SUMD	Clinician	224	Age	-0.19	0.05	-0.35	51	Younger age	Schizophrenia
Chapman, Colvin	2018	USA	CRA	Clinician	35	Age	-0.93	0.33	-1.68	91	Younger age	Alzheimer's
Cherian	2012	India	Y-BOCS	SRClinical	545	Age	0.12	0.06	0.23	48	Older age	OCD
Conde-Sala	2013	Spain	AQ-D	Discrepancy	164	Age	-0.63	0.07	-1.15	51	Younger age	Alzheimer's
Contador	2020	Spain	FAQ	Discrepancy	325	Age	0.07	0.04	0.13	51	Older age	Mixed (AD + Other Dementia)
Cosentino	2007	USA	ARS	Clinician	24	Age	-0.05	0.48	-0.10	91	Younger age	Alzheimer's
Cosentino	2011	USA	ARS	Clinician	42	Age	-0.26	0.26	-0.46	91	Younger age	Alzheimer's
De Berardis	2005	Italy	Y-BOCS	SRClinical	112	Age	0.28	0.12	0.51	76	Older age	OCD
De Berardis	2008	Italy	Y-BOCS	SRClinical	75	Age	0.02	0.17	0.05	76	Older age	OCD
Dias, Brissos, Carita	2007	Portugal	SUMD	Clinician	50	Age	-0.54	0.23	-0.98	27	Younger age	Bipolar disorder
Dias, Brissos, Frey	2008	Portugal	SUMD	Clinician	70	Age	-0.40	0.17	-0.72	27	Younger age	Bipolar disorder
Emami	2016	Canada	SAI-E	Clinician	66	Age	-0.25	0.17	-0.46	80	Younger age	Schizophrenia
Faget	2012	France	SUMD	Clinician	27	Age	0.17	0.40	0.31	71	Older age	Schizophrenia
Fu	2017	China	ITAQ	Clinician	278	Age	-0.42	0.06	-0.76	20	Younger age	Mixed (Psychiatric)
Gambina	2014	Italy	AQ-D	Discrepancy	79	Age	-0.71	0.16	-1.29	76	Younger age	Alzheimer's
Hanyu	2008	Japan	EMC	SRAny	38	Age	-0.13	0.29	-0.23	46	Younger age	Alzheimer's
Himle	2006	USA	Y-BOCS	SRClinical	69	Age	-0.10	0.20	-0.18	91	Younger age	OCD
Jacob	2014	USA	Y-BOCS	SRClinical	130	Age	-0.26	0.14	-0.47	91	Younger age	OCD
Karadag	2011	Turkey	OVIS	SRClinical	64	Age	-0.20	0.18	-0.36	37	Younger age	OCD
Kishore	2004	India	BABS	SRClinical	100	Age	0.19	0.15	0.34	48	Older age	OCD
Kortte	2015	USA	BAS	Clinician	35	Age	-2.63	0.54	-4.76	91	Younger age	Stroke
Lamar	2002	USA	AQ-D	Discrepancy	32	Age	-0.42	0.35	-0.76	91	Younger age	Mixed (AD + Other Dementia)
Loebel	1990	USA	Unspecified Clinical interview	NA	32	Age	-0.43	0.34	-0.79	91	Younger age	Alzheimer's
Lopez, Becker	1994	USA	Unspecified Clinical interview	NA	177	Age	-0.36	0.08	-0.65	91	Younger age	Alzheimer's
Lysaker, Bryson	1998	USA	SUMD	Clinician	101	Age	-0.27	0.11	-0.50	91	Younger age	Schizophrenia
Maeshima	1997	Japan	Unspecified Clinical interview	NA	50	Age	0.75	0.30	1.36	46	Older age	Brain injury
Moro	2016	Various	BAS	Clinician	63	Age	-0.55	0.21	-0.99	NA	Younger age	Stroke
Noe	2005	Spain	PCRS	Discrepancy	62	Age	0.00	0.18	0.00	51	Equal	Brain injury
Onen	2013	Turkey	Y-BOCS	SRClinical	100	Age	0.36	0.11	0.66	37	Older age	OCD
Ozkiris	2015	Turkey	OVIS	SRClinical	63	Age	-0.23	0.20	-0.41	37	Younger age	OCD
Sedaghat	2010	Greece	Unspecified Clinical interview	NA	21	Age	0.27	0.53	0.50	35	Older age	Alzheimer's
Sedaghat	2010	Greece	Unspecified Clinical interview	NA	21	Age	-0.71	0.56	-1.29	35	Younger age	Alzheimer's
Senturk	2017	Turkey	CIRS	Clinician	26	Age	-1.70	0.48	-3.08	37	Younger age	MCI
Senturk	2017	Turkey	CIRS	Clinician	21	Age	-0.26	0.52	-0.48	37	Younger age	Alzheimer's
Shad	2004	USA	BPRS	Clinician	35	Age	-.017	0.31	-0.32	91	Younger age	Schizophrenia
Starkstein, Brockman	2010	Argentina	AQ-D	Discrepancy	77	Age	0.02	0.15	0.04	46	Older age	Alzheimer's
Starkstein, Jorge	2006	Argentina	AQ-D	Discrepancy	173	Age	0.02	0.19	0.04	46	Older age	Alzheimer's
Therriault	2018	Various	Ecog	SRAny	468	Age	-0.34	0.02	-0.61	NA	Younger age	MCI
Tordesillas	2018	Spain	SUMD	Clinician	108	Age	0.27	0.11	0.49	51	Older age	Schizophrenia
Tremont	2011	USA	Unspecified Clinical interview	NA	65	Age	-0.51	0.17	-0.93	91	Younger age	MCI
Turro-Garriga	2013	Spain	ERS	Clinician	124	Age	0.09	0.12	0.17	51	Older age	Alzheimer's
Turro-Garriga	2016	Spain	AQ-D	Discrepancy	177	Age	-0.64	0.07	-1.16	51	Younger age	Alzheimer's
Valiente	2011	Spain	PANSS G12	Clinician	40	Age	-0.33	0.27	-0.60	51	Younger age	Schizophrenia
Wang	2011	China	ITAQ	Clinician	139	Age	-0.14	0.11	-0.24	20	Younger age	Schizophrenia
Woon	2020	India	MDIS	SRClinical	99	Age	-0.55	0.22	-1.00	48	Younger age	Depression
Yoon	2017	South Korea	Unspecified Clinical interview	NA	616	Age	-0.24	0.04	-0.43	18	Younger age	Alzheimer's
Zhang	2016	China	PANSS G12	Clinician	56	Age	-1.48	0.21	-2.69	20	Younger age	Schizophrenia
Amanzio	2011	Italy	AQ-D	Discrepancy	29	Education	0.51	0.38	0.93	76	Better educated	Alzheimer's
Ayesa-Arriola	2014	Spain	SUMD	Clinician	198	Education	0.23	0.06	0.41	51	Better educated	Schizophrenia
Bota	2006	USA	SUMD	Clinician	24	Education	1.14	0.49	2.07	91	Better educated	Schizophrenia
Chapman, Colvin	2018	USA	CRA	Clinician	35	Education	0.42	0.32	0.76	91	Better educated	Alzheimer's
Cosentino	2007	USA	ARS	Clinician	24	Education	-1.07	0.51	-1.95	91	Less educated	Alzheimer's
Cosentino	2011	USA	ARS	Clinician	42	Education	1.07	0.28	1.93	91	Better educated	Alzheimer's
Dias, Brissos, Carita	2007	Portugal	SUMD	Clinician	50	Education	0.66	0.23	1.20	27	Better educated	Bipolar disorder
Dias, Brissos, Frey	2008	Portugal	SUMD	Clinician	70	Education	0.44	0.17	0.79	27	Better educated	Bipolar disorder

Insight Systematic Review: Appendix

Auth	Year	Country	Measure	Type	N	DV	Cohen’s d	Variance	Odds	Hofstede	Direction	Diagnosis
Emami	2016	Canada	SAI-E	Clinician	66	Education	0.06	0.17	0.10	80	Better educated	Schizophrenia
Fu	2017	China	ITAQ	Clinician	278	Education	0.19	0.06	0.35	20	Better educated	Mixed (Psychiatric)
Hanyu	2008	Japan	EMC	SRAny	38	Education	0.42	0.29	0.76	46	Better educated	Alzheimer's
Karadag	2011	Turkey	OVIS	SRClinical	64	Education	-0.33	0.18	-0.60	37	Less educated	OCD
Kishore	2004	India	BABS	SRClinical	100	Education	-0.05	0.15	-0.10	48	Less educated	OCD
Kortte	2015	USA	BAS	Clinician	31	Education	0.04	0.44	0.07	91	Better educated	Stroke
Lamar	2002	USA	AQ-D	Discrepancy	32	Education	0.63	0.35	1.14	91	Better educated	Mixed (AD + Other Dementia)
Loebel	1990	USA	Unspecified Clinical interview	NA	32	Education	0.81	0.35	1.47	91	Better educated	Alzheimer's
Lopez, Becker	1994	USA	Unspecified Clinical interview	NA	177	Education	0.57	0.09	1.03	91	Better educated	Alzheimer's
Lysaker, Bryson	1998	USA	SUMD	Clinician	101	Education	-0.09	0.11	-0.16	91	Less educated	Schizophrenia
Noe	2005	Spain	PCRS	Discrepancy	62	Education	0.04	0.18	0.08	51	Better educated	Brain injury
Ozkiris	2015	Turkey	OVIS	SRClinical	63	Education	-0.36	0.20	-0.66	37	Less educated	OCD
Senturk	2017	Turkey	CIRS	Clinician	26	Education	0.23	0.42	0.42	37	Better educated	MCI
Senturk	2017	Turkey	CIRS	Clinician	21	Education	0.57	0.53	1.03	37	Better educated	Alzheimer's
Starkstein, Brockman	2010	Argentina	AQ-D	Discrepancy	77	Education	0.05	0.15	0.10	46	Better educated	Alzheimer's
Starkstein, Jorge	2006	Argentina	AQ-D	Discrepancy	173	Education	0.11	0.19	0.21	46	Better educated	Alzheimer's
Therriault	2018	Various	Ecog	SRAny	468	Education	0.04	0.02	0.08	NA	Better educated	MCI
Tordesillas	2018	Spain	SUMD	Clinician	108	Education	0.59	0.11	1.07	51	Better educated	Schizophrenia
Tremont	2011	USA	Unspecified Clinical interview	NA	65	Education	-0.25	0.17	-0.46	91	Less educated	MCI
Wang	2011	China	ITAQ	Clinician	139	Education	-0.30	0.11	-0.55	20	Less educated	Schizophrenia
Yoon	2017	South Korea	Unspecified Clinical interview	NA	616	Education	0.33	0.04	0.59	18	Better educated	Alzheimer's
Zhang	2016	China	PANSS G12	Clinician	56	Education	-0.48	0.20	-0.88	20	Less educated	Schizophrenia
Visser	2017	Netherlands	OVIS	SRClinical	140	Employment	0.55	0.09	1.00	80	Employed	OCD
Wang	2011	China	ITAQ	Clinician	139	Employment	0.23	0.10	0.41	20	Employed	Schizophrenia
Woon	2020	India	MDIS	SRClinical	99	Employment	-0.38	0.26	-0.69	48	Unemployed	Depression
Heinrichs	1985	USA	Clinical judgement	NA	37	Ethnicity	0.66	0.42	1.19	81	White	Schizophrenia
Kortte	2015	USA	BAS	Clinician	35	Ethnicity	-0.65	0.45	-1.17	81	African American	Stroke
Lysaker, Bryson	1998	USA	SUMD	Clinician	96	Ethnicity	0.29	0.11	0.52	81	White	Schizophrenia
Cherian	2012	India	Y-BOCS (item 11)	SRClinical	536	Marital Status	0.02	0.05	0.04	48	Married	OCD
Fennig	1996	USA	HDS	Clinician	229	Marital Status	0.86	0.08	1.56	81	Married	Schizophrenia (+ other psychoses)
Fu	2017	China	ITAQ	Clinician	278	Marital Status	0.43	0.06	0.78	20	Married	Mixed (Psychiatric)
Karadag	2011	Turkey	OVIS	SRClinical	62	Marital Status	0.24	0.15	0.43	37	Married	OCD
Kishore	2004	India	BABS	Clinician	100	Marital Status	-0.48	0.14	-0.86	48	Unmarried	OCD
Turksoy	2002	Turkey	DSM-IV	NA	88	Marital Status	0.01	0.13	0.02	37	Married	OCD
Woon	2020	India	MDIS	SRClinical	99	Marital Status	-0.61	0.26	-1.10	48	Unmarried	Depression
Amanzio	2011	Italy	AQ-D	Discrepancy	29	Sex	-0.01	0.31	-0.01	76	Female	Alzheimer's
Ayesa-Arriola	2014	Spain	SUMD	Clinician	224	Sex	-0.22	0.04	-0.40	51	Female	Schizophrenia
Baier	2005	Germany	BAS	Clinician	128	Sex	0.06	0.10	0.11	67	Male	Stroke
Castrillo Sanz	2016	Spain	CIR	Clinician	127	Sex	-0.36	0.13	-0.66	51	Female	Alzheimer's
Chapman	2018	USA	CRA	Clinician	35	Sex	0.08	0.30	0.15	81	Male	Alzheimer's
Cherian	2012	India	Y-BOCS	SRClinical	545	Sex	0.14	0.05	0.25	48	Male	OCD
Conde-Sala	2013	Spain	AQ-D	Discrepancy	164	Sex	0.02	0.06	0.04	51	Male	Alzheimer's
Contador	2020	Spain	FAQ	Discrepancy	325	Sex	-0.26	0.03	-0.47	51	Female	Mixed (AD + Other Dementia)
Cosentino	2007	USA	ARS	Clinician	24	Sex	-0.61	0.43	-1.10	81	Female	Alzheimer's
Cosentino	2011	USA	CRA	Clinician	42	Sex	-0.01	0.21	-0.02	91	Female	Alzheimer's
De Berardis	2005	Italy	Y-BOCS (item 11)	Clinician	123	Sex	0.03	0.10	0.06	76	Male	OCD
De Berardis	2008	Italy	Y-BOCS (item 11)	Clinician	75	Sex	0.06	0.14	0.11	76	Male	OCD
De Carolis	2015	Italy	CIRS	Clinician	108	Sex	0.01	0.13	0.03	76	Male	MCI
Dias, Brissos, Frey	2008	Portugal	SUMD	Clinician	70	Sex	-0.51	0.17	-0.93	27	Female	Bipolar disorder
Emami	2016	Canada	SAI-E	Clinician	66	Sex	0.18	0.18	0.33	80	Male	Schizophrenia
Faget	2012	France	SUMD	Clinician	31	Sex	-0.94	0.72	-1.71	71	Female	Schizophrenia
Fu	2017	China	ITAQ	Clinician	278	Sex	0.09	0.05	0.16	20	Male	Mixed (Psychiatric)
Hanyu	2008	Japan	EMC	SRAny	38	Sex	0.23	0.24	0.42	46	Male	Alzheimer's
Heinrichs	1985	USA	Clinical judgement	NA	38	Sex	-0.07	0.25	-0.12	81	Female	Schizophrenia
Himle	2006	USA	Y-BOCS (item 11)	Clinician	69	Sex	-0.31	0.16	-0.56	91	Female	OCD
Jacob	2014	USA	Y-BOCS	SRClinical	129	Sex	-0.23	0.12	-0.42	81	Female	OCD
Karadag	2011	Turkey	OVIS	SRClinical	64	Sex	-0.50	0.16	-0.90	37	Female	OCD
Kishore	2004	India	BABS	Clinician	100	Sex	0.28	0.12	0.51	48	Male	OCD
Kortte	2015	USA	BAS	Clinician	35	Sex	-0.04	0.36	-0.07	81	Female	Stroke
Lamar	2002	USA	AQ-D	Discrepancy	32	Sex	-0.09	0.29	-0.16	91	Female	Mixed (AD + Other Dementia)
Loebel	1990	USA	Clinical interview	NA	32	Sex	0.38	0.50	0.69	81	Male	Alzheimer's

Auth	Year	Country	Measure	Type	N	DV	Cohen's d	Variance	Odds	Hofstede	Direction	Diagnosis
Lopez, Becker	1994	USA	Clinical judgement	NA	181	Sex	0.07	0.08	0.13	81	Male	Alzheimer's
Lysaker, Bryson	1998	USA	SUMD	Clinician	101	Sex	-0.39	0.48	-0.70	81	Female	Schizophrenia
Maeshima	1997	Japan	Clinical judgement	NA	50	Sex	-0.18	0.31	-0.33	46	Female	Brain injury
Moro	2016	Various	Structured interview (Berti, Ladavas & Della Corte, 1996)	NA	63	Sex	0.36	0.19	0.65	NA	Male	Stroke
Noe	2005	Spain	PCRS	Discrepancy	62	Sex	0.30	0.18	0.54	51	Male	Brain injury
Onen	2013	Turkey	Y-BOCS	SRClinical	100	Sex	-0.21	0.09	-0.38	37	Female	OCD
Ozkiris	2015	Turkey	OVIS	SRClinical	63	Sex	0.07	0.19	0.12	37	Male	OCD
Senturk	2017	Turkey	CIRS	Clinician	47	Sex	0.05	0.19	0.09	37	Male	Alzheimer's
Starkstein, Brockman	2010	Argentina	AQ-D	Discrepancy	77	Sex	0.12	0.13	0.22	46	Male	Alzheimer's
Starkstein, Sabe	1996	Argentina	AQ-D	Discrepancy	55	Sex	-0.14	0.20	-0.26	46	Female	Alzheimer's
Therriault	2018	Various	Ecog	SRAny	468	Sex	-0.30	0.02	-0.55	NA	Female	MCI
Tordesillas	2018	Spain	SUMD	Clinician	108	Sex	0.06	0.10	0.11	51	Male	Schizophrenia
Tremont	2011	USA	Clinical Judgement	NA	65	Sex	0.01	0.14	0.02	81	Male	MCI
Turksoy	2002	Turkey	DSM-IV	NA	94	Sex	-0.41	0.11	-0.74	37	Female	OCD
Turro-Garriga	2013	Spain	ERS	Discrepancy	124	Sex	0.32	0.13	0.58	51	Male	Alzheimer's
Turro-Garriga	2016	Spain	AQ-D	Clinician	177	Sex	-0.30	0.06	-0.55	51	Female	Alzheimer's
Valiente	2011	Spain	PANSS G12	Clinician	40	Sex	-0.22	0.22	-0.39	51	Female	Schizophrenia
Visser	2017	Netherlands	OVIS	SRClinical	140	Sex	0.21	0.08	0.37	80	Male	OCD
Wang	2011	China	ITAQ	Clinician	139	Sex	-0.13	0.09	-0.23	20	Female	Schizophrenia
Wibawa	2019	Australia	AS	Discrepancy	38	Sex	0.06	0.31	0.12	90	Male	Huntington's
Woon	2020	India	MDIS	SRClinical	99	Sex	-0.10	0.18	-0.18	48	Female	Depression
Yoon	2017	South Korea	Clinician semi-structured interview	NA	617	Sex	-0.33	0.03	-0.60	18	Female	Alzheimer's
Zhang	2016	China	PANSS G12	Clinician	56	Sex	0.32	0.16	0.57	20	Male	Schizophrenia

Table A.2. Study characteristics of all dichotomous effect sizes that were included in the meta-analysis.

1.3 Qualitative Only Studies

First Author	Year	Country	Insight Measure	Study Design	Participant No.	Sociodemographic variable	Journal	Reason for exclusion	Participant Group	Significant outcomes?
Aalten	2006	Netherlands	GRAD	Prospective Cohort	199	Age, education, sex, socioeconomic status	International Psychogeriatrics	Ineligible analyses	Dementia	Yes (age, education, socioeconomic status, sex)
Almeida	1996	Brazil	SAI	Cross Sectional	40	Age,	International Journal of Geriatric Psychiatry	Absent statistics	Paraphrenia	No
Amador	1994	USA	SUMD	Cross Sectional	412	Age, education, sex	Archives of General Psychiatry	Absent statistics	Psychosis and Mood Disorder	No
Arbel	2013	Israel	SUMD	Cross Sectional	25	Age, , education	Psychiatry Research	Few population samples	Anorexia	No
Berg	2018	Norway	BIS	Cross Sectional	80	Immigration Status	Early Intervention in Psychiatry	Ineligible insight variable/demographic	Psychosis	Yes (immigration status)
Bianchini	2014	Italy	SAI	Prospective Cohort	55	Age, sex	Psychiatry Research	Absent statistics	Schizophrenia	No
Buchy	2010	Canada	SUMD	Prospective cohort	165	Age, education, sex	Early Intervention in Psychiatry	Longitudinal only	FEP	No
Burton	2016	USA	MIC-SR vs. neuropsychological test	Cross Sectional	168	Age, education, employment, ethnicity, marital status, sex	Schizophrenia Research	Neuropsychological test discrepancy	Schizophrenia Spectrum Disorders	No
Chan	2014	Hong Kong	SUMD	Prospective cohort	71	Age, education	Psychiatry Research	Longitudinal only	First Episode Schizophrenia	No
Chan	2016	Hong Kong	SUMD	Cross Sectional	95	Age, , education, employment, marital status, sex	Comprehensive Psychiatry	Absent statistics	Schizophrenia Spectrum Disorder	No
Chen	2001	Hong Kong	SUMD	Prospective Cohort	70	Age, education	The Journal of Nervous and Mental Disease	Longitudinal only	Psychiatric Inpatients	No
Chen	2018	Taiwan	PRMQ	Cross Sectional	90	Age, education, sex	Psychiatry Research	Ineligible analysis	Alcohol dependence	Yes (age, education)
Collins	1997	Canada	SAI	Cross Sectional	58	Age	Schizophrenia Research	Ineligible analysis	Schizophrenia	No
Comacchio	2020	Italy	SAI-E	Prospective cohort	185	Sex	Archives of Women's Mental Health	Longitudinal only	FEP	Yes (gender)
Cuesta	1994	Spain	Lack of Insight Index	Cross Sectional	40	Age, education, sex	Schizophrenia Bulletin	Absent statistics	Schizophrenia	No
David	1992	UK	SAI	Cross Sectional	91	Age	British Journal of Psychiatry	Absent statistics	Psychosis	No
David	1995	UK	PSE Insight Item	Prospective Cohort	150	Age, , socioeconomic status, ethnicity, immigration, sex	British Journal of Psychiatry	Absent statistics	Psychosis	Yes (socioeconomic status)
De Assis	2015	Brazil	HAM-D & YMRS	Cross Sectional	48	Age	Psychiatric Quarterly	Ineligible insight variable/demographic	Bipolar disorder	Yes (age, sex)
de Castro Zilli	2007	Brazil	DIS	Cross Sectional	21	Age, education	Dementia & Neuropsychologia	Absent statistics	Alzheimer's disease	No
Duarte Gigante	2004	Brazil	SAI	Cross Sectional	40	Age, , education, sex	Sao Paulo Medical Journal	Absent statistics	Schizophrenia	No
Farias	2005	USA	DFQ	Cross Sectional	111	Age, education, ethnicity, sex	International Journal of Geriatric Psychiatry	Absent statistics	Older adults	No
Gilleen	2011	UK	DEX, MARS, PCRS, SUMD, SAI-E	Cross Sectional	31	Age, education	Schizophrenia Bulletin	Absent statistics	Schizophrenia	No
Goldberg	2001	USA	PANSS G12	Cross Sectional	211	Age, education, ethnicity, sex	The Journal of Nervous and Mental Disease	Ineligible analysis	SMI	Yes (ethnicity)
Gomez-de-Regil	2015	Mexico	SUMD	Cross Sectional	61	Age, , marital status, sex	Schizophrenia Research: Cognition	Absent statistics	Psychosis	No
Greenfeld	1990	USA	Schedule for the Assessment of Insight in Illness (Greenfeld et al, 1989)	Prospective cohort	40	BMI	International Journal of Eating Disorders	Longitudinal only	Anorexia	No
Hanseeuw	2020	Various	ECog	Prospective cohort	1070	Age, education, sex	Annals of Neurology	Longitudinal only	Older adults (including MCI and AD)	Yes (age)

Insight Systematic Review: Appendix

First Author	Year	Country	Insight Measure	Study Design	Participant No.	Sociodemographic variable	Journal	Reason for exclusion	Participant Group	Significant outcomes?
Hoth	2007	USA	PCRS	Cross Sectional	66	Age, education	Journal of Clinical and Experimental Neuropsychology	Absent statistics	Huntington's disease	No
Jong	2007	Korea	HAIS	Cross Sectional	123	Age, education, religion, occupation	Journal of Korean Medical Sciences	Few population samples	Alcohol use disorder	Yes (age)
Kemp, Lambert	1995	UK	SUMD	Cross Sectional	29	Age, marital status	Schizophrenia Research	Absent statistics	Schizophrenia	No
Keshavan	2004	USA	PANSS G12	Cross Sectional	535	Age, , education, sex	Schizophrenia Research	Ineligible analysis	Schizophrenia Spectrum Disorder	No
Kim, Ozzoude	2020	Canada	PANSS G12	Randomised Controlled Trial	1447	Age, education, ethnicity, sex	Neuropsychopharmacology	Ineligible analysis	Schizophrenia	Yes (sex)
Klaas	2017	Switzerland	Clinical Judgement	Prospective Cohort	240	Education, migration status, sex	Psychological Medicine	Absent statistics	EIP	No
Kumar	2013	India	SUMD	Cross Sectional	44	Marital Status, sex	Journal of Postgraduate Medicine	Absent statistics	Mania	No
Liu	2017	Singapore	Subjective vs. Cognitive testing discrepancy	Cross Sectional	751	Marital Status, sex	Psychogeriatrics	Neuropsychological test discrepancy	Memory Impairment in older adults	Yes (marital status, sex)
Lysaker, Bell	1995	USA	PANSS G12	Case Control	44	Age, education, employment	The Journal of Nervous and Mental Disease	Longitudinal only	Schizophrenia	No
Lysaker, Dimaggio	2011	USA	SUMD	Cross Sectional	65	Age, education, sex	Comprehensive Psychiatry	Absent statistics	Schizophrenia Spectrum Disorder	No
Lysaker, Gagen	2018	USA	PANSS G12	Cross Sectional	324	Sex	Schizophrenia Bulletin	Ineligible analysis	Schizophrenia or Schizoaffective disorder	Yes (sex)
Lysaker, Whitney	2006	USA	SUMD	Cross Sectional	53	Age, education	Journal of Neuropsychiatry and Clinical Neurosciences	Absent statistics	Schizophrenia or Schizoaffective disorder	No
Marazziti	2002	Italy	Y-BOCS (item 11)	Cross Sectional	117	Age,	European Psychiatry	Absent statistics	OCD	No
Maremmani	2012	Italy	DAH-RS item 7	Cross Sectional	1066	Age, employment, income, marital status, sex	Frontiers in Psychiatry	Absent statistics	Substance misuse	No
Martyr, Clare	2012	UK	FAD	Cross Sectional	96	Age	The Clinical Neuropsychologist	Ineligible analysis	Dementia	Yes (age)
McCabe	2002	NR	SAI	Cross Sectional	89	Sociodemographic (unspecified)	Journal of Nervous and Mental Disease	Ineligible insight variable/demographic	Schizophrenia	No
Mintz	2004	USA	BIS	Cross Sectional	91	Age	Israel Journal of Psychiatry and Related Sciences	Ineligible analysis	Psychiatric inpatients	No
Mograbi	2012	Various	Self report vs. Neuropsychological test	Cross Sectional	683	Education, marital status, sex	International Psychogeriatrics	Neuropsychological test discrepancy	Dementia	Yes (education)
Moore	1999	Ireland	SUMD	Cross Sectional	46	Age, socioeconomic status, sex	European Psychiatry	Absent statistics	Schizophrenia	No
Mullick	2001	USA	SAI	Cross Sectional	44	Education	Psychiatric Services	Absent statistics	SMI	No
Parellada	2009	Spain	SUMD	Prospective Cohort	110	Age, sex	Psychological Medicine	Absent statistics	FEP (adolescents)	No
Pijnenborg	2015	Various	PANSS G12	Prospective Cohort	455	Age, country, sex	European Neuropsychopharmacology	Ineligible analysis	Schizophrenia and Schizoaffective	No
Pia	2014	Italy	Clinical Judgement	Cross Sectional	27	Age, education	Cortex	Ineligible analysis	Stroke	No
Pillai	2018	USA	AQ-D	Cross Sectional	80	Age, education	Movement Disorders and Clinical Practice	Absent statistics	Parkinson's disease	No
Reed	1993	USA	ARS	Cross Sectional	57	Age, education	Journal of Clinical and Experimental Neuropsychology	Ineligible analysis	Alzheimer's disease	No
Sanchez-Torres	2015	Spain	AMDP	Retrospective Cohort	42	Age, , education, sex	Schizophrenia Research	Longitudinal only	Schizophrenia	Yes (sex)
Sanz	1998	UK	ITAQ, Markova & Berrios (a), Markova & Berrios (b), PANSS G12	Cross Sectional	33	Education	Psychological Medicine	Absent statistics	Psychosis	Yes (education)
Saravanan	2007	India	SAI-E	Cross Sectional	131	Age, education, sex	Social Psychiatry and Psychiatric Epidemiology	Absent statistics	Schizophrenia	No
Sasse	2013	Germany	PCRS	Cross Sectional	141	Age, education, employment	Journal of Head Trauma and Rehabilitation	Absent statistics	Traumatic Brain Injury	No
Schwartz	1997	USA	SUMD	Prospective Cohort	23	Age	Comprehensive Psychiatry	Absent statistics	Schizophrenia	No
Setkowski	2016	Netherlands	BIS	Prospective Cohort	100	Age, country, domestic situation, sex	International Journal of Social Psychiatry	Longitudinal only	Psychiatric Inpatients	No
Sherer	2003	USA	AQ-D	Cross Sectional	129	Age, education, sex	Archives of Physical Medicine Rehabilitation	Ineligible analysis	Traumatic Brain Injury	Yes (age)
Sherer	2005	USA	AQ(2)	Cross Sectional	91	Age	Journal of Head and Trauma Rehabilitation	Ineligible analysis	Traumatic brain injury	No
Smith	1997	USA	SUMD	Cross Sectional	33	Age, sex	Journal of Clinical Psychopharmacology	Absent statistics	Schizophrenia	No
Smith	2014	Australia	YMRS (item 11)	Cross Sectional	41	Age, sex	Journal of Affective Disorders	Ineligible insight variable/demographic	FEP	No
Tariku	2019	Ethiopia	SUMD	Cross Sectional	455	Employment, literacy, sex	Psychiatry Journal	Ineligible analysis	Schizophrenia	Yes (employment)
Turro-Garriga	2014	Spain	AQ-D	Cross Sectional	352	Age, marital status, sex	Journal of Geriatric Psychiatry and Neurology	Absent statistics	Alzheimer's disease	Yes (age)
van Vliet	2013	Netherlands	GRAD	Prospective Cohort	268	Sex	Alzheimer's Disease & Associated Disorders	Longitudinal	Alzheimer's disease	Yes (sex)
Vanderploeg	2007	USA	KBCI	Cross Sectional	36	Age, education	Journal of Rehabilitation and Research Development	Absent statistics	Traumatic brain injury (moderate to severe)	No
Vannini	2017	USA	Discrepancy between neuropsychological testing and MFQ subscale	Cross Sectional	297	Age, education, sex	Neuropsychologia	Neuropsychological test discrepancy	Mild cognitive impairment	Yes (sex)
Vasterling, Seltzer, Watrous	1997	USA	EMQ	Prospective Cohort	28	Age, , education	Neuropsychiatry, Neuropsychology and Behavioural Neurology	Longitudinal only	Alzheimer's disease	No
Vazmalaei, Jolfaei	2012	Iran	MDIS/SUMD	Cross Sectional	145	Age	Journal of Research in Medical Sciences	Absent statistics	Bipolar disorder (type 1)	No
Vigne	2014	Brazil	BABS	Cross Sectional	37	Age, employment, marital status, religion, socioeconomic status, sex	Psychiatry Research	Few population samples	Social anxiety disorder	Yes (marital status)
Welten	2016	Netherlands	YMRS (item 11)	Retrospective Cohort	1904	Age, BMI, sex	Journal of Clinical Psychopharmacology	Ineligible insight variable/demographic	Bipolar disorder (acute mania)	Yes (BMI)
Wiffen	2010	Various	SAI-E	Retrospective Cohort	303	Sex	Schizophrenia Research	Ineligible analysis	Schizophrenia or Schizoaffective disorder	No
Wilson	2015	USA	Discrepancy between episodic memory rating and neuropsychological battery performance	Retrospective Cohort	2092	Age, education, sex	Neurology	Neuropsychological test discrepancy	Dementia	Yes (age)
Yen, Chen.1	2002	Taiwan	SAI-E	Cross Sectional	109	Sex	The Journal of Nervous and Mental Disease	Ineligible analysis	Psychosis	No

First Author	Year	Country	Insight Measure	Study Design	Participant No.	Sociodemographic variable	Journal	Reason for exclusion	Participant Group	Significant outcomes?
Yen, Hslao	2008	Taiwan	HAIS	Cross Sectional	401	Employment, ethnicity, sex	The American Journal of Drug and Alcohol Abuse	Ineligible analysis	Alcohol use disorder	Yes (ethnicity)
Yen, Chen.2	2003	Taiwan	SAI-E	Prospective Cohort	33	Age, education	Comprehensive Psychiatry	Longitudinal only	Bipolar disorder	No
Young	1993	Canada	SUMD	Cross Sectional	31	Age, education	Schizophrenia Research	Absent statistics	Schizophrenia	No
Zimmerman	2017	Brazil	PCRS	Cross Sectional	65	Age	Cognitive Neuropsychiatry	Ineligible analysis	Traumatic brain injury	Yes (age)

Table A.3. Study characteristics of all records that were only included in the qualitative synthesis.

2. SENSITIVITY ANALYSES FOR INFLUENTIAL CASES

2.1 Overview of Potential Outliers: Meta-Analysis (Continuous Outcome)

Variable	k	Original I^2	First Author	Cook's Distance (Critical d)	Leave in/out Effect Size	I^2 Change	Effect Size Change
Age	67	52%	McEvoy	.09 (.06)	-.22	-5%	-.01
			Mohamed	.09 (.06)	-.22	-7%	-.01
			Verhey	.12 (.06)	-.20	-3%	+.01
Education	44	62%	Cernovsky	.11 (.09)	.14	-5%	.0
Marital Status	13	93%	Ampalam	.47 (.31)	.04	-11%	-.10
Sex	27	59%	Prus	.17 (.15)	.03	-7%	-.02

Table A.4. A summary of influential cases for each socio-demographic characteristic when insight was measured as a continuous variable. Excluded cases are highlighted in red.

2.2 Overview of Potential Outliers: Meta-Analysis 2 (Dichotomous Outcome)

Variable	k	First Author	Original I^2	Cook's Distance (Critical d)	Leave in/out Effect Size	I^2 Change	Effect Size Change
Age	48	De Carolis	91%	1.47 (.08)	.24	-86%	-.22
Education	31	De Carolis	63%	.43 (.13)	.18	-63%	+.06
	30	Therriault	0%	.16 (.13)	.19	0%	+ 02
Sex	49	Therriault	13%	.15 (.08)	.13	-6%	-.02
		Yoon	13%	.11 (.08)	.13	-3%	-.02

Table A.5. A summary of influential cases for each socio-demographic characteristic when insight was measured as a dichotomous variable. Excluded cases are highlighted in red.

2.3 Overview of Meta-Analytic Decisions

Variable	First Author	Methodological Considerations	Considerations for Meta-Analysis	Study Author Comments	Decision to Exclude?
Age	De Carolis	Very low threshold for 'anosognosia' group (score >1). Large heterogeneity observed for other variables in study. Mean age = 72.5.	Large heterogeneity in age. Large number of studies looking at age. Study effect size is very large and significant, and the direction is comparable to other effect sizes in MCI groups. Cook's d is minimally above threshold. Removal would substantially change the pooled effect size and substantially reduce heterogeneity.	None.	Yes.
	McEvoy	Large schizophrenia sample (n= 251). Mean age= 40.52. Longitudinal, randomised trial for antipsychotics.	Moderate heterogeneity in age. Large number of studies looking at age. Study effect size is moderate and significant. In opposite direction, which may be expected, as the effect is weaker in schizophrenia samples. Cook's d is minimally above threshold. Removal would not substantially change the pooled effect size or heterogeneity. Pooled effect would remain significant if removed.	Reported but not explained.	No.
	Mohamed	Very large, chronic schizophrenia sample (n= 1432). Mean age = 40.5. Longitudinal, randomised trial for antipsychotics.	Moderate heterogeneity in age. Large number of studies looking at age. Study effect size is small and significant. In opposite direction, which may be expected, as the effect is weaker in schizophrenia samples. Cook's d is minimally above threshold. Removal would not substantially change the pooled effect size or heterogeneity. Pooled effect would remain significant if removed.	None.	No.
	Verhey	Dutch study. Large, mixed dementia sample. Awareness scored with dual raters and achieved reasonably good reliability. Mean age= 71.2.	Moderate heterogeneity in age. Large number of studies looking at age. Study effect size is large and significant. In expected direction, and is comparable to other effect sizes in dementia groups. Cook's d is moderately above threshold. Removal would not substantially change the pooled effect size or heterogeneity. Pooled effect would remain significant if removed.	Reported but not explained.	No.
Education	Cernovsky	Canadian study. Moderate size schizophrenia sample (n= 111). Mean age= 38.10. Insight assessed using generic clinical interview and then dichotomised.	Moderate heterogeneity in education. Large number of studies looking at education. Study effect size large and significant and in the expected direction. Cook's d is minimally above threshold. Removal would not change the pooled effect size to or substantially reduce heterogeneity. Pooled effect would remain significant if removed.	Reported but not explained.	No.
	De Carolis	Very low threshold for 'anosognosia' group (score >1). Large heterogeneity observed for other variables in this study. Mean education = 9.3. All participants had at least five years of education so potentially biased.	Moderate heterogeneity in education. Large number of studies looking at education. Study effect size not large or significant but only one other study found an effect in this direction. Cook's d very high. Removal would moderately change the pooled effect size and would substantially reduce heterogeneity.	None.	Yes.
	Therriault	Amnestic MCI sample. Multiple countries. 46% female. 44% sample had insight. Cut-off determined statistically. Large sample (N= 467). Mean age= 71.29.	Zero heterogeneity in education. Large number of studies looking at education. Study level effect is minimal and non-significant. Cook's d is minimally above threshold. Would not substantially change the effect size or heterogeneity to remove. Pooled effect would remain significant if removed.	Reported but not explained.	No.
Marital Status	Ampalam	Indian study. Small-to-moderate sample size (N= 60). Mean age not reported. 46% male sample overall. SAI scale used to assess insight.	Large heterogeneity in marital status. Relatively small number of studies looking at marital status. Study effect size is large and significant but in opposite direction to the pooled effect. Cook's d moderately high. Would substantially change the pooled effect size and heterogeneity to remove. Pooled effect would remain non-significant if removed.	Reported and explanation offered (coping mechanisms and social support).	Yes.
Sex	Prus	German study. Moderate sample size (n= 111). Insight rated using SUMD scale and administered by graduate students. Mean age= 39. 53% male.	Moderate heterogeneity in sex. Moderate number of studies looking at education. Study effect size is large and significant. Cook's d minimally above threshold. Would moderately change the pooled effect size to remove and would substantially reduce heterogeneity. Pooled effect would remain non-significant if removed.	Reported and explanations offered (gender differences in disclosure, verbal memory, adaptive behaviour, neurobiological differences)	No.
	Therriault	Amnestic MCI sample. Multiple countries. 46% female. 62.6% sample had insight. Cut-off determined statistically. Large sample (N= 467). Mean age= 71.29.	Very little heterogeneity in sex. Large number of studies looking at sex. Study effect in same direction to pooled effect. Cook's d moderately above threshold. Would not substantially change pooled effect size or heterogeneity to remove. However, pooled effect would become marginally non-significant if removed (p= .067)	Reported but not explained.	No.
	Yoon	Very large sample (N= 616). South Korean study. Patients diagnosed with early onset Alzheimer's disease. 87.6% of patients had insight. 37% male.	Very little heterogeneity in sex. Large number of studies looking at sex. Study effect in same direction to pooled effect. Cook's d minimally above threshold. Would not substantially change pooled effect size or heterogeneity to remove. However, pooled effect would become marginally non-significant if removed (p= .062)	None.	No.

Table A.6. Study characteristics and inclusion decisions for potential outliers.

2.4 Data analysis procedure

All statistical results were converted into standardised effect sizes (cohen's d and fisher's z), to correct for potential violations of the normality assumption. The procedure for this is outlined below. All effects were ultimately reported as Cohen's d, following standard research reporting procedures. 95% confidence intervals were also calculated for each effect size. 3x2 Chi Squared tests were excluded, unless they could be reasonably reduced to a two-by-two frequency matrix. This was done for marital status (single/unmarried vs. married/partnered), ethnicity (white/Caucasian ethnicity vs. black/African/Caribbean ethnicity) and insight (poor/low/impaired insight/present anosognosia vs. good/high/preserved insight/no anosognosia. We did, however, exclude data from the meta-synthesis if age or education was reported as a categorical variable. Where more than one insight measure was eligible for inclusion, the results were pooled so that the assumption of independence was maintained.

See here for the associated formulas (Borenstein, Hedges, Higgins, & Rothstein, 2009).

**Statistic**	**Conversion One**	**Final Conversion**
Independent (t)	Cohen (d)/Pearson (r)	Fisher (z)/Cohen (d)
Pearson (r)	N/A	Fisher (z) /Cohen (d)
Cohen (d)	Cohen (d)/Pearson (r)	Fisher (z) /Cohen (d)
Spearman (rho)	N/A	Fisher (z) /Cohen (d)
Mann Whitney (U)	Rank Biserial Correlation	Fisher (z) /Cohen (d)

Table A.7. Conversion metrics for effect sizes included in the meta analyses.

3. SENSITIVITY ANALYSES FOR EFFECTS

3.1 Forest Plots

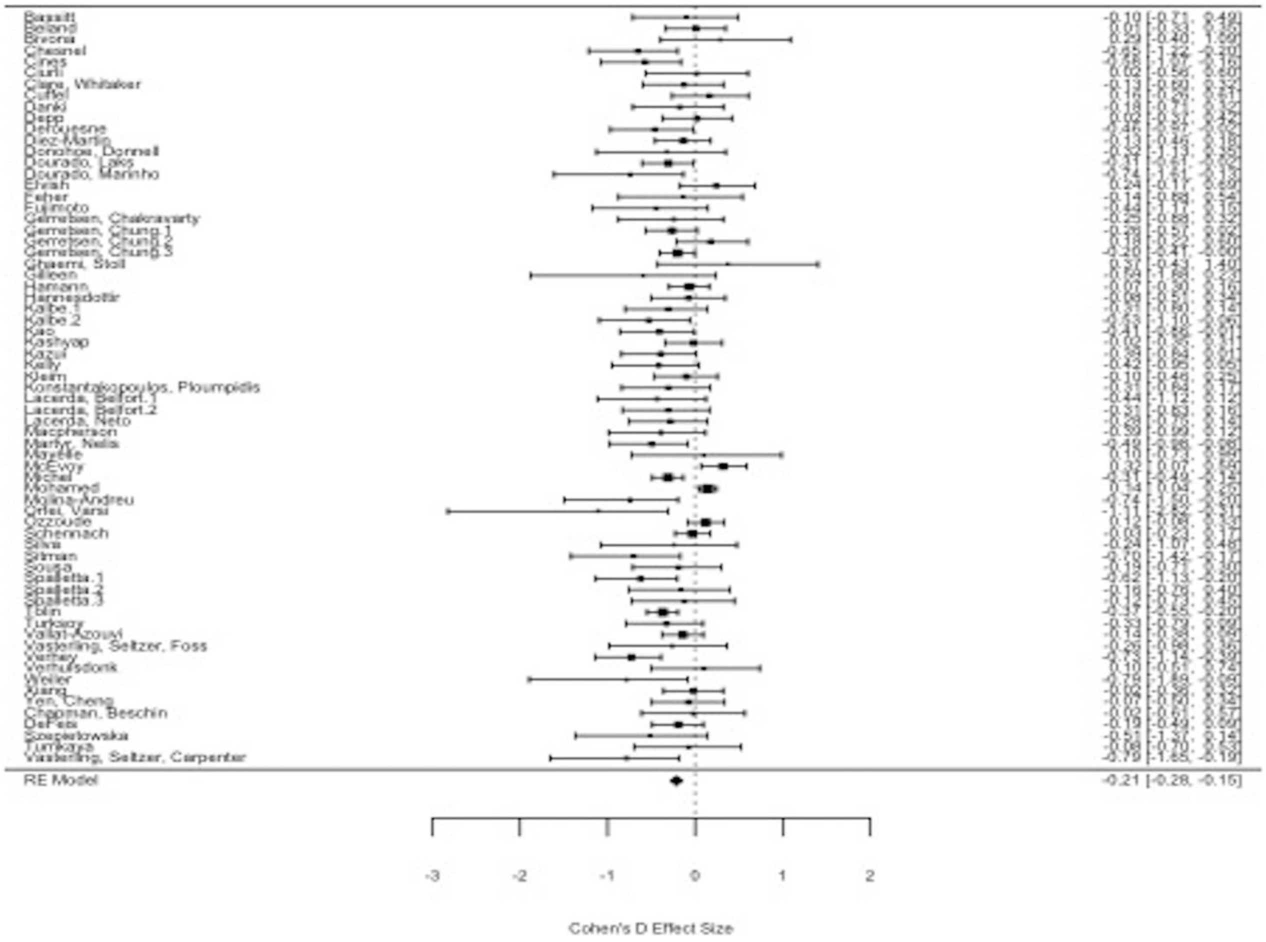

Figure A.1. Forest plot detailing the relationship between insight and age when insight was measured as a continuous variable.

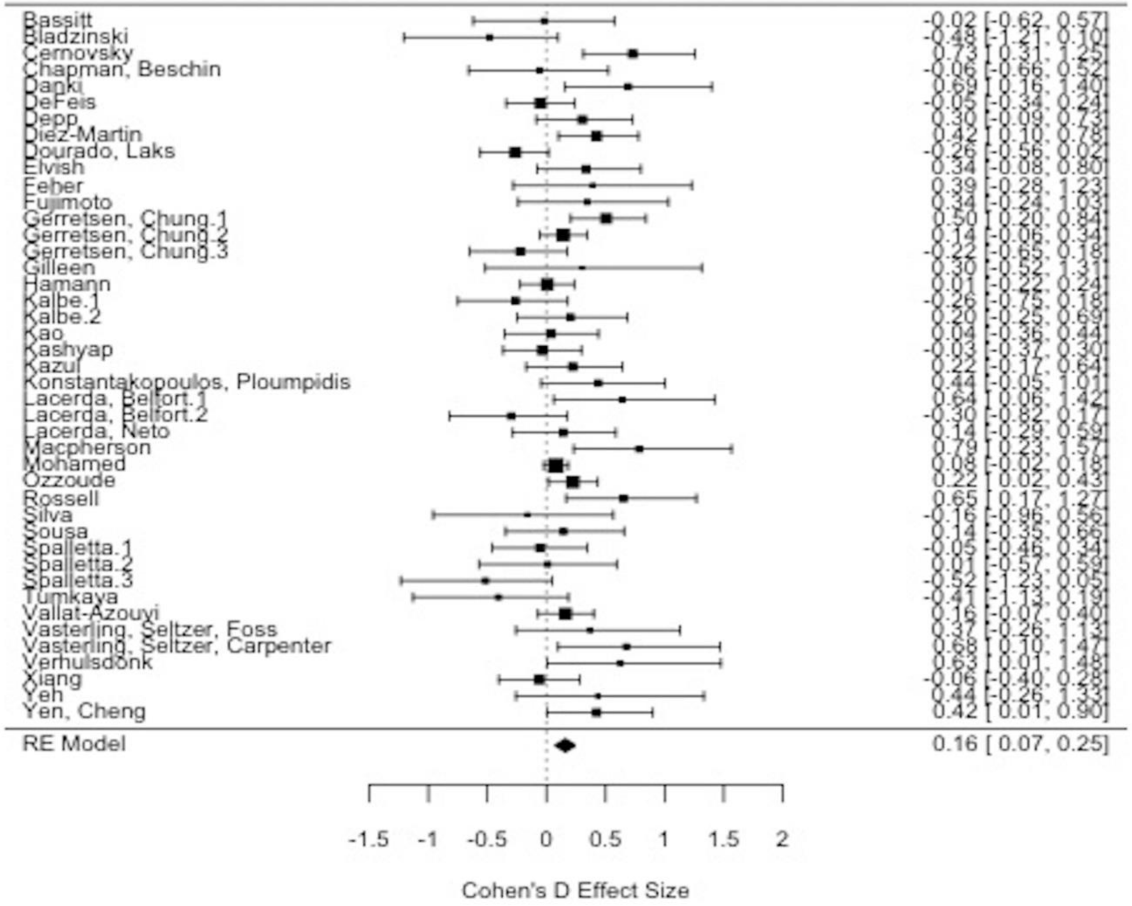

Figure A.2. Forest plot detailing the relationship between insight and education when insight was measured as a continuous variable.

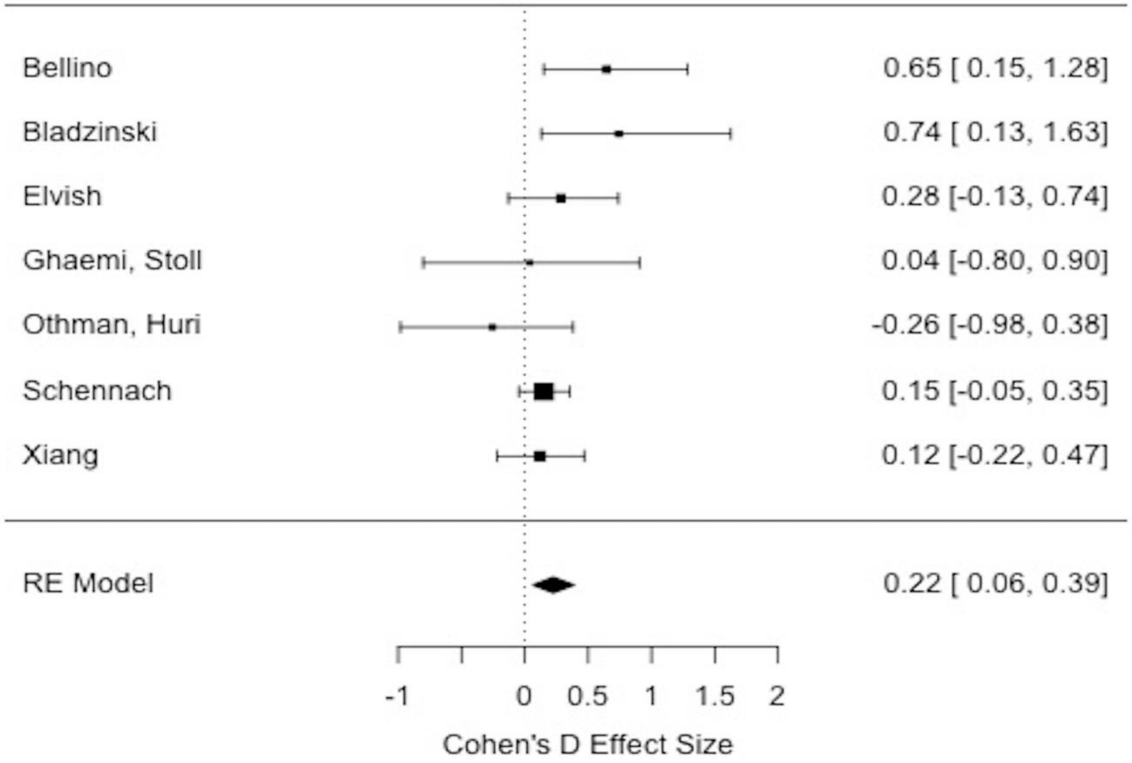

Figure A.3. Forest plot detailing the relationship between insight and employment when insight was measured as a continuous variable.

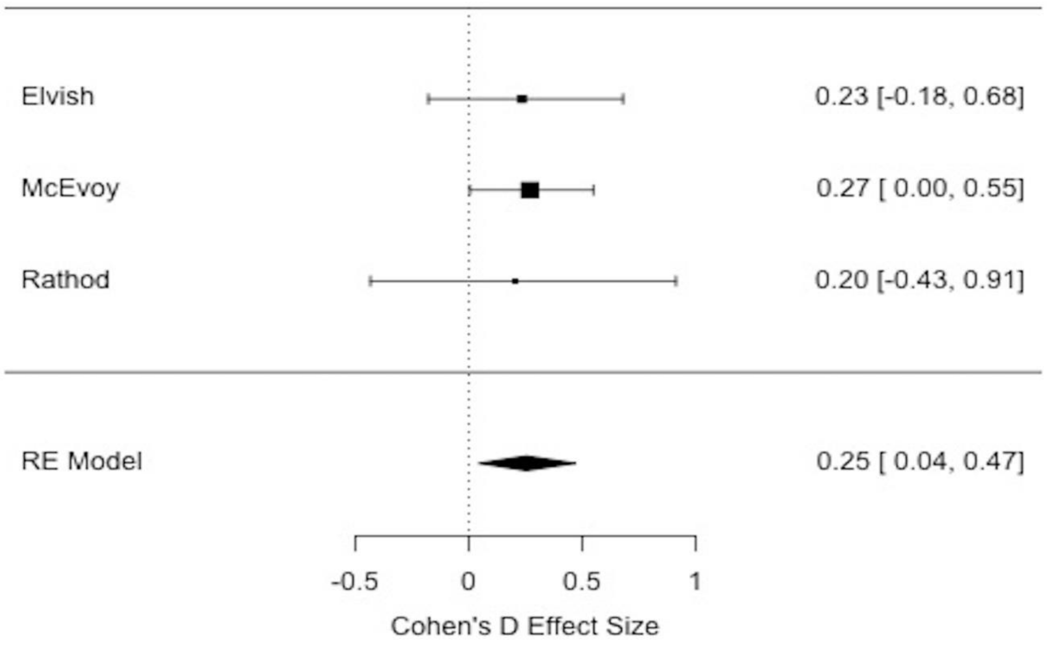

Figure A.4. Forest plot detailing the relationship between insight and ethnicity when insight was measured as a continuous variable (white ethnicity is positive, non-white ethnicity is negative).

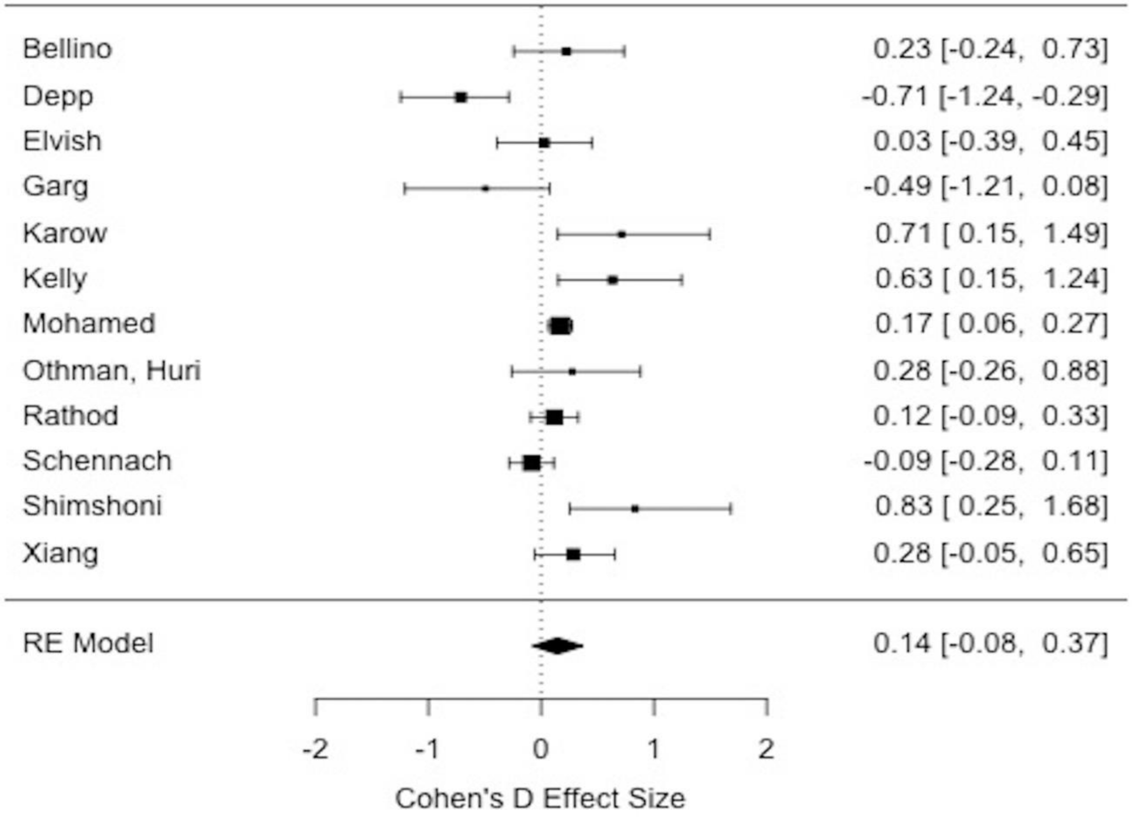

Figure A.5. Forest plot detailing the relationship between insight marital status when insight was measured as a continuous variable (married is positive).

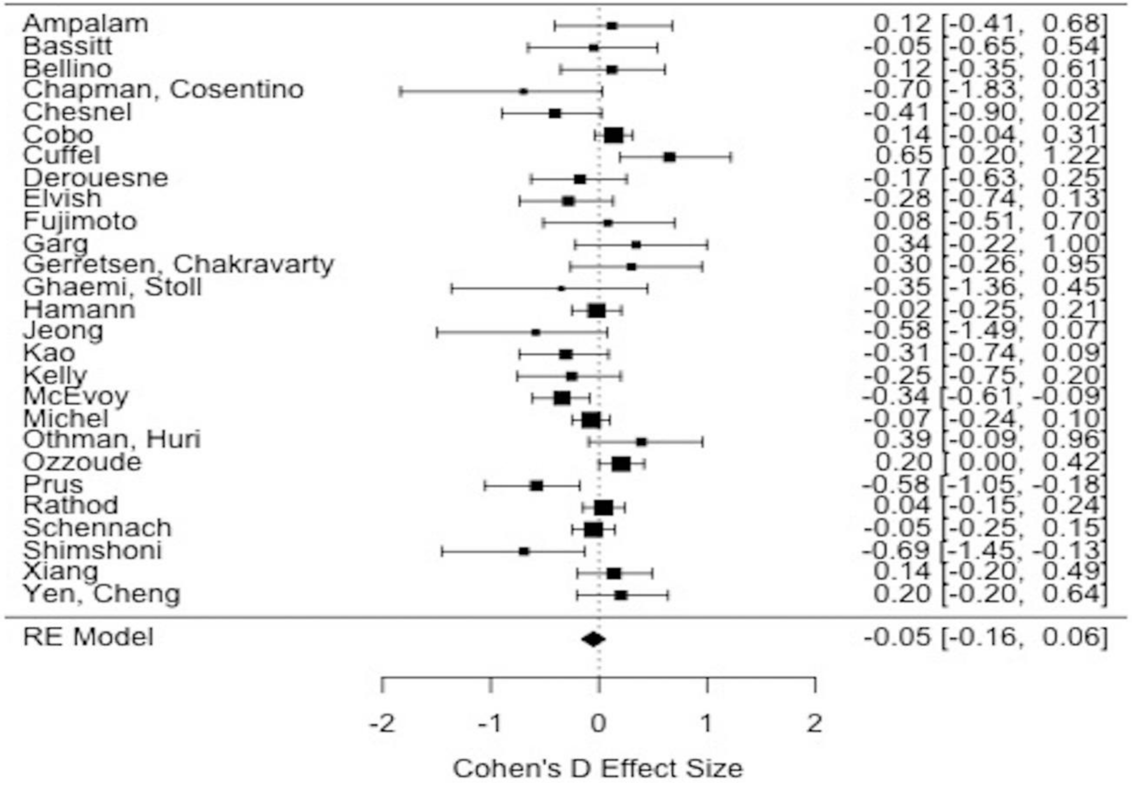

Figure A.6. Forest plot detailing the relationship between insight and sex when insight was measured as a continuous variable.

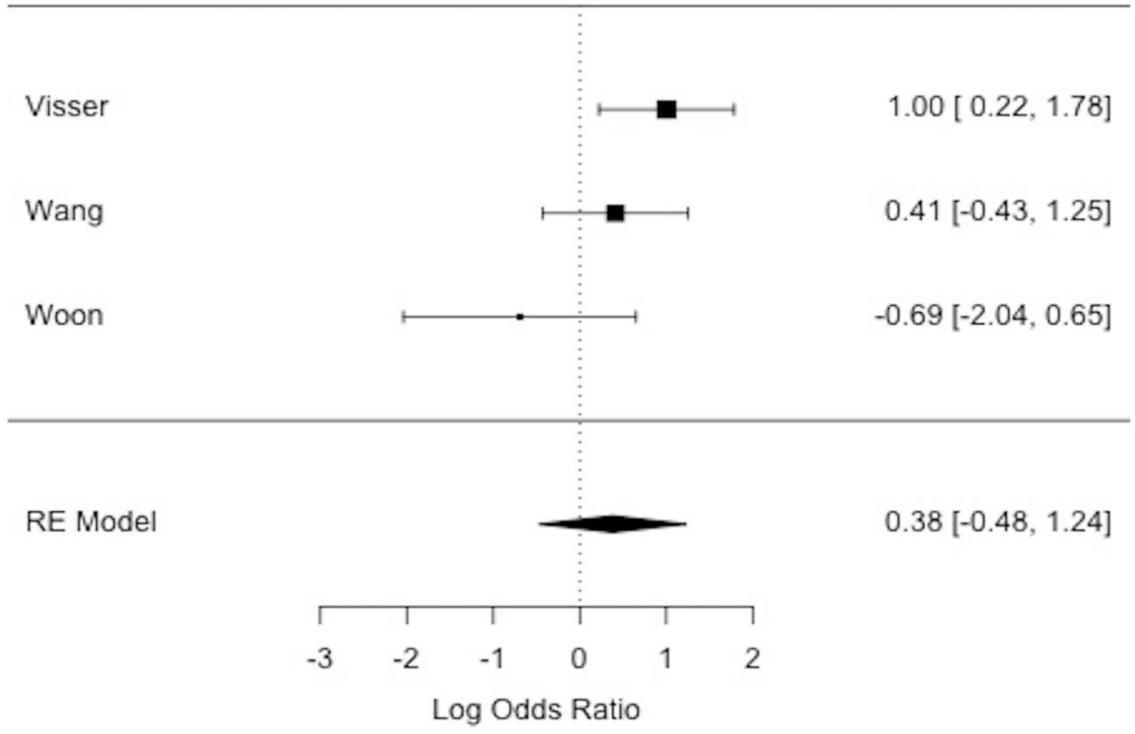

Figure A.7. Forest plot detailing the odds of an employed person being judged to have good insight, relative to an unemployed person.

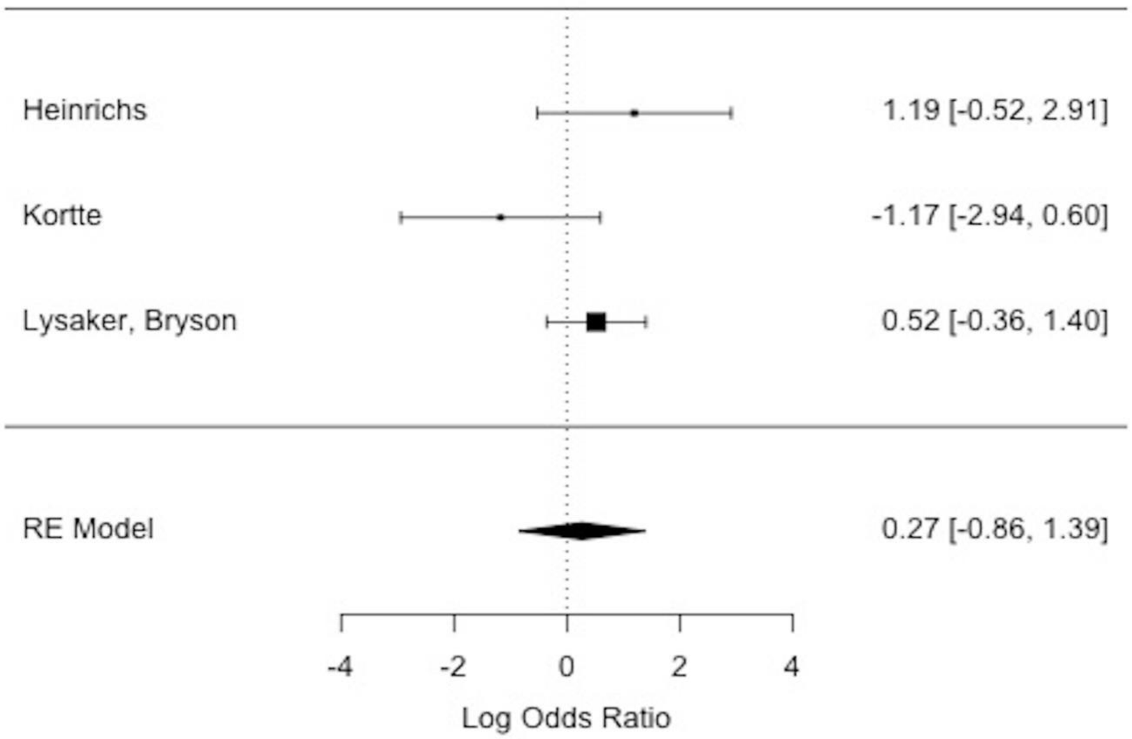

Figure A.8. Forest plot detailing the odds of a white person being judged to have good insight, relative to a black person.

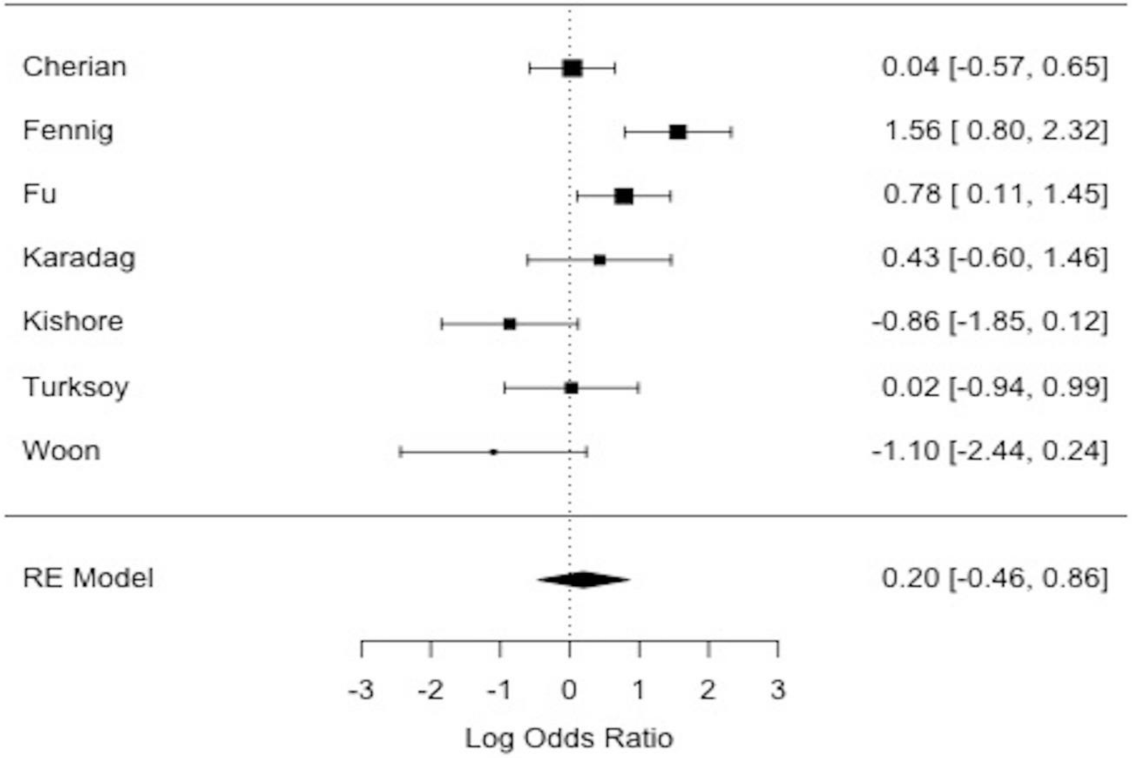

Figure A.9. Forest plot detailing the odds of a married person being judged to have good insight, relative to an unmarried person.

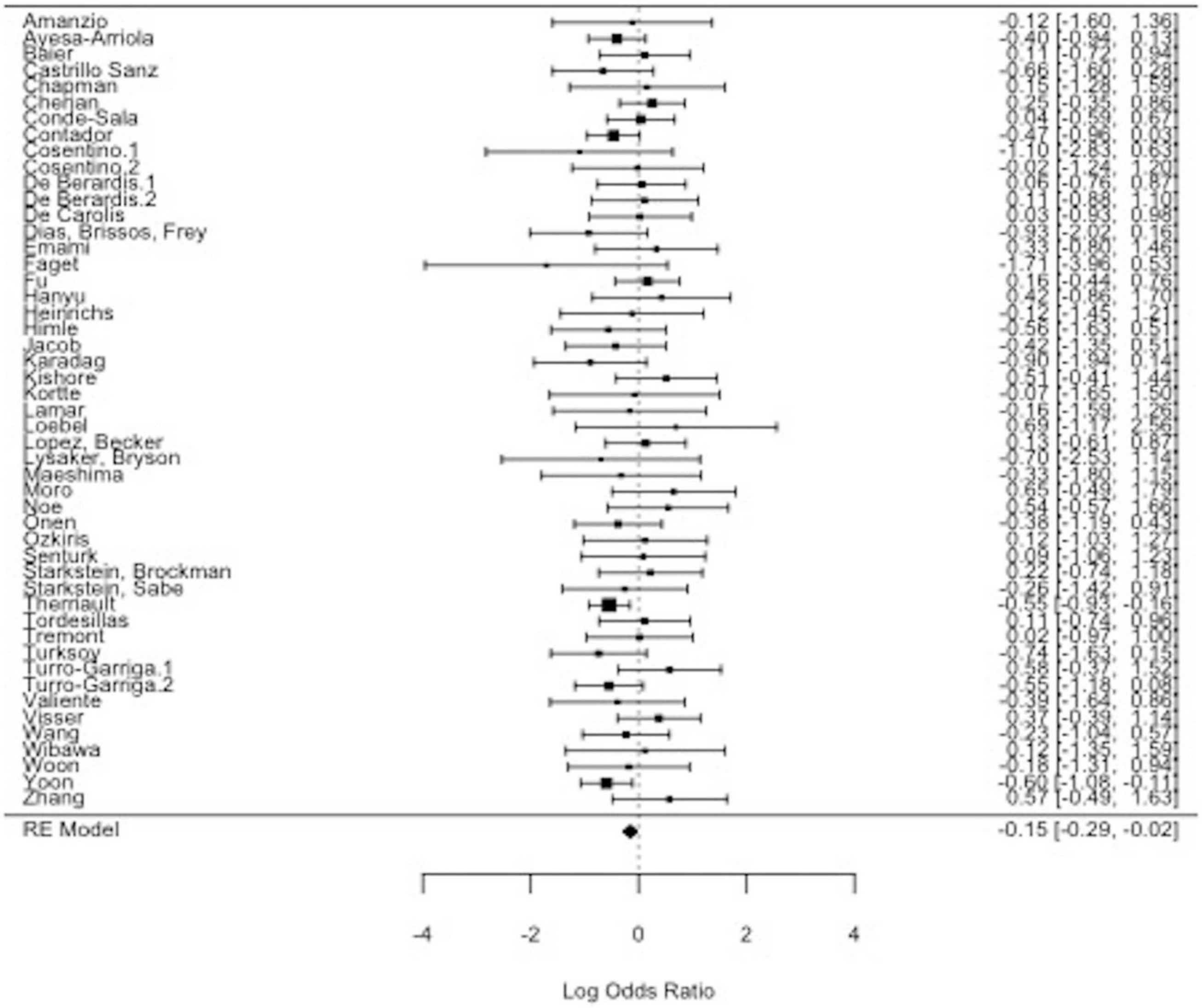
Amanzio -0.12 [-1.60, 1.36]
Ayesa-Arriola -0.40 [-0.94, 0.13]
Baier 0.11 [-0.72, 0.94]
Castrillo Sanz -0.66 [-1.60, 0.28]
Chapman 0.15 [-1.28, 1.59]
Cherian 0.25 [-0.35, 0.86]
Conde-Sala 0.04 [-0.59, 0.67]
Contador -0.47 [-0.96, 0.03]
Cosentino.1 -1.10 [-2.83, 0.63]
Cosentino.2 -0.02 [-1.24, 1.20]
De Berardis.1 0.06 [-0.76, 0.87]
De Berardis.2 0.11 [-0.88, 1.10]
De Carolis 0.03 [-0.93, 0.98]
Dias, Brissos, Frey -0.93 [-2.02, 0.16]
Emami 0.33 [-0.80, 1.46]
Faget -1.71 [-3.96, 0.53]
Fu 0.16 [-0.44, 0.76]
Hanyu 0.42 [-0.86, 1.70]
Heinrichs -0.12 [-1.45, 1.21]
Himle -0.56 [-1.63, 0.51]
Jacob -0.42 [-1.35, 0.51]
Karadag -0.90 [-1.94, 0.14]
Kishore 0.51 [-0.41, 1.44]
Kortte -0.07 [-1.65, 1.50]
Lamar -0.16 [-1.59, 1.26]
Loebel 0.69 [-1.17, 2.56]
Lopez, Becker 0.13 [-0.61, 0.87]
Lysaker, Bryson -0.70 [-2.53, 1.14]
Maeshima -0.33 [-1.80, 1.15]
Moro 0.65 [-0.49, 1.79]
Noe 0.54 [-0.57, 1.66]
Onen -0.38 [-1.19, 0.43]
Ozkiris 0.12 [-1.03, 1.27]
Senturk 0.09 [-1.06, 1.23]
Starkstein, Brockman 0.22 [-0.74, 1.18]
Starkstein, Sabe -0.26 [-1.42, 0.91]
Therriault -0.55 [-0.93, -0.16]
Tordesillas 0.11 [-0.74, 0.96]
Tremont 0.02 [-0.97, 1.00]
Turksoy -0.74 [-1.63, 0.15]
Turro-Garriga.1 0.58 [-0.37, 1.52]
Turro-Garriga.2 -0.55 [-1.18, 0.08]
Valiente -0.39 [-1.64, 0.86]
Visser 0.37 [-0.39, 1.14]
Wang -0.23 [-1.02, 0.57]
Wibawa 0.12 [-1.35, 1.59]
Woon -0.18 [-1.31, 0.94]
Yoon -0.60 [-1.08, -0.11]
Zhang 0.57 [-0.49, 1.63]
RE Model -0.15 [-0.29, -0.02]
-4
-2
0
2
4
Log Odds Ratio

Figure A.10. Forest plot detailing the odds of females being judged to have good insight, relative to a males (males coded as positive).

3.2 Funnel Plots

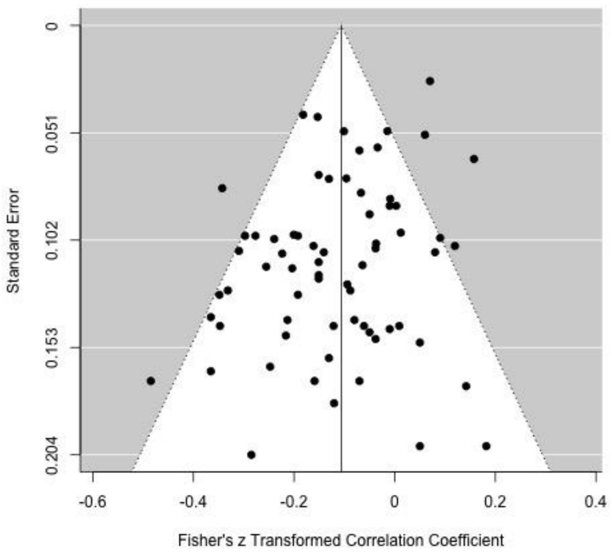

Figure A.11. Funnel plot of studies that investigated the relationship between insight and age when insight was measured as a continuous variable.

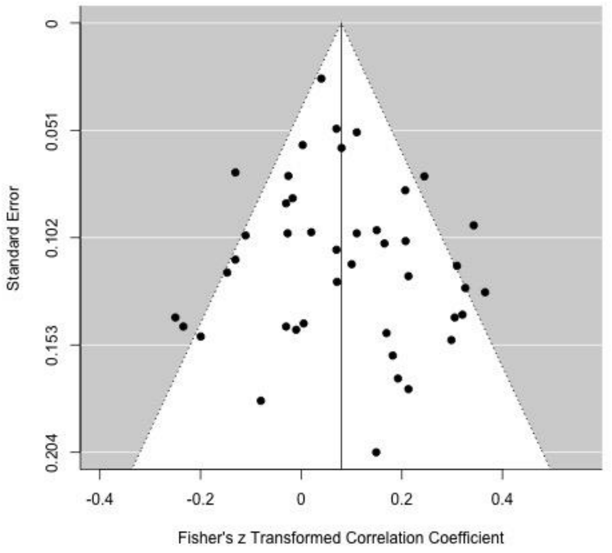

Figure A.12. Funnel plot of studies that investigated the relationship between insight and education when insight was measured as a continuous variable.

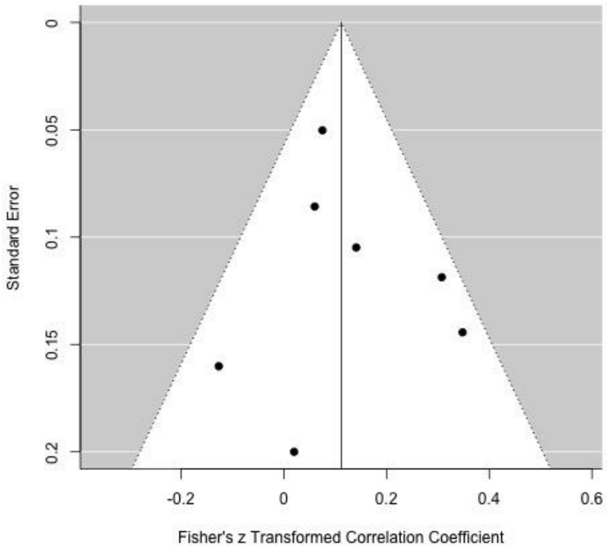

Figure A.13. Funnel plot of studies that investigated the relationship between insight and employment when insight was measured as a continuous variable.

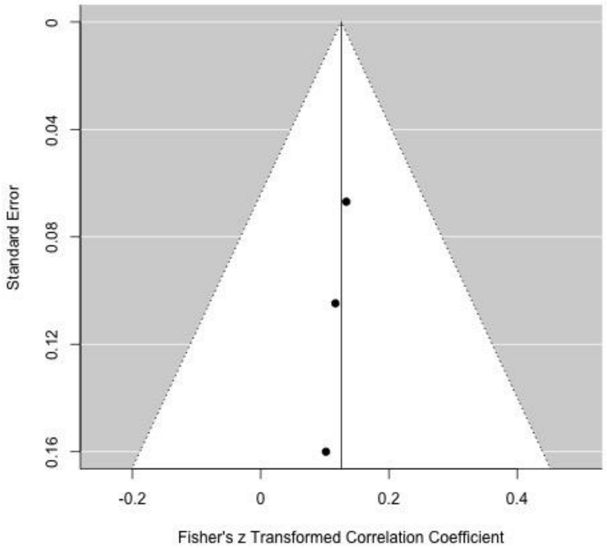

Figure A.14. Funnel plot of studies that investigated the relationship between insight and ethnicity when insight was measured as a continuous variable.

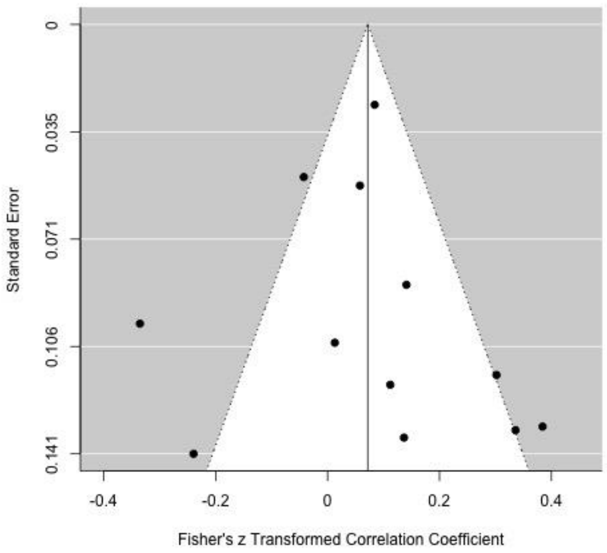

Figure A.15. Funnel plot of studies that investigated the relationship between insight and marital status when insight was measured as a continuous variable.

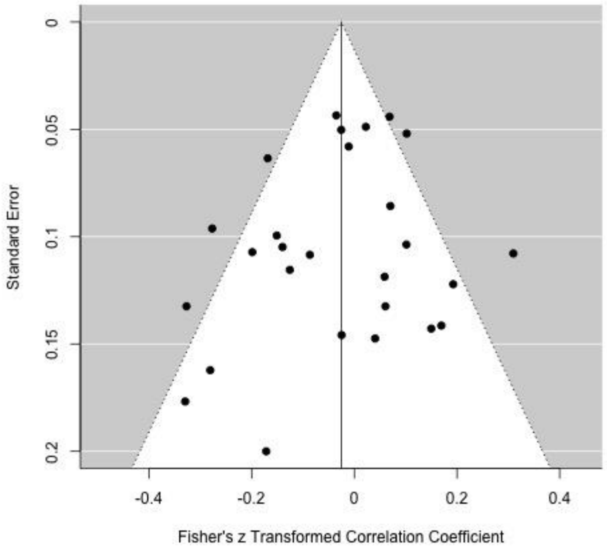

Figure A.16. Funnel plot of studies that investigated the relationship between insight and sex when insight was measured as a continuous variable.

3.3 Cook's Distance Plots

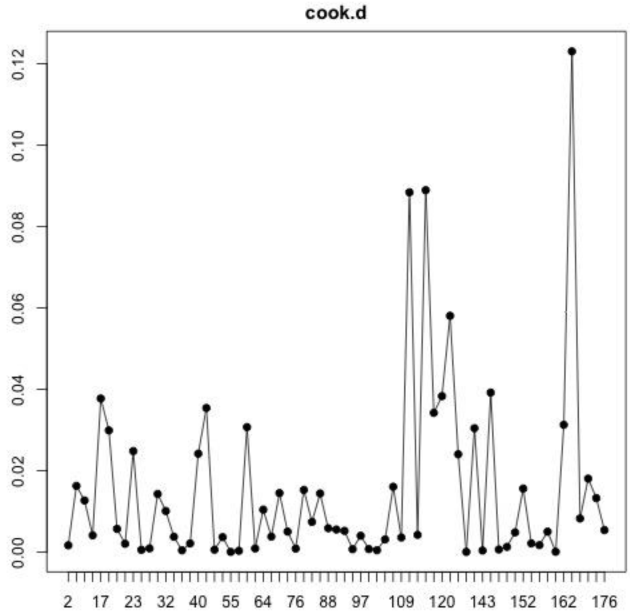

Figure A.17. Cook's distance analysis investigating outliers from studies the relationship between insight and age when insight was measured as a continuous variable.

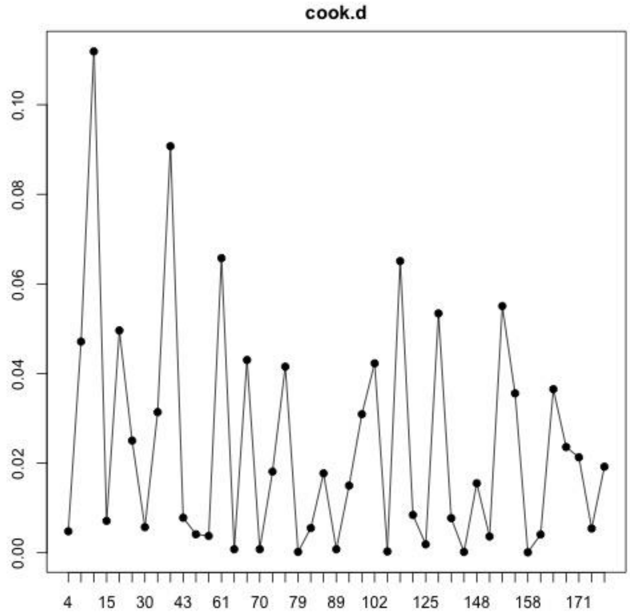

Figure A.18. Cook's distance analysis investigating outliers from studies the relationship between insight and education when insight was measured as a continuous variable.

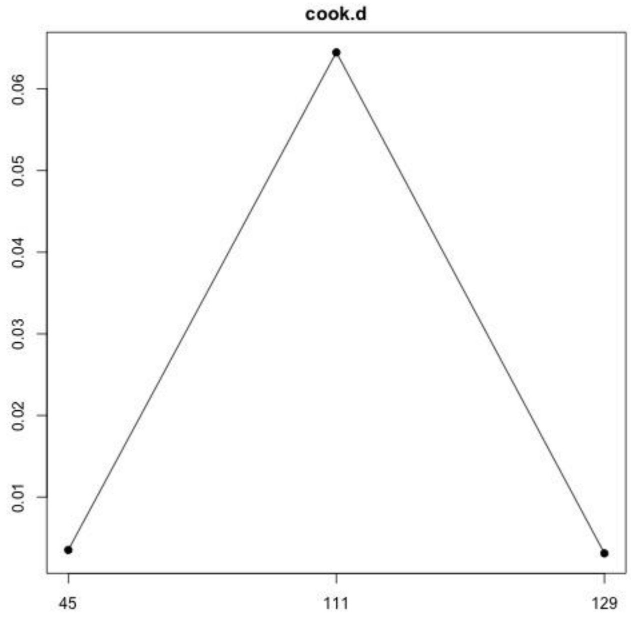

Figure A.19. Cook's distance analysis investigating outliers from studies the relationship between insight and ethnicity when insight was measured as a continuous variable.

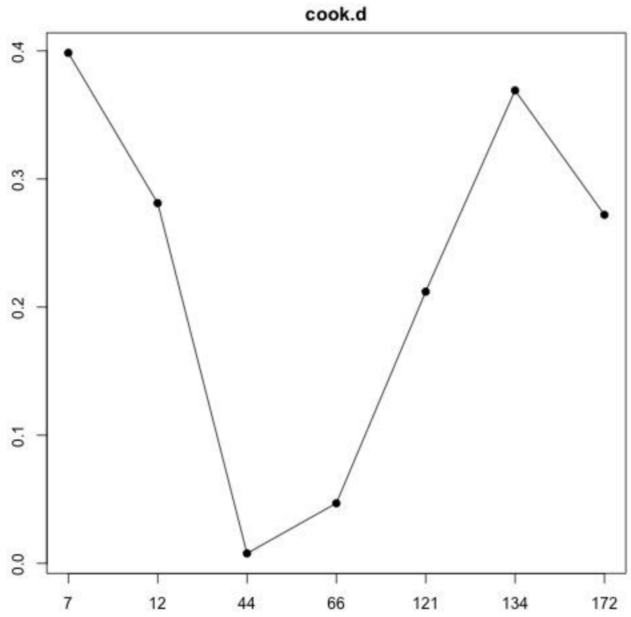

Figure A.20. Cook's distance analysis investigating outliers from studies the relationship between insight and employment when insight was measured as a continuous variable.

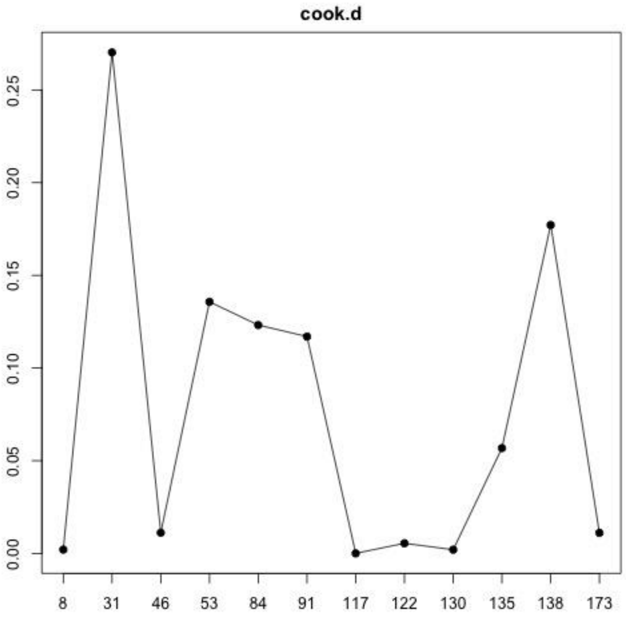

Figure A.21. Cook's distance analysis investigating outliers from studies the relationship between insight and marital status when insight was measured as a continuous variable.

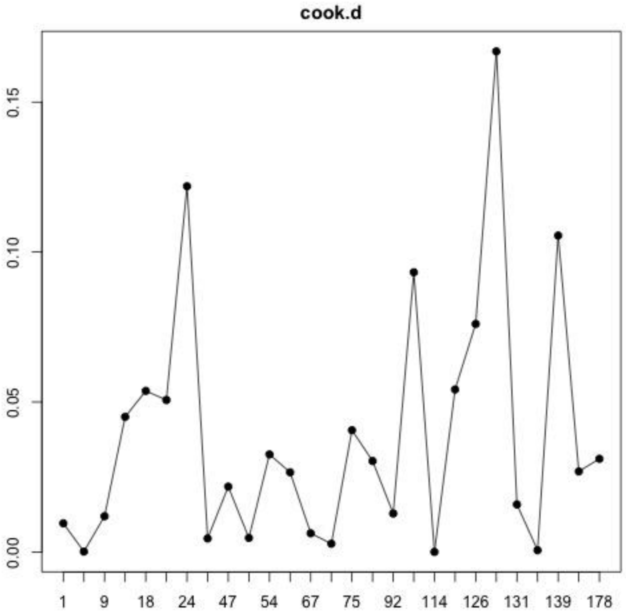

Figure A.22. Cook's distance analysis investigating outliers from studies the relationship between insight and sex when insight was measured as a continuous variable.

4. SUPPLEMENTARY SUBGROUP ANALYSES

4.1 Age effect on insight in Psychiatric and Neurological samples

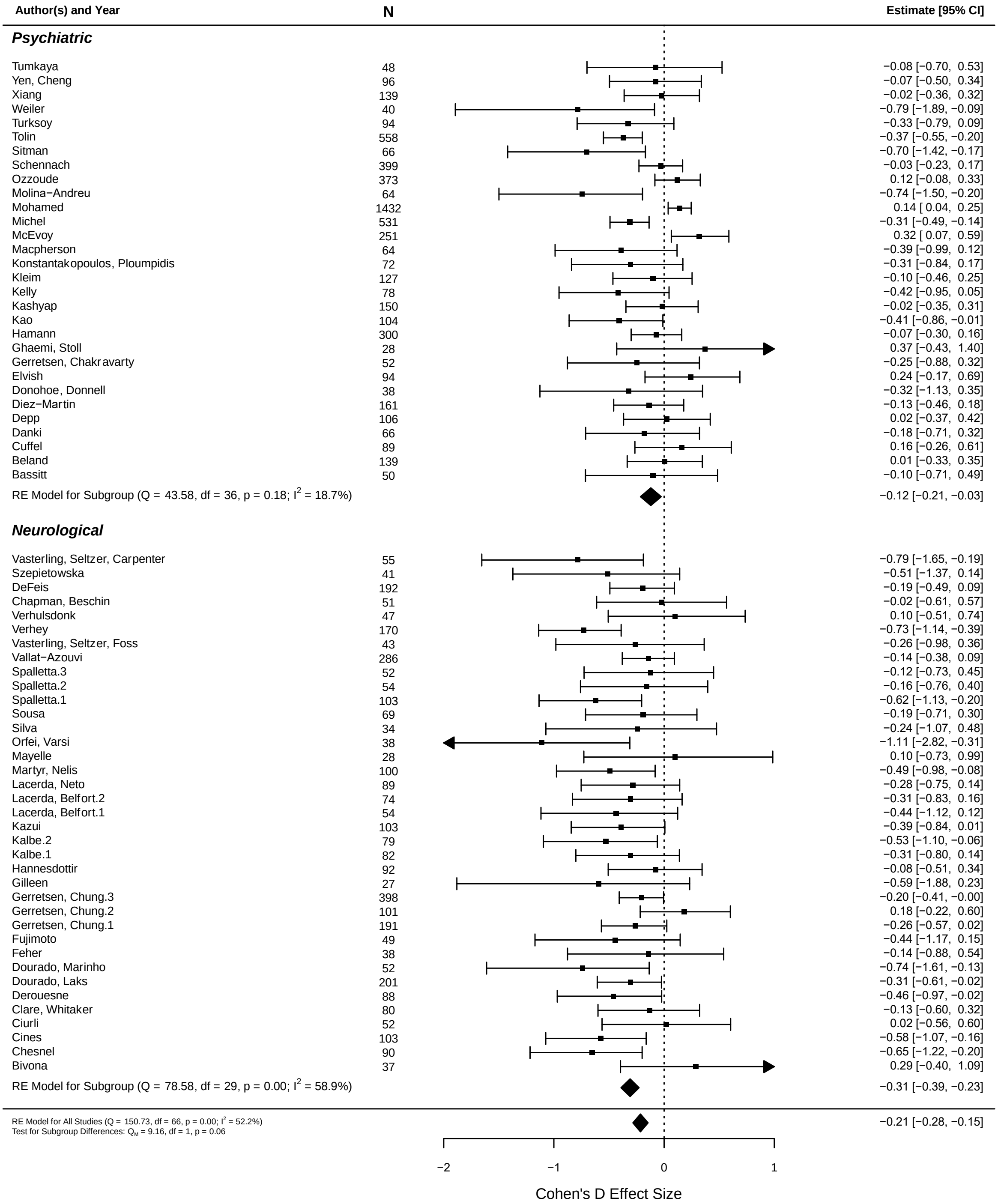

Figure A.23. The effect of age on insight between psychiatric and neurological sub-samples.

4.2 Age effect on insight between diagnoses

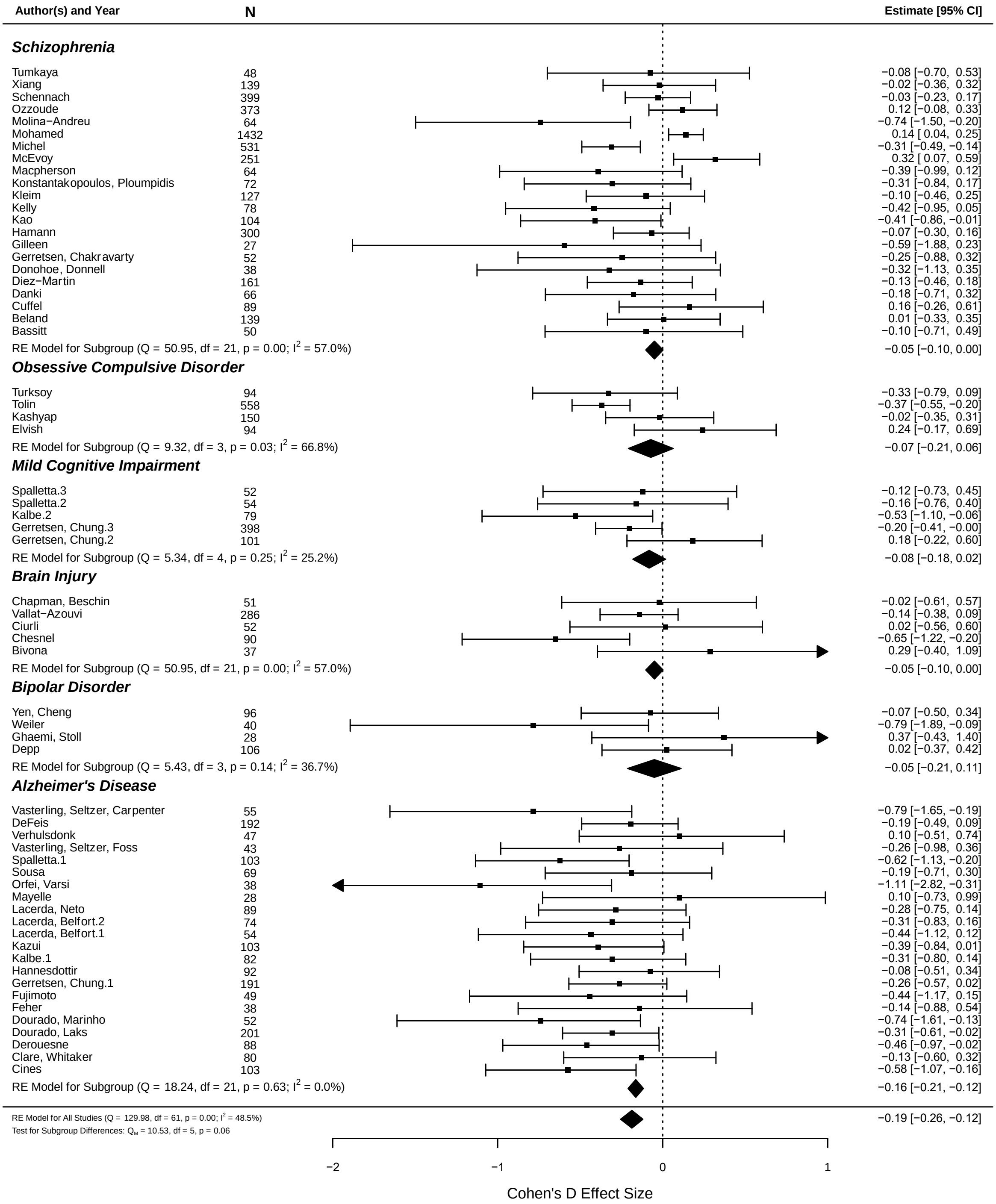

Figure A.24. The effect of age on insight, between different diagnoses.

4.3 Age effect on insight for different types of insight scales

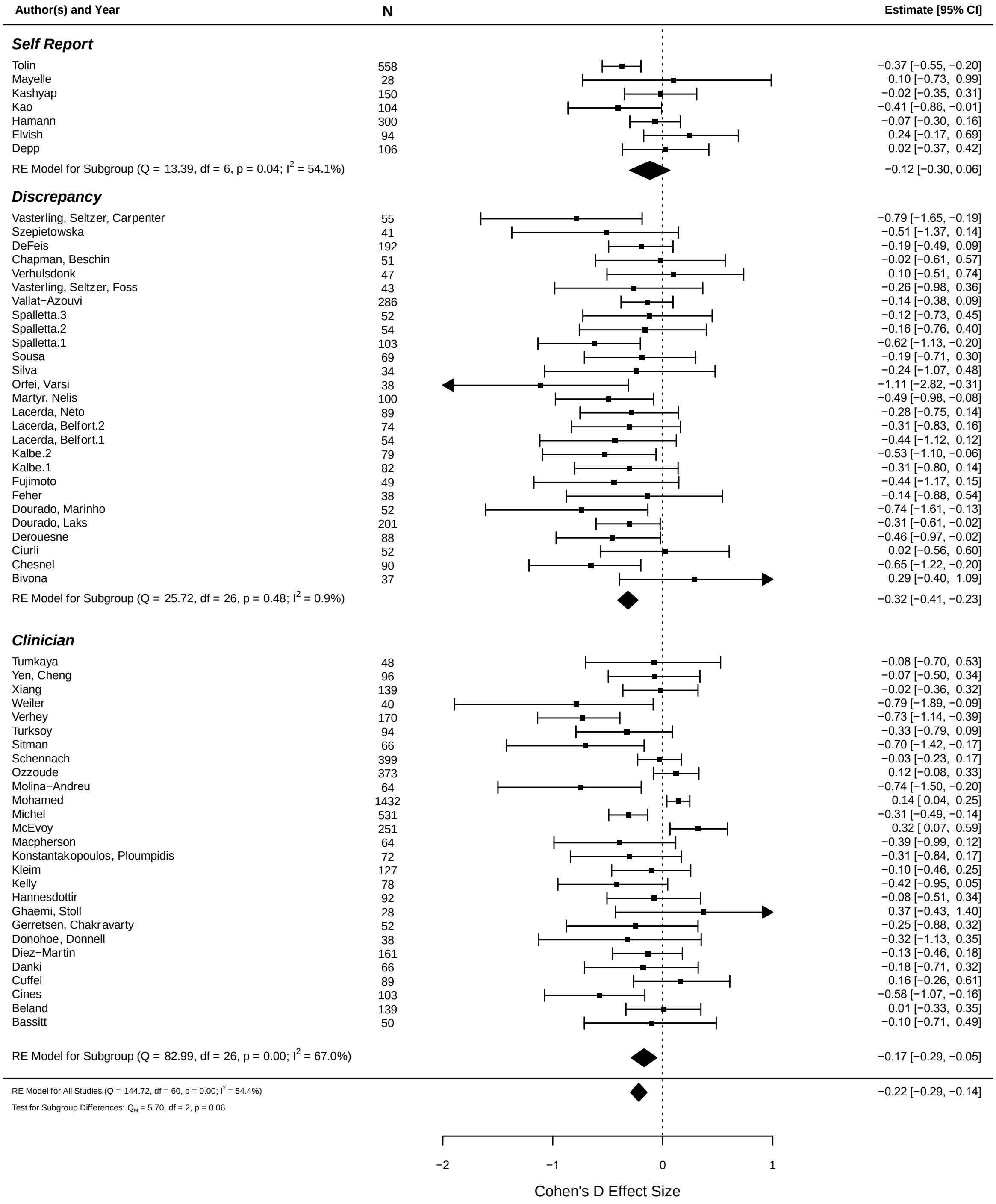

Figure A.23. The effect of age on insight between different types of measurement scales.

5. SUPPLEMENTARY DATA FOR QUALITATIVE SYNTHESIS

5.1 Summary of Longitudinal Results

First Author	Year	Sociodemographic factors	Diagnostic group	Follow up period	Follow ups	Significant results (insight total scores only).
Aalten	2006	Age, education, SES, sex	Dementia	18 months	3	Only better education predicted good insight at 6 months. Younger age, better education, male gender and higher socioeconomic status predicted good insight at 12 months. Better education and higher socioeconomic status predicted good insight at 16 months.
Chan	2014	Age, education	Schizophrenia	1 year	2	Neither variable significantly predicted insight.
Chen	2001	Age, education	SMI inpatients	Variable	1	No variable predicted change in insight.
Comacchio	2020	Gender	Psychosis	5 years	3	Female patients scored worse than males at baseline but improved more than males over time.
Derouesne	1999	Age, education, sex	Alzheimer's disease	Variable (M= 21 months)	1	Only younger age predicted good insight at follow up.
Fennig	1996	Marital status	Psychosis	6 months	1	Being married predicted good insight.
Gerretsen	2017	Age, education	Schizophrenia	28 weeks	1	No variable predicted good insight at follow up.
Greenfeld	1990	BMI	Anorexia	Variable (1.2 to 10 years)		Increase in BMI between hospitalization and follow-up predicted better insight.
Hanseeuw	2020	Age	Alzheimer's disease	Var (M= 3 years)	3.3	Older patients had faster decreases in awareness over time, than did younger patients.
Lysaker	1995	Age, education, employment	Schizophrenia	26 weeks	1	No variable predicted change in insight.
Ozzoude	2019	Age, education, sex	Schizophrenia	Variable (1-3 months)	1	Only male gender predicted good insight at follow up.
Rathod	2005	Age, employment, ethnicity, marital status, sex	Schizophrenia	12 months	2	Younger age, being in part time employment and white ethnicity predicted good insight at 5 months. Only white ethnicity predicted good insight at 12 months.
Sanchez-Torres	2015	Age, , education, sex	Schizophrenia	10 years	1	Only female gender predicted good insight at follow up.
Schennach	2012	Age, employment, marital status, sex	Schizophrenia	1 year	1	Only being employed predicted improvement in insight.
Setkowski	2016	Age, country, marital status, sex	SMI inpatients	2 years	1	No variable predicted change in insight.
Silva	2016	Age, education, sex	MCI	2 years	1	No variable predicted good insight at follow up.
Sousa	2015	Age, education, carer burden sex	Alzheimer's disease	12 months	1	No variable predicted good insight at follow up.
Van Vliet	2013	Sex	Alzheimer's disease	1 year	2	Male gender predicted good insight over the follow up period.
Vasterling	1997	Age, , education	Alzheimer's disease	Variable (M= 16.4 months)	1	No variable predicted change in insight.
Wiffen	2010	Sex	Schizophrenia	6 months	3	No variable predicted good insight at follow up.
Yen	2003	Age, education	Bipolar disorder	Variable (M= 32.8 days)	1	No variable predicted change in insight.

Table A.11. Sociodemographic associations with insight, when measured as a longitudinal variable.

6. REVIEW PROTOCOL

6.1 Insight Review Protocol (Amended)

**Included**	**Excluded**
**Design**	
Cross-sectional	Qualitative only studies
Epidemiological analyses	Non-systematic review
Case note analyses	Non-English language
Case control	Commentaries
Retrospective cohort	Case studies
Prospective cohort	Small N samples (<20 eligible participants)
Randomised controlled studies	Conference abstracts
Systematic review	Brief reports
Meta-analyses	Books
Unpublished dissertations of the above	
**Population**	
Cognitive impairment	Non-human participants
Dementia/stroke	Palliative care
Mental health diagnosis	Rare neuropsychiatric syndromes
Substance use disorder	Intellectual/Learning disability
All ages	Healthy populations
	Physical health problems only
**Focus**	
Clinical insight	Mental capacity focus only
Anosognosia	Non-clinical or non-cognitive focus
Neuroimaging of relevant populations (other eligibility criteria applies)	Methodological focus only
Neuropsychological testing of relevant populations (other eligibility criteria applies)	Interventions for medical professionals<br>Cognitive Insight
Metacognitive experiments of relevant populations (other eligibility criteria applies)	
**Data/Outcomes**	
Quantitative measure of insight via:<br>• Full validated insight assessment tool (and international adaptations)<br>• Subjective clinical judgment<br>• Awareness discrepancy measure	No quantitative measure of insight or measure can't be linked to a protected characteristic
PANSS G12/OVIS item 11/Y-BOCS item 11 are the only eligible single item scales	No recorded protected characteristic (or insufficient data to merit a conclusion)
Self-reports vs. neuropsychological test discrepancies	Insight subscales or single item scales (except PANSS G12/OVIS item 11/Y-BOCS item 11)<br>Protected characteristic included as a covariate only

7. REVIEW SEARCH TERMS

7.1 MEDLINE

(("clinical insight"[All Fields] OR "cognitive insight"[All Fields] OR "anosognosia"[All Fields] OR ("insight"[All Fields] AND "self awareness"[All Fields]) OR ("insight"[All Fields] AND "awareness of deficit"[All Fields]) OR ("insight"[All Fields] AND "self certainty"[All Fields]) OR "patient's insight"[All Fields] OR "patient insight"[All Fields] OR "lack of insight"[All Fields] OR "loss of insight"[All Fields] OR "impaired insight"[All Fields] OR "poor insight"[All Fields] OR "lack of insight"[All Fields])) AND ((("age" OR "children" OR "younger" OR "older" OR "gender*" OR "sex" OR "male*" OR "female*" OR "rac*" OR "ethnic*" OR "minority" OR "Afro*" OR "African" OR "Caribbean" OR "Asian" OR "Indian" OR "Chinese" OR "sexual orient*" OR "homosexual" OR "bisexual" OR "trans*" OR "gender AND reassign*" OR "disab*" OR "plegic" OR "special AND needs" OR "religi*" OR "spiritu*" OR "pregnan*" OR "matern*" OR "natal")))

7.2 Cochrane Database for Systematic Reviews & Cochrane Controlled Register of Trials (CENTRAL)

#1 "clinical insight" OR "cognitive insight" OR "anosognosia"
#2 ("insight" AND "self awareness") OR ("insight" AND "awareness of deficit") OR ("insight" AND "self certainty")
#3 "patient's insight" OR "patient insight" OR "lack of insight" OR "loss of insight" OR "impaired insight" OR "poor insight" OR "lack of insight"
#4 "Beck Cognitive Insight Scale" OR "Birchwood Insight Scale" OR "Scale to assess unawareness of mental disorder" OR "Positive negative syndrome scale" OR "schedule for the assessment of insight" OR "self reflection insight scale" OR "self appraisal of illness questionnaire" OR "mood disorders insight scale"
#5 ("BCIS" AND "insight"):ti,ab,kw OR ("BIS" AND "insight"):ti,ab,kw OR ("SUMD" AND "insight"):ti,ab,kw OR ("PANSS" AND "insight"):ti,ab,kw OR ("SAI" AND "insight"):ti,ab,kw OR ("SRIS" AND "insight"):ti,ab,kw OR ("SAIQ" AND "insight"):ti,ab,kw OR ("MDIS" AND "insight"):ti,ab,kw
#6 "age" OR "children" OR "younger" OR "older" OR "gender*" OR "sex" OR "male*" OR "female*" OR "rac*" OR "ethnic*" OR "minority" OR "Afro*" OR "African" OR "Caribbean" OR "Asian" OR "Indian" OR "Chinese" OR "sexual orient*" OR "homosexual" OR "bisexual" OR "trans*" OR "gender AND reassign*" OR "disab*" OR "plegic" OR "special AND needs" OR "religi*" OR "spiritu*" OR "pregnan*" OR "matern*" OR "natal"
#7 (#1 or #2 or #3 or #4 or #5) = 257 (51 reviews, 206 trials.
#8 (#7 and #6) = 190 (51 reviews, 139 trials).

7.3 Web of Science

Indexes = SCI-EXPANDED, SSCI, CPCI-S, CPCI-SHH, ESCI. Languages = English, Document Type = Article, Timespan = All years (1900-2019)
#1 ALL= ("clinical insight" OR "cognitive insight" OR "anosognosia”)
#2 ALL= ("insight" AND "self awareness") OR ("insight" AND "awareness of deficit") OR ("insight" AND "self certainty")
#3 ALL= ("patient's insight" OR "patient insight" OR "lack of insight" OR "loss of insight" OR "impaired insight" OR "poor insight" OR "lack of insight")
#4 ALL= ("Beck Cognitive Insight Scale" OR "Birchwood Insight Scale" OR "Scale to assess unawareness of mental disorder" OR "Positive negative syndrome scale")
#5 ALL= ("schedule for the assessment of insight" OR "self reflection insight scale" OR "self appraisal of illness questionnaire" OR "mood disorders insight scale")
#6 TS= (("BCIS" NEAR/5 "insight") OR ("BIS" NEAR/5 "insight") OR ("SUMD" NEAR/5 "insight") OR ("PANSS" NEAR/5 "insight") OR ("SAI" NEAR/5 "insight") OR ("SRIS" NEAR/5 "insight") OR ("SAIQ" NEAR/5 "insight") OR ("MDIS" NEAR/5 "insight"))
#7 ALL= ("age" OR "children" OR "younger" OR "older" OR "gender*" OR "sex" OR "male*" OR "female*" OR "rac*" OR "ethnic*" OR "minority" OR "Afro*" OR "African" OR "Caribbean" OR "Asian" OR "Indian" OR "Chinese" OR "sexual orient*" OR "homosexual" OR "bisexual" OR "trans*" OR "gender AND reassign*" OR "disab*" OR "plegic" OR "special AND needs" OR "religi*" OR "spiritu*" OR "pregnan*" OR "matern*" OR "natal")
#8 ALL= ((#1 OR #2 OR #3 OR #4 OR #5 OR #6) AND #7) = 1043 Articles.

7.4 EMBASE (& EMBASE Classic)

Dates: 1947-2019, Limits: Human participants only, English language
#1 Text Word: "clinical insight" or "cognitive insight" or "anosognosia" or ("insight" and "self awareness") or ("insight" and "awareness of deficit") or ("insight" and "self certainty") or "patient's insight" or "patient insight" or "lack of insight" or "loss of insight" or "impaired insight" or "poor insight" or "lack of insight" or "Beck Cognitive Insight Scale" or "Birchwood Insight Scale" or "Scale to assess unawareness of mental disorder" or "Positive negative syndrome scale" or "schedule for the assessment of insight" or "self reflection insight scale" or "self appraisal of illness questionnaire" or "mood disorders insight scale"
#2 Text Word: ("BCIS" adj5 "insight") or ("BIS" adj5 "insight") or ("SUMD" adj5 "insight") or ("PANSS" adj5 "insight") or ("SAI" adj5 "insight") or ("SRIS" adj5 "insight") or ("SAIQ" adj5 "insight") or ("MDIS" adj5 "insight")
#3 Text Word: "age" or "children" or "younger" or "older" or "gender*" or "sex" or "male*" or "female*" or "rac*" or "ethnic*" or "minority" or "Afro*" or "African" or "Caribbean" or "Asian" or "Indian" or "Chinese" or "sexual orient*" or "homosexual" or "bisexual" or "trans*" or "gender AND reassign*" or "disab*" or "plegic" or "special AND needs" or "religi*" or "spiritu*" or "pregnan*" or "matern*" or "natal"
#4 All Fields: (#1 or #2) AND #3 = 1356 records.

7.5 CINAHL

Limits: English language only, Human participants,
#1 TX: "clinical insight" or "cognitive insight" or "anosognosia" or ("insight" and "self awareness") or ("insight" and "awareness of deficit") or ("insight" and "self certainty") or "patient's insight" or "patient insight" or "lack of insight" or "loss of insight" or "impaired insight" or "poor insight" or "lack of insight")
#2 TX: "Beck Cognitive Insight Scale" or "Birchwood Insight Scale" or "Scale to assess unawareness of mental disorder" or "Positive negative syndrome scale" or "schedule for the assessment of insight" or "self reflection insight scale" or "self appraisal of illness questionnaire" or "mood disorders insight scale"
#3 TX: ("BCIS" N5 "insight") or ("BIS" N5 "insight") or ("SUMD" N5 "insight") or ("PANSS" N5 "insight") or ("SAI" N5 "insight") or ("SRIS" N5 "insight") or ("SAIQ" N5 "insight")) or ("MDIS" N5 "insight")
#4 TX: "age" or "children" or "younger" or "older" or "gender*" or "sex" or "male*" or "female*" or "rac*" or "ethnic*" or "minority" or "Afro*" or "African" or "Caribbean" or "Asian" or "Indian" or "Chinese" or "sexual orient*" or "homosexual" or "bisexual" or "trans*" or "gender AND reassign*" or "disab*" or "plegic" or "special AND needs" or "religi*" or "spiritu*" or "pregnan*" or "matern*" or "natal"
#1 TX ("clinical insight" or "cognitive insight" or "anosognosia" or ("insight" and "self awareness") or ("insight" and "awareness of deficit") or ("insight" and "self certainty") or "patient's insight" or "patient insight" or "lack of insight" or "loss of insight" or "impaired insight" or "poor insight" or "lack of insight"))
#2 TX ("Beck Cognitive Insight Scale" or "Birchwood Insight Scale" or "Scale to assess unawareness of mental disorder" or "Positive negative syndrome scale" or "schedule for the assessment of insight" or "self reflection insight scale" or "self appraisal of illness questionnaire" or "mood disorders insight scale")
#3 TX (("BCIS" N5 "insight") or ("BIS" N5 "insight") or ("SUMD" N5 "insight") or ("PANSS" N5 "insight") or ("SAI" N5 "insight") or ("SRIS" N5 "insight") or ("SAIQ" N5 "insight")) or ("MDIS" N5 "insight"))
#4 ("age" or "children" or "younger" or "older" or "gender*" or "sex" or "male*" or "female*" or "rac*" or "ethnic*" or "minority" or "Afro*" or "African" or "Caribbean" or "Asian" or "Indian" or "Chinese" or "sexual orient*" or "homosexual" or "bisexual" or "trans*" or "gender AND reassign*" or "disab*" or "plegic" or "special AND needs" or "religi*" or "spiritu*" or "pregnan*" or "matern*" or "natal")
#5 TX: (#1 or #2 or #3) AND #4 = 943 records.

7.6 PsycINFO

Limits: No date filter (1806-2019), English language, Human participants
#1 Text Word: "clinical insight" or "cognitive insight" or "anosognosia" or ("insight" and "self awareness") or ("insight" and "awareness of deficit") or ("insight" and "self certainty") or "patient's insight" or "patient insight" or "lack of insight" or "loss of insight" or "impaired insight" or "poor insight" or "lack of insight" or "Beck Cognitive Insight Scale" or "Birchwood Insight Scale" or "Scale to assess unawareness of mental disorder" or "Positive negative syndrome scale" or "schedule for the assessment of insight" or "self reflection insight scale" or "self appraisal of illness questionnaire" or "mood disorders insight scale"
#2 Text Word: ("BCIS" w5 "insight") or ("BIS" w5 "insight") or ("SUMD" w5 "insight") or ("PANSS" w5 "insight") or ("SAI" w5 "insight") or ("SRIS" w5 "insight") or ("SAIQ" w5 "insight") or ("MDIS" w5 "insight")

#3 Text Word: "age" or "children" or "younger" or "older" or "gender*" or "sex" or "male*" or "female*" or "rac*" or "ethnic*" or "minority" or "Afro*" or "African" or "Caribbean" or "Asian" or "Indian" or "Chinese" or "sexual orient*" or "homosexual" or "bisexual" or "trans*" or "gender AND reassign*" or "disab*" or "plegic" or "special AND needs" or "religi*" or "spiritu*" or "pregnan*" or "matern*" or "natal"
#4 All Fields: (#1 or #2) AND #3 = 953 records

8. REFERENCES

Meta Analysis

Amanzio, M., Torta, D. M., Sacco, K., Cauda, F., D'Agata, F., Duca, S., . . . Geminiani, G. C. (2011). Unawareness of deficits in Alzheimer's disease: role of the cingulate cortex. *Brain, 134*(Pt 4), 1061-1076. doi:10.1093/brain/awr020

Ampalam, P., Deepthi, R., & Vadaparty, P. (2012). Schizophrenia - insight, depression: a correlation study. *Indian J Psychol Med, 34*(1), 44-48. doi:10.4103/0253-7176.96158

Ayesa-Arriola, R., Moríñigo, J. D. L., David, A. S., Pérez-Iglesias, R., Rodríguez-Sánchez, J. M., & Crespo-Facorro, B. (2014). Lack of insight 3 years after first-episode psychosis: an unchangeable illness trait determined from first presentation? *Schizophrenia research, 157*(1-3), 271-277. doi:10.1016/j.schres.2014.05.011

Baier, B., Karnath, H., Baier, B., & Karnath, H. O. (2005). Incidence and diagnosis of anosognosia for hemiparesis revisited. *Journal of Neurology, Neurosurgery & Psychiatry, 76*(3), 358-361. Retrieved from http://search.ebscohost.com/login.aspx?direct=true&db=cin20&AN=106487280&site=ehost-live
https://jnnp.bmj.com/content/jnnp/76/3/358.full.pdf

Bassitt, D. P., Neto, M. R., de Castro, C. C., & Busatto, G. F. (2007). Insight and regional brain volumes in schizophrenia. *Eur Arch Psychiatry Clin Neurosci, 257*(1), 58-62. doi:10.1007/s00406-006-0685-z

Beland, S., & Lepage, M. (2017). The relative contributions of social cognition and self-reflectiveness to clinical insight in enduring schizophrenia. *Psychiatry Res, 258*, 116-123. doi:10.1016/j.psychres.2017.09.082

Bellino, S., Patria, L., Ziero, S., & Bogetto, F. (2005). Clinical picture of obsessive-compulsive disorder with poor insight: a regression model. *Psychiatry Res, 136*(2-3), 223-231. doi:10.1016/j.psychres.2004.04.015

Bivona, U., Ciurli, P., Barba, C., Onder, G., Azicnuda, E., Silvestro, D., . . . Formisano, R. (2008). Executive function and metacognitive self-awareness after severe traumatic brain injury. *J Int Neuropsychol Soc, 14*(5), 862-868. doi:10.1017/S1355617708081125

Błądziński, P., Kalisz, A., Adamczyk, P., Arciszewska, A., Mętel, D., Daren, A., & Cechnicki, A. (2019). Associations of insight and treatment adherence with employment status of people with schizophrenia. *Postępy Psychiatrii i Neurologii, 28*(1), 21-33. doi:10.5114/ppn.2018.81364

Bota, R. G., Munro, J. S., Ricci, W. F., & Bota, D. A. (2006). The dynamics of insight in the prodrome of schizophrenia. *CNS Spectr, 11*(5), 355-362. doi:10.1017/s1092852900014486

Castrillo Sanz, A., Andres Calvo, M., Repiso Gento, I., Izquierdo Delgado, E., Gutierrez Rios, R., Rodriguez Herrero, R., . . . Tola-Arribas, M. A. (2016). Anosognosia in Alzheimer disease: Prevalence, associated factors, and influence on disease progression. *Neurologia, 31*(5), 296-304. doi:10.1016/j.nrl.2015.03.006

Cernovsky, Z. Z., Landmark, J. A., Merskey, H., & Husni, M. (2004). Clinical correlates of insight in schizophrenia. *Psychol Rep, 95*(3 Pt 1), 821-827. doi:10.2466/pr0.95.3.821-827

Chapman, S., Beschin, N., Cosentino, S., Elkind, M. S. V., Della Sala, S., & Cocchini, G. (2019). Anosognosia for prospective and retrospective memory deficits: Assessment and theoretical considerations. *Neuropsychology, 33*(7), 1020-1031. doi:10.1037/neu0000568

Chapman, S., Colvin, L. E., Vuorre, M., Cocchini, G., Metcalfe, J., Huey, E. D., & Cosentino, S. (2018). Cross domain self-monitoring in anosognosia for memory loss in Alzheimer's disease. *Cortex, 101*, 221-233. doi:10.1016/j.cortex.2018.01.019

Chapman, S., Cosentino, S., Igwe, K. C., Abdurahman, A., Elkind, M. S. V., Brickman, A. M., . . . Cocchini, G. (2020). Mnemonic monitoring in anosognosia for memory loss. *Neuropsychology, 34*(6), 675-685. doi:10.1037/neu0000643

Cherian, A. V., Narayanaswamy, J. C., Srinivasaraju, R., Viswanath, B., Math, S. B., Kandavel, T., & Reddy, Y. C. (2012). Does insight have specific correlation with symptom dimensions in OCD? *J Affect Disord, 138*(3), 352-359. doi:10.1016/j.jad.2012.01.017

Chesnel, C., Jourdan, C., Bayen, E., Ghout, I., Darnoux, E., Azerad, S., . . . Vallat-Azouvi, C. (2018). Self-awareness four years after severe traumatic brain injury: discordance between the patient's and relative's complaints. Results from the PariS-TBI study. *Clinical Rehabilitation, 32*(5), 692-704. doi:10.1177/0269215517734294

Cines, S., Farrell, M., Steffener, J., Sullo, L., Huey, E., Karlawish, J., & Cosentino, S. (2015). Examining the Pathways Between Self-Awareness and Well-Being in Mild to Moderate Alzheimer Disease. *Am J Geriatr Psychiatry, 23*(12), 1297-1306. doi:10.1016/j.jagp.2015.05.005

Ciurli, P., Bivona, U., Barba, C., Onder, G., Silvestro, D., Azicnuda, E., . . . Formisano, R. (2010). Metacognitive unawareness correlates with executive function impairment after severe traumatic brain injury. *J Int Neuropsychol Soc, 16*(2), 360-368. doi:10.1017/S135561770999141X

Clare, L., Whitaker, C. J., & Nelis, S. M. (2010). Appraisal of Memory Functioning and Memory Performance in Healthy Ageing and Early-Stage Alzheimer's Disease. *Aging Neuropsychology and Cognition, 17*(4), 462-491. doi:10.1080/13825580903581558

Cobo, J., Labad, J., Pousa, E., Nieto, L., Ochoa, S., Usall, J., . . . Ruiz, A. I. (2020). Exploring the relationship of insight with psychopathology and gender in individuals with schizophrenia spectrum disorders with structural equation modelling. *Arch Womens Ment Health, 23*(5), 643-655. doi:10.1007/s00737-020-01031-1

Conde-Sala, J. L., Reñé-Ramírez, R., Turró-Garriga, O., Gascón-Bayarri, J., Juncadella-Puig, M., Moreno-Cordón, L., . . . Garre-Olmo, J. (2013). Clinical differences in patients with Alzheimer's disease according to the presence or absence of anosognosia: implications for perceived quality of life. *Journal of Alzheimer's Disease, 33*(1), 1105-1116. doi:10.3233/JAD-2012-121360

Contador, I., Mograbi, D. C., Fernandez-Calvo, B., Benito-Leon, J., & Bermejo-Pareja, F. (2020). Comparison of mortality rate in older adults with and without functional awareness: the Neurological Disorders in Central Spain (NEDICES) population-based study. *Public Health, 183*, 146-152. doi:10.1016/j.puhe.2020.03.033

Cosentino, S., Metcalfe, J., Butterfield, B., Stern, Y., Cosentino, S., Metcalfe, J., . . . Stern, Y. (2007). Objective metamemory testing captures awareness of deficit in Alzheimer's disease. *Cortex: A Journal Devoted to the Study of the Nervous System & Behavior, 43*(7), 1004-1019. Retrieved from http://search.ebscohost.com/login.aspx?direct=true&db=cin20&AN=105818205&site=ehost-live
https://ac.els-cdn.com/S001094520870697X/1-s2.0-S001094520870697X-main.pdf?_tid=02ddf3af-b07c-4488-aafc-f93e700512e1&acdnat=1547815450_d0d974d1d887c4ef1763a356b472b069

Cosentino, S., Metcalfe, J., Cary, M. S., De Leon, J., & Karlawish, J. (2011). Memory Awareness Influences Everyday Decision Making Capacity about Medication Management in Alzheimer's Disease. *Int J Alzheimers Dis, 2011*, 483897. doi:10.4061/2011/483897

Cuffel, B. J., Alford, J., Fischer, E. P., & Owen, R. R. (1996). Awareness of illness in schizophrenia and outpatient treatment adherence. *J Nerv Ment Dis, 184*(11), 653-659. doi:10.1097/00005053-199611000-00001

Danki, D., Dilbaz, N., Okay, I. T., & Telci, S. (2007). [Insight in schizophrenia: relationship to family history, and positive and negative symptoms]. *Turk Psikiyatri Derg, 18*(2), 129-136. Retrieved from https://www.ncbi.nlm.nih.gov/pubmed/17566878

De Berardis, D., Campanella, D., Gambi, F., Sepede, G., Salini, G., Carano, A., . . . Ferro, F. M. (2005). Insight and alexithymia in adult outpatients with obsessive-compulsive disorder. *Eur Arch Psychiatry Clin Neurosci, 255*(5), 350-358. doi:10.1007/s00406-005-0573-y

De Berardis, D., Campanella, D., Serront, N., Gambi, F., Carano, A., La Rovere, R., . . . Ferro, F. M. (2008). Insight and perceived expressed emotion among adult outpatients with obsessive-compulsive disorder. *J Psychiatr Pract, 14*(3), 154-159. doi:10.1097/01.pra.0000320114.38434.5f

De Carolis, A., Cipollini, V., Corigliano, V., Comparelli, A., Sepe-Monti, M., Orzi, F., . . . Giubilei, F. (2015). Anosognosia in people with cognitive impairment: association with cognitive deficits and behavioral disturbances. *Dement Geriatr Cogn Dis Extra, 5*(1), 42-50. doi:10.1159/000367987

DeFeis, B., Chapman, S., Zhu, C., Azar, M., Sunderaraman, P., Ornstein, K., . . . Cosentino, S. (2019). Reduced Awareness of Memory Deficit is Associated With Increased Medicare Home Health Care Use in Dementia. *Alzheimer Dis Assoc Disord, 33*(1), 62-67. doi:10.1097/WAD.0000000000000287

Depp, C. A., Harmell, A. L., Savla, G. N., Mausbach, B. T., Jeste, D. V., & Palmer, B. W. (2014). A prospective study of the trajectories of clinical insight, affective symptoms, and cognitive ability in bipolar disorder. *Journal of Affective Disorders, 152-154*, 250-255. doi:10.1016/j.jad.2013.09.020

Derouesne, C., Thibault, S., Lagha-Pierucci, S., Baudouin-Madec, V., Ancri, D., & Lacomblez, L. (1999). Decreased awareness of cognitive deficits in patients with mild dementia of the Alzheimer type. *International Journal of Geriatric Psychiatry, 14*(12), 1019-1030. doi:10.1002/(SICI)1099-1166(199912)14:12<1019::AID-GPS61>3.0.CO;2-F

Dias, V., Brissos, S., & Carita, A. (2008). Clinical and neurocognitive correlates of insight in patients with bipolar I disorder in remission. *Acta Psychiatrica Scandinavica, 117*(1), 28-34. Retrieved from

https://openathens.ovid.com/secure-ssl/home.oa?idpselect=https://kclidp.kcl.ac.uk/idp/shibboleth&entityID=https://kclidp.kcl.ac.uk/idp/shibboleth&?T=JS&CSC=Y&NEWS=N&PAGE=fulltext&D=psyc6&AN=2007-19430-005
http://sfx.kcl.ac.uk/kings?sid=OVID:psycdb&id=pmid:&id=doi:&genre=article&atitle=Clinical+and+neurocognitive+correlates+of+insight+in+patients+with+bipolar+I+disorder+in+remission.&title=Acta+Psychiatrica+Scandinavica&issn=0001-690X&date=2008&volume=117&issue=1&spage=28&aulast=Dias%2C+V.+V&isbn=&__char_set=utf8
https://onlinelibrary.wiley.com/doi/pdf/10.1111/j.1600-0447.2007.01110.x

Dias, V. V., Brissos, S., Frey, B. N., & Kapczinski, F. (2008). Insight, quality of life and cognitive functioning in euthymic patients with bipolar disorder. *J Affect Disord, 110*(1-2), 75-83. doi:10.1016/j.jad.2008.01.010

Diez-Martin, J., Moreno-Ortega, M., Bagney, A., Rodriguez-Jimenez, R., Padilla-Torres, D., Sanchez-Morla, E. M., . . . Jimenez-Arriero, M. A. (2014). Differential relationships between set-shifting abilities and dimensions of insight in schizophrenia. *Psychopathology, 47*(2), 86-92. doi:10.1159/000348631

Donohoe, G., Donnell, C. O., Owens, N., & O'Callaghan, E. (2004). Evidence that health attributions and symptom severity predict insight in schizophrenia. *Journal of Nervous & Mental Disease, 192*(9), 635-637. Retrieved from http://search.ebscohost.com/login.aspx?direct=true&db=cin20&AN=106546444&site=ehost-live

Dourado, M., Marinho, V., Soares, C., Engelhardt, E., & Laks, J. (2007). Awareness of disease in Alzheimer's dementia: description of a mild to moderate sample of patient and caregiver dyads in Brazil. *International Psychogeriatrics, 19*(4), 733-744. doi:10.1017/S1041610207005492

Dourado, M. C. N., Laks, J., & Mograbi, D. C. (2019). Awareness in Dementia: Development and Evaluation of a Short Version of the Assessment Scale of Psychosocial Impact of the Diagnosis of Dementia (ASPIDD-s) in Brazil. *Alzheimer Dis Assoc Disord, 33*(3), 220-225. doi:10.1097/WAD.0000000000000306

Elvish, J., Simpson, J., & Ball, L. J. (2010). Which clinical and demographic factors predict poor insight in individuals with obsessions and/or compulsions? *J Anxiety Disord, 24*(2), 231-237. doi:10.1016/j.janxdis.2009.11.001

Emami, S., Guimond, S., Chakravarty, M. M., & Lepage, M. (2016). Cortical thickness and low insight into symptoms in enduring schizophrenia. *Schizophrenia research, 170*(1), 66-72. doi:10.1016/j.schres.2015.10.016

Faget-Agius, C., Boyer, L., Padovani, R., Richieri, R. l., Mundler, O., LanÃ§on, C., & Guedj, E. (2012). Schizophrenia with preserved insight is associated with increased perfusion of the precuneus. *Journal of Psychiatry & Neuroscience, 37*(5), 297-304. doi:10.1503/jpn.110125

Feher, E. P., Mahurin, R. K., Inbody, S. B., Crook, T. H., & Pirozzolo, F. J. (1991). Anosognosia in Alzheimer's disease. *Neuropsychiatry, Neuropsychology and Behavioral Neurology, 4*(2), 136-146. Retrieved from https://www.scopus.com/inward/record.uri?eid=2-s2.0-0026092160&partnerID=40&md5=6bd68c43b8e0168c95b209c81aa648cb

Fennig, S., Everett, E., Bromet, E. J., Jandorf, L., Fennig, S. R., Tanenberg-Karant, M., & Craig, T. J. (1996). Insight in first-admission psychotic patients. *Schizophr Res, 22*(3), 257-263. Retrieved from https://ac.els-cdn.com/S0920996496000771/1-s2.0-S0920996496000771-main.pdf?_tid=917f9b71-cd1e-476a-8025-526f1dccca7d&acdnat=1547815468_d25167b759211050b99d47d921873c5e

Fu, Y.-N., Cao, X.-L., Hou, C.-L., Ng, C. H., Ungvari, G. S., Chiu, H. F., . . . Xiang, Y.-T. (2017). Comparison of insight and clinical variables in homeless and non-homeless psychiatric inpatients in China. *Psychiatry Research, 255*, 13-16. Retrieved from https://openathens.ovid.com/secure-ssl/home.oa?idpselect=https://kclidp.kcl.ac.uk/idp/shibboleth&entityID=https://kclidp.kcl.ac.uk/idp/shibboleth&?T=JS&CSC=Y&NEWS=N&PAGE=fulltext&D=psyc13a&AN=2017-34965-004
http://sfx.kcl.ac.uk/kings?sid=OVID:psycdb&id=pmid:&id=doi:10.1016%2Fj.psychres.2017.04.066&genre=article&atitle=Comparison+of+insight+and+clinical+variables+in+homeless+and+non-homeless+psychiatric+inpatients+in+China.&title=Psychiatry+Research&issn=0165-1781&date=2017&volume=255&issue=&spage=13&aulast=Fu%2C+Yan-Nan&isbn=&__char_set=utf8
https://ac.els-cdn.com/S0165178117300057/1-s2.0-S0165178117300057-main.pdf?_tid=4a418933-e514-4e1f-8b42-49157ebf854e&acdnat=1549984131_02bd040bbac44b6df1027bd5001346a5

Fujimoto, H., Matsuoka, T., Kato, Y., Shibata, K., Nakamura, K., Yamada, K., & Narumoto, J. (2017). Brain regions associated with anosognosia for memory disturbance in Alzheimer's disease: a magnetic resonance imaging study. *Neuropsychiatr Dis Treat, 13*, 1753-1759. doi:10.2147/ndt.s139177

Gambina, G., Bonazzi, A., Valbusa, V., Condoleo, M., Bortolami, O., Broggio, E., . . . Moro, V. (2014). Awareness of cognitive deficits and clinical competence in mild to moderate Alzheimer's disease: their relevance in clinical practice. *Neurol Sci, 35*(3), 385-390. doi:10.1007/s10072-013-1523-5

Garg, R., Cheema, S. K., & Raj, R. (2018). Psychometric Properties of the Insight in Psychosis Questionnaire and its Correlation to Psychopathology in Indian Population. *Indian J Psychol Med, 40*(2), 113-120. doi:10.4103/ijpsym.ijpsym_112_17

Gerretsen, P., Chakravarty, M., Mamo, D., Menon, M., Pollock, B. G., Rajji, T. K., & Graff-Guerrero, A. (2013). Frontotemporoparietal asymmetry and lack of illness awareness in schizophrenia. *Human Brain Mapping, 34*(5), 1035-1043. Retrieved from https://openathens.ovid.com/secure-ssl/home.oa?idpselect=https://kclidp.kcl.ac.uk/idp/shibboleth&entityID=https://kclidp.kcl.ac.uk/idp/shibboleth&?T=JS&CSC=Y&NEWS=N&PAGE=fulltext&D=psyc10&AN=2013-12400-004

http://sfx.kcl.ac.uk/kings?sid=OVID:psycdb&id=pmid:&id=doi:10.1002%2Fhbm.21490&genre=article&atitle=Frontotemporoparietal+asymmetry+and+lack+of+illness+awareness+in+schizophrenia.&title=Human+Brain+Mapping&issn=1065-9471&date=2013&volume=34&issue=5&spage=1035&aulast=Gerretsen%2C+Philip&isbn=&__char_set=utf8

https://onlinelibrary.wiley.com/doi/pdf/10.1002/hbm.21490

Gerretsen, P., Chung, J. K., Shah, P., Plitman, E., Iwata, Y., Caravaggio, F., . . . Graff-Guerrero, A. (2017). Anosognosia Is an Independent Predictor of Conversion From Mild Cognitive Impairment to Alzheimer’s Disease and Is Associated With Reduced Brain Metabolism. *Journal of Clinical Psychiatry, 78*(9), e1187-e1196. Retrieved from https://openathens.ovid.com/secure-ssl/home.oa?idpselect=https://kclidp.kcl.ac.uk/idp/shibboleth&entityID=https://kclidp.kcl.ac.uk/idp/shibboleth&?T=JS&CSC=Y&NEWS=N&PAGE=fulltext&D=emexa&AN=620138877

http://sfx.kcl.ac.uk/kings?sid=OVID:embase&id=pmid:&id=doi:10.4088%2FJCP.16m11367&genre=article&atitle=Anosognosiag+pIS+an+independentpy+g+predictor+of+conversiony+From+mild+cognitive+impairment+to+Alzheimer%27s+disease+and+is+associated+with+reduced+brain+metabolism&title=Journal+of+Clinical+Psychiatry&issn=0160-6689&date=2017&volume=78&issue=9&spage=e1187&aulast=Gerretsen+P.&isbn=&__char_set=utf8

Ghaemi, S. N., Stoll, A. L., & Pope, H. G., Jr. (1995). Lack of insight in bipolar disorder. The acute manic episode. *J Nerv Ment Dis, 183*(7), 464-467.

Gilleen, J., Greenwood, K., Archer, N., Lovestone, S., & David, A. S. (2012). The role of premorbid personality and cognitive factors in awareness of illness, memory, and behavioural functioning in Alzheimer's disease. *Cognitive Neuropsychiatry, 17*(3), 227-245. doi:10.1080/13546805.2011.588007

Hamann, J., Kruse, J., Schmitz, F. S., Kissling, W., & Pajonk, F. G. (2010). Patient participation in antipsychotic drug choice decisions. *Psychiatry Res, 178*(1), 63-67. doi:10.1016/j.psychres.2008.08.008

Hannesdottir, K., Morris, R. G., Hannesdottir, K., & Morris, R. G. (2007). Primary and secondary anosognosia for memory impairment in patients with Alzheimer's disease. *Cortex: A Journal Devoted to the Study of the Nervous System & Behavior, 43*(7), 1020-1030. Retrieved from http://search.ebscohost.com/login.aspx?direct=true&db=cin20&AN=105818204&site=ehost-live

https://ac.els-cdn.com/S0010945208706981/1-s2.0-S0010945208706981-main.pdf?_tid=50741ec7-6f3e-49ea-bbf7-85783108a41f&acdnat=1549632223_2dca909c088067ec3207d8a608cffe54

Hanyu, H., Sato, T., Akai, T., Shimizu, S., Hirao, K., Kanetaka, H., . . . Koizumi, K. (2008). Neuroanatomical correlates of unawareness of memory deficits in early Alzheimer's disease. *Dement Geriatr Cogn Disord, 25*(4), 347-353. doi:10.1159/000119594

Heinrichs, D. W., Cohen, B. P., & Carpenter, W. T., Jr. (1985). Early insight and the management of schizophrenic decompensation. *J Nerv Ment Dis, 173*(3), 133-138.

Himle, J. A., Van Etten, M. L., Janeck, A. S., & Fischer, D. J. (2006). Insight as a Predictor of Treatment Outcome in Behavioral Group Treatment for Obsessive–Compulsive Disorder. *Cognitive Therapy and Research, 30*(5), 661-666. doi:10.1007/s10608-006-9079-9

Jacob, M. L., Larson, M. J., & Storch, E. A. (2014). Insight in adults with obsessive-compulsive disorder. *Compr Psychiatry, 55*(4), 896-903. doi:10.1016/j.comppsych.2013.12.016

Jeong, S. H., Chung, I. W., Jung, H. Y., Hwang, S. S., Kim, S. H., Youn, T., . . . Kim, Y. S. (2017). Comparison of clinician-rated and self-report insight in Korean patients with schizophrenia using VAGUS insight scale. *Psychiatry Res, 258*, 93-100. doi:10.1016/j.psychres.2017.10.003

Kalbe, E., Salmon, E., Perani, D., Holthoff, V., Sorbi, S., Elsner, A., . . . Herholz, K. (2005). Anosognosia in very mild Alzheimer's disease but not in mild cognitive impairment. *Dement Geriatr Cogn Disord, 19*(5-6), 349-356. doi:10.1159/000084704

Kao, Y.-C., & Liu, Y.-P. (2010). The clinical applicability of the Self-Appraisal of Illness Questionnaire (SAIQ) to chronic schizophrenic patients in Taiwan. *Psychiatric Quarterly, 81*(3), 215-225. Retrieved from https://openathens.ovid.com/secure-ssl/home.oa?idpselect=https://kclidp.kcl.ac.uk/idp/shibboleth&entityID=https://kclidp.kcl.ac.uk/idp/shibboleth&?T=JS&CSC=Y&NEWS=N&PAGE=fulltext&D=psyc7&AN=2010-17299-004

http://sfx.kcl.ac.uk/kings?sid=OVID:psycdb&id=pmid:&id=doi:10.1007%2Fs11126-010-9131-5&genre=article&atitle=The+clinical+applicability+of+the+Self-Appraisal+of+Illness+Questionnaire+%28SAIQ%29+to+chronic+schizophrenic+patients+in+Taiwan.&title=Psychiatric+Quarterly&issn=0033-2720&date=2010&volume=81&issue=3&spage=215&aulast=Kao%2C+Yu-Chen&isbn=&__char_set=utf8

https://link.springer.com/content/pdf/10.1007%2Fs11126-010-9131-5.pdf

Karadag, F., Tumkaya, S., Kirtas, D., Efe, M., Alacam, H., & Oguzhanoglu, N. K. (2011). Neurological soft signs in obsessive compulsive disorder with good and poor insight. *Prog Neuropsychopharmacol Biol Psychiatry, 35*(4), 1074-1079. doi:10.1016/j.pnpbp.2011.03.003

Karow, A., Pajonk, F. G., Reimer, J., Hirdes, F., Osterwald, C., Naber, D., & Moritz, S. (2008). The dilemma of insight into illness in schizophrenia: self- and expert-rated insight and quality of life. *Eur Arch Psychiatry Clin Neurosci, 258*(3), 152-159. doi:10.1007/s00406-007-0768-5

Kashyap, H., Kumar, J. K., Kandavel, T., & Reddy, Y. C. J. (2012). Neuropsychological correlates of insight in obsessive-compulsive disorder. *Acta Psychiatrica Scandinavica, 126*(2), 106-114. doi:10.1111/j.1600-0447.2012.01845.x

Kazui, H., Hirono, N., Hashimoto, M., Nakano, Y., Matsumoto, K., Takatsuki, Y., . . . Takeda, M. (2006). Symptoms underlying unawareness of memory impairment in patients with mild Alzheimer's disease. *Journal of Geriatric Psychiatry and Neurology, 19*(1), 3-12. doi:10.1177/0891988705277543

Kelly, B., Clarke, M., Browne, S., McTigue, O., Kamali, M., Gervin, M., . . . O'Callaghan, E. (2004). Clinical predictors of admission status in first episode schizophrenia. *European Psychiatry, 19*(2), 67-71. Retrieved from https://openathens.ovid.com/secure-ssl/home.oa?idpselect=https://kclidp.kcl.ac.uk/idp/shibboleth&entityID=https://kclidp.kcl.ac.uk/idp/shibboleth&?T=JS&CSC=Y&NEWS=N&PAGE=fulltext&D=psyc4&AN=2005-07074-001

http://sfx.kcl.ac.uk/kings?sid=OVID:psycdb&id=pmid:&id=doi:10.1016%2Fj.eurpsy.2003.07.009&genre=article&atitle=Clinical+predictors+of+admission+status+in+first+episode+schizophrenia.&title=European+Psychiatry&issn=0924-9338&date=2004&volume=19&issue=2&spage=67&aulast=Kelly%2C+B.+D&isbn=&__char_set=utf8

https://ac.els-cdn.com/S0924933803001767/1-s2.0-S0924933803001767-main.pdf?_tid=9a880f66-28b6-481a-a2ac-b2db5f2f0a79&acdnat=1547130435_7071e28b99618e5f60178caa1e3f40d2

Kishore, V. R., Samar, R., Reddy, Y. C. J., Chandrasekhar, C. R., & Thennarasu, K. (2004). Clinical characteristics and treatment response in poor and good insight obsessive-compulsive disorder. *European Psychiatry, 19*(4), 202-208. doi:10.1016/j.eurpsy.2003.12.005

Kleim, B., Vauth, R., Adam, G., Stieglitz, R.-D., Hayward, P., & Corrigan, P. (2009). Perceived stigma predicts low self-efficacy and poor coping in schizophrenia. *Journal of Mental Health, 17*(5), 482-491. doi:10.1080/09638230701506283

Konstantakopoulos, G., Ploumpidis, D., Oulis, P., Soumani, A., Nikitopoulou, S., Pappa, K., . . . David, A. S. (2013). Is insight in schizophrenia multidimensional? Internal structure and associations of the Greek version of the Schedule for the Assessment of Insight-Expanded. *Psychiatry Res, 209*(3), 346-352. doi:10.1016/j.psychres.2013.02.016

Kortte, K. B., McWhorter, J. W., Pawlak, M. A., Slentz, J., Sur, S., & Hillis, A. E. (2015). Anosognosia for hemiplegia: The contributory role of right inferior frontal gyrus. *Neuropsychology, 29*(3), 421-432. doi:10.1037/neu0000135

Lacerda, I. B., Santos, R. L., Belfort, T., Neto, J. P. S., & Dourado, M. C. N. (2018). Patterns of discrepancies in different objects of awareness in mild and moderate Alzheimer's disease. *Aging Ment Health*, 1-8. doi:10.1080/13607863.2018.1544219

Lacerda, I. B., Santos, R. L., Neto, J. P. S., & Dourado, M. C. N. (2017). Factors Related to Different Objects of Awareness in Alzheimer Disease. *Alzheimer Disease & Associated Disorders, 31*(4), 335-342. doi:10.1097/WAD.0000000000000210

Lamar, M., Lasarev, M. R., & Libon, D. J. (2002). Determining levels of unawareness in dementia research. *J Neuropsychiatry Clin Neurosci, 14*(4), 430-437. doi:10.1176/jnp.14.4.430

Loebel, J., Dager, S. R., Berg, G., & Hyde, T. S. (1990). Fluency of speech and self-awareness of memory deficit in Alzheimer's disease. *International Journal of Geriatric Psychiatry, 5*(1), 41-45. Retrieved from https://openathens.ovid.com/secure-ssl/home.oa?idpselect=https://kclidp.kcl.ac.uk/idp/shibboleth&entityID=https://kclidp.kcl.ac.uk/idp/shibboleth&?T=JS&CSC=Y&NEWS=N&PAGE=fulltext&D=psyc3&AN=1990-17632-001

http://sfx.kcl.ac.uk/kings?sid=OVID:psycdb&id=pmid:&id=doi:10.1002%2Fgps.930050107&genre=article&atitle=Fluency+of+speech+and+self-awareness+of+memory+deficit+in+Alzheimer%27s+disease.&title=International+Journal+of+Geriatric+Psychiatry&issn=0885-6230&date=1990&volume=5&issue=1&spage=41&aulast=Loebel%2C+J.+Pierre&isbn=&__char_set=utf8

https://onlinelibrary.wiley.com/doi/pdf/10.1002/gps.930050107

Lopez, O. L., Becker, J. T., Somsak, D., Dew, M. A., & Dekosky, S. T. (1994). Awareness of Cognitive Deficits and Anosognosia in Probable Alzheimers-Disease. *European Neurology, 34*(5), 277-282. doi:10.1159/000117056

Lysaker, P. H., Bell, M. D., Bryson, G. J., & Kaplan, E. (1998). Insight and interpersonal function in schizophrenia. *J Nerv Ment Dis, 186*(7), 432-436.

Macpherson, R., Jerrom, B., & Hughes, A. (1996). Relationship between insight, educational background and cognition in schizophrenia. *Br J Psychiatry, 168*(6), 718-722. doi:10.1192/bjp.168.6.718

Maeshima, S., Dohi, N., Funahashi, K., Nakai, K., Itakura, T., & Komai, N. (1997). Rehabilitation of patients with anosognosia for hemiplegia due to intracerebral haemorrhage. *Brain Inj, 11*(9), 691-697. Retrieved from https://www.tandfonline.com/doi/pdf/10.1080/026990597123232?needAccess=true

Martyr, A., Nelis, S. M., & Clare, L. (2014). Predictors of perceived functional ability in early-stage dementia: self-ratings, informant ratings and discrepancy scores. *Int J Geriatr Psychiatry, 29*(8), 852-862. doi:10.1002/gps.4071

Mayelle, A., El Haj, M., & Antoine, P. (2019). Awareness of Self and Disease Assessment: Development and Validation of a Subjective Measure in People with Alzheimer's Disease. *J Alzheimers Dis, 71*(3), 841-850. doi:10.3233/JAD-190371

McEvoy, J. P., Johnson, J., Perkins, D., Lieberman, J. A., Hamer, R. M., Keefe, R. S., . . . Sharma, T. (2006). Insight in first-episode psychosis. *Psychological Medicine, 36*(10), 1385-1393. doi:10.1017/S0033291706007793

Michel, P., Baumstarck, K., Auquier, P., Amador, X., Dumas, R., Fernandez, J., . . . Boyer, L. (2013). Psychometric properties of the abbreviated version of the a scale to assess unawareness in mental disorder in schizophrenia. *BMC Psychiatry Vol 13 2013, ArtID 229, 13*. Retrieved from https://openathens.ovid.com/secure-ssl/home.oa?idpselect=https://kclidp.kcl.ac.uk/idp/shibboleth&entityID=https://kclidp.kcl.ac.uk/idp/shibboleth&?T=JS&CSC=Y&NEWS=N&PAGE=fulltext&D=psyc10&AN=2014-08645-001

http://sfx.kcl.ac.uk/kings?sid=OVID:psycdb&id=pmid:&id=doi:10.1186%2F1471-244X-13-229&genre=article&atitle=Psychometric+properties+of+the+abbreviated+version+of+the+a+scale+to+assess+unawareness+in+mental+disorder+in+schizophrenia.&title=BMC+Psychiatry&issn=1471-244X&date=2013&volume=13&issue=1&spage=229&aulast=Michel%2C+Pierre&isbn=&__char_set=utf8

Mohamed, S., Rosenheck, R., McEvoy, J., Swartz, M., Stroup, S., & Lieberman, J. A. (2009). Cross-sectional and longitudinal relationships between insight and attitudes toward medication and clinical outcomes in chronic schizophrenia. *Schizophr Bull, 35*(2), 336-346. doi:10.1093/schbul/sbn067

Molina-Andreu, O., Gonzalez-Rodriguez, A., Villanueva, A. P., Penades, R., Catalan, R., & Bernardo, M. (2014). Awareness of illness and suicidal behavior in delusional disorder patients. *Revista de Psiquiatria Clinica, 41*(6), 156-158. Retrieved from https://openathens.ovid.com/secure-ssl/home.oa?idpselect=https://kclidp.kcl.ac.uk/idp/shibboleth&entityID=https://kclidp.kcl.ac.uk/idp/shibboleth&?T=JS&CSC=Y&NEWS=N&PAGE=fulltext&D=emed15&AN=601960184

http://sfx.kcl.ac.uk/kings?sid=OVID:embase&id=pmid:&id=doi:10.1590%2F0101-60830000000034&genre=article&atitle=Awareness+of+illness+and+suicidal+behavior+in+delusional+disorder+patients&title=Revista+de+Psiquiatria+Clinica&issn=0101-6083&date=2014&volume=41&issue=6&spage=156&aulast=Molina-Andreu+O.&isbn=&__char_set=utf8

Moro, V., Pernigo, S., Tsakiris, M., Avesani, R., Edelstyn, N. M., Jenkinson, P. M., & Fotopoulou, A. (2016). Motor versus body awareness: Voxel-based lesion analysis in anosognosia for hemiplegia and somatoparaphrenia following right hemisphere stroke. *Cortex, 83*, 62-77. doi:10.1016/j.cortex.2016.07.001

Noe, E., Ferri, J., Caballero, M. C., Villodre, R., Sanchez, A., & Chirivella, J. (2005). Self-awareness after acquired brain injury--predictors and rehabilitation. *J Neurol, 252*(2), 168-175. doi:10.1007/s00415-005-0625-2

Onen, S., Karakas Ugurlu, G., & Caykoylu, A. (2013). The relationship between metacognitions and insight in obsessive-compulsive disorder. *Compr Psychiatry, 54*(5), 541-548. doi:10.1016/j.comppsych.2012.11.006

Orfei, M. D., Varsi, A. E., Blundo, C., Celia, E., Casini, A. R., Caltagirone, C., . . . Spalletta, G. (2010). Anosognosia in mild cognitive impairment and mild Alzheimer's disease: frequency and neuropsychological correlates. *American Journal of Geriatric Psychiatry, 18*(12), 1133-1140. doi:10.1097/JGP.0b013e3181dd1c50

Othman, Z., & Huri, S. Z. (2017). Insight in Offenders with Schizophrenia: Relationship to Psychopathology and Cognitive Function. *International Medical Journal, 24*(1), 18-20. Retrieved from http://search.ebscohost.com/login.aspx?direct=true&db=cin20&AN=122041318&site=ehost-live

Ozkiris, A., Essizoglu, A., Gulec, G., & Aksaray, G. (2015). The relationship between insight and the level of expressed emotion in patients with obsessive-compulsive disorder. *Nord J Psychiatry, 69*(3), 204-209. doi:10.3109/08039488.2014.959996

Ozzoude, M., Nakajima, S., Plitman, E., Chung, J. K., Kim, J., Iwata, Y., . . . Gerretsen, P. (2019). The effects of illness severity, cognition, and estimated antipsychotic dopamine receptor occupancy on insight into the illness in schizophrenia: An analysis of clinical antipsychotic trials of intervention effectiveness (CATIE) data. *Prog Neuropsychopharmacol Biol Psychiatry, 89*, 207-213. doi:10.1016/j.pnpbp.2018.08.033

Prus, L., Wiedl, K. H., & Waldorf, M. (2012). Stigma as a predictor of insight in schizophrenia. *Psychiatry Research, 198*(2), 187-193. Retrieved from https://openathens.ovid.com/secure-ssl/home.oa?idpselect=https://kclidp.kcl.ac.uk/idp/shibboleth&entityID=https://kclidp.kcl.ac.uk/idp/shibboleth&?T=JS&CSC=Y&NEWS=N&PAGE=fulltext&D=psyc9&AN=2012-06495-001

http://sfx.kcl.ac.uk/kings?sid=OVID:psycdb&id=pmid:&id=doi:10.1016%2Fj.psychres.2011.12.012&genre=article&atitle=Stigma+as+a+predictor+of+insight+in+schizophrenia.&title=Psychiatry+Research&issn=0165-1781&date=2012&volume=198&issue=2&spage=187&aulast=Prus%2C+Linda&isbn=&__char_set=utf8

Rathod, S., Kingdon, D., Smith, P., & Turkington, D. (2005). Insight into schizophrenia: The effects of cognitive behavioural therapy on the components of insight and association with sociodemographics--data on a previously published randomised controlled trial. *Schizophrenia research, 74*(2-3), 211-219. Retrieved from https://openathens.ovid.com/secure-ssl/home.oa?idpselect=https://kclidp.kcl.ac.uk/idp/shibboleth&entityID=https://kclidp.kcl.ac.uk/idp/shibboleth&?T=JS&CSC=Y&NEWS=N&PAGE=fulltext&D=psyc4&AN=2005-03332-009

http://sfx.kcl.ac.uk/kings?sid=OVID:psycdb&id=pmid:&id=doi:10.1016%2Fj.schres.2004.07.003&genre=article&atitle=Insight+into+schizophrenia%3A+The+effects+of+cognitive+behavioural+therapy+on+the+components+of+insight+and+association+with+sociodemographics--data+on+a+previously+published+randomised+controlled+trial.&title=Schizophrenia+Research&issn=0920-9964&date=2005&volume=74&issue=2-3&spage=211&aulast=Rathod%2C+Shanaya&isbn=&__char_set=utf8

https://ac.els-cdn.com/S0920996404002233/1-s2.0-S0920996404002233-main.pdf?_tid=090b4739-3e31-4323-835a-1f58ab28e47c&acdnat=1547131013_ffc875fb50acc8d9cb6a191318545616

Rossell, S. L., Coakes, J., Shapleske, J., Woodruff, P. W. R., & David, A. S. (2003). Insight: its relationship with cognitive function, brain volume and symptoms in schizophrenia. *Psychological Medicine, 33*(1), 111-119. doi:10.1017/S0033291702006803

Schennach, R., Meyer, S., Seemuller, F., Jager, M., Schmauss, M., Laux, G., . . . Riedel, M. (2012). Insight in schizophrenia-course and predictors during the acute treatment phase of patients suffering from a schizophrenia spectrum disorder. *Eur Psychiatry, 27*(8), 625-633. doi:10.1016/j.eurpsy.2012.01.001
Sedaghat, F., Dedousi, E., Baloyannis, I., Tegos, T., Costa, V., Dimitriadis, A. S., . . . Baloyannis, S. J. (2010). Brain SPECT findings of anosognosia in Alzheimer's disease. *Journal of Alzheimer's Disease, 21*(2), 641-647. doi:10.3233/JAD-2010-090631
Senturk, G., Hanagasi, H., Gurvit, H., Emre, M., Bilgic, B., Bayram, A., & Arslan, A. B. (2017). Cognitive and anatomical correlates of anosognosia in amnestic mild cognitive impairment and early-stage Alzheimer's disease. *International Psychogeriatrics, 29*(2), 293-302. doi:10.1017/S1041610216001812
Shad, M. U., Muddasani, S., Prasad, K., Sweeney, J. A., & Keshavan, M. S. (2004). Insight and prefrontal cortex in first-episode Schizophrenia. *Neuroimage, 22*(3), 1315-1320. doi:10.1016/j.neuroimage.2004.03.016
Shimshoni, Y. a., Reuven, O., Dar, R., & Hermesh, H. (2011). Insight in obsessive-compulsive disorder: A comparative study of insight measures in an israeli clinical sample. *Journal of Behavior Therapy and Experimental Psychiatry, 42*(3), 389-396. Retrieved from https://openathens.ovid.com/secure-ssl/home.oa?idpselect=https://kclidp.kcl.ac.uk/idp/shibboleth&entityID=https://kclidp.kcl.ac.uk/idp/shibboleth&?T=JS&CSC=Y&NEWS=N&PAGE=fulltext&D=psyc8&AN=2011-09161-022
http://sfx.kcl.ac.uk/kings?sid=OVID:psycdb&id=pmid:&id=doi:10.1016%2Fj.jbtep.2011.02.011&genre=article&atitle=Insight+in+obsessive-compulsive+disorder%3A+A+comparative+study+of+insight+measures+in+an+israeli+clinical+sample.&title=Journal+of+Behavior+Therapy+and+Experimental+Psychiatry&issn=0005-7916&date=2011&volume=42&issue=3&spage=389&aulast=Shimshoni%2C+Ya%27ara&isbn=&__char_set=utf8
https://ac.els-cdn.com/S0005791611000346/1-s2.0-S0005791611000346-main.pdf?_tid=f5b9fad9-753f-4037-b009-10380924ca40&acdnat=1547133226_07b140763c3c5f3aa1ec63e4e7f65447
Silva, M. R., Moser, D., Pfluger, M., Pusswald, G., Stogmann, E., Dal-Bianco, P., . . . Lehrner, J. (2016). Self-reported and informant-reported memory functioning and awareness in patients with mild cognitive impairment and Alzheimer s disease. *Neuropsychiatr, 30*(2), 103-112. doi:10.1007/s40211-016-0185-y
Sitman, R., Sela, T., Erez, G., Bloch, Y., & Levkovitz, Y. (2012). Comparison of insight among schizophrenia and bipolar disorder patients in remission of affective and positive symptoms: Analysis and critique. *European Psychiatry, 27*(8), 612-618. Retrieved from https://openathens.ovid.com/secure-ssl/home.oa?idpselect=https://kclidp.kcl.ac.uk/idp/shibboleth&entityID=https://kclidp.kcl.ac.uk/idp/shibboleth&?T=JS&CSC=Y&NEWS=N&PAGE=fulltext&D=emed13&AN=51417140
http://sfx.kcl.ac.uk/kings?sid=OVID:embase&id=pmid:&id=doi:10.1016%2Fj.eurpsy.2011.02.002&genre=article&atitle=Comparison+of+insight+among+schizophrenia+and+bipolar+disorder+patients+in+remission+of+affective+and+positive+symptoms%3A+Analysis+and+critique&title=European+Psychiatry&issn=0924-9338&date=2012&volume=27&issue=8&spage=612&aulast=Braw+Y.&isbn=&__char_set=utf8
Sousa, M. F. B., Santos, R. L., Nogueira, M. L., Belfort, T., Rosa, R. D. L., Torres, B., . . . Dourado, M. C. N. (2015). Awareness of Disease is Different for Cognitive and Functional Aspects in Mild Alzheimer's Disease: A One-Year Observation Study. *Journal of Alzheimers Disease, 43*(3), 905-913. doi:10.3233/JAD-140342
Spalletta, G., Girardi, P., Caltagirone, C., & Orfei, M. D. (2012). Anosognosia and neuropsychiatric symptoms and disorders in mild Alzheimer disease and mild cognitive impairment. *J Alzheimers Dis, 29*(4), 761-772. doi:10.3233/jad-2012-111886
Starkstein, S. E., Brockman, S., Bruce, D., & Petracca, G. (2010). Anosognosia is a significant predictor of apathy in Alzheimer's disease. *J Neuropsychiatry Clin Neurosci, 22*(4), 378-383. doi:10.1176/appi.neuropsych.22.4.378
10.1176/jnp.2010.22.4.378
Starkstein, S. E., Jorge, R., Mizrahi, R., Robinson, R. G., Starkstein, S. E., Jorge, R., . . . Robinson, R. G. (2006). A diagnostic formulation for anosognosia in Alzheimer's disease. *Journal of Neurology, Neurosurgery & Psychiatry, 77*(6), 719-725. Retrieved from http://search.ebscohost.com/login.aspx?direct=true&db=cin20&AN=106229495&site=ehost-live
https://jnnp.bmj.com/content/jnnp/77/6/719.full.pdf
Starkstein, S. E., Sabe, L., Cuerva, A. G., Kuzis, G., & Leiguarda, R. (1997). Anosognosia and procedural learning in Alzheimer's disease. *Neuropsychiatry Neuropsychol Behav Neurol, 10*(2), 96-101.
Szepietowska, E. M., & Kuzaka, A. (2019). Self-assessment of executive function and lateralization of brain pathology: What does the DEX-S profile show? *Psychiatr Pol, 53*(1), 129-143. doi:10.12740/PP/OnlineFirst/85936

Therriault, J., Ng, K. P., Pascoal, T. A., Mathotaarachchi, S., Kang, M. S., Struyfs, H., . . . Rosa-Neto, P. (2018). Anosognosia predicts default mode network hypometabolism and clinical progression to dementia. *Neurology, 90*(11), E932-501. doi:10.1212/WNL.0000000000005120

Tolin, D. F., Fitch, K. E., Frost, R. O., & Steketee, G. (2008). Family Informants' Perceptions of Insight in Compulsive Hoarding. *Cognitive Therapy and Research, 34*(1), 69-81. doi:10.1007/s10608-008-9217-7

Tordesillas-Gutierrez, D., Ayesa-Arriola, R., Delgado-Alvarado, M., Robinson, J. L., Lopez-Morinigo, J., Pujol, J., . . . Crespo-Facorro, B. (2018). The right occipital lobe and poor insight in first-episode psychosis. *PLoS One, 13*(6), e0197715. doi:10.1371/journal.pone.0197715

Tremont, G., & Alosco, M. L. (2011). Relationship between cognition and awareness of deficit in mild cognitive impairment. *Int J Geriatr Psychiatry, 26*(3), 299-306. doi:10.1002/gps.2529

Tumkaya, S., Hanci Yenigun, E., Topak, O. Z., Sendur, I., Ozturk Atkaya, N., & Ozdel, O. (2019). [Is Clinical Insight Associated with Working Memory Components in Schizophrenia and Schizoaffective Disorder?]. *Turk Psikiyatri Derg, 30*(1), 1-8. Retrieved from https://www.ncbi.nlm.nih.gov/pubmed/31170301

Turksoy, N., Tukel, R., Ozdemir, O., & Karali, A. (2002). Comparison of clinical characteristics in good and poor insight obsessive-compulsive disorder. *J Anxiety Disord, 16*(4), 413-423. Retrieved from https://ac.els-cdn.com/S0887618502001354/1-s2.0-S0887618502001354-main.pdf?_tid=ebe45538-fa9b-458d-966b-331edacc2abf&acdnat=1547825067_b566812e3328e3d8d061715dd9b31afa

Turró-Garriga, O., Garre-Olmo, J., Calvó-Perxas, L., Reñé-Ramírez, R., Gascón-Bayarri, J., Conde-Sala, J. L., & Conde-Sala, J. L. (2016). Course and Determinants of Anosognosia in Alzheimer's Disease: A 12-Month Follow-up. *Journal of Alzheimer's Disease, 51*(2), 357-366. doi:10.3233/JAD-150706

Turro-Garriga, O., Garre-Olmo, J., Vilalta-Franch, J., Conde-Sala, J. L., Gracia Blanco, M., & Lopez-Pousa, S. (2013). Burden associated with the presence of anosognosia in Alzheimer's disease. *International Journal of Geriatric Psychiatry, 28*(3), 291-297. Retrieved from https://openathens.ovid.com/secure-ssl/home.oa?idpselect=https://kclidp.kcl.ac.uk/idp/shibboleth&entityID=https://kclidp.kcl.ac.uk/idp/shibboleth&?T=JS&CSC=Y&NEWS=N&PAGE=fulltext&D=psyc10&AN=2013-03935-010

http://sfx.kcl.ac.uk/kings?sid=OVID:psycdb&id=pmid:&id=doi:10.1002%2Fgps.3824&genre=article&atitle=Burden+associated+with+the+presence+of+anosognosia+in+Alzheimer%27s+disease.&title=International+Journal+of+Geriatric+Psychiatry&issn=0885-6230&date=2013&volume=28&issue=3&spage=291&aulast=Turro-Garriga%2C+Oriol&isbn=&__char_set=utf8

https://onlinelibrary.wiley.com/doi/pdf/10.1002/gps.3824

Valiente, C., Provencio, M., Espinosa, R., Chaves, C., & Fuentenebro, F. (2011). Predictors of subjective well-being in patients with paranoid symptoms: is insight necessarily advantageous? *Psychiatry Res, 189*(2), 190-194. doi:10.1016/j.psychres.2011.02.018

Vasterling, J. J., Seltzer, B., Carpenter, B. D., & Thompson, K. A. (1997). Unawareness of social interaction and emotional control deficits in alzheimer's disease. *Neuropsychol Dev Cogn B Aging Neuropsychol Cogn, 4*(4), 280-289. doi:10.1080/13825589708256653

Vasterling, J. J., Seltzer, B., Foss, J. W., & Vanderbrook, V. (1995). Unawareness of deficit in Alzheimer's disease: Domain-specific differences and disease correlates. *Neuropsychiatry, Neuropsychology, & Behavioral Neurology, 8*(1), 26-32. Retrieved from https://openathens.ovid.com/secure-ssl/home.oa?idpselect=https://kclidp.kcl.ac.uk/idp/shibboleth&entityID=https://kclidp.kcl.ac.uk/idp/shibboleth&?T=JS&CSC=Y&NEWS=N&PAGE=fulltext&D=psyc3&AN=1995-41126-001

http://sfx.kcl.ac.uk/kings?sid=OVID:psycdb&id=pmid:&id=doi:&genre=article&atitle=Unawareness+of+deficit+in+Alzheimer%27s+disease%3A+Domain-specific+differences+and+disease+correlates.&title=Neuropsychiatry%2C+Neuropsychology%2C+%26+Behavioral+Neurology&issn=0894-878X&date=1995&volume=8&issue=1&spage=26&aulast=Vasterling%2C+Jennifer+J&isbn=&__char_set=utf8

Verhey, F. R. J., Rozendaal, N., Ponds, R. W. H. M., & Jolles, J. (1993). Dementia, awareness and depression. *International Journal of Geriatric Psychiatry, 8*(10), 851-856. doi:10.1002/gps.930081008

Verhulsdonk, S., Quack, R., HÃ¶ft, B., Lange-Asschenfeldt, C., & Supprian, T. (2013). Anosognosia and depression in patients with Alzheimer's dementia. *Archives of Gerontology & Geriatrics, 57*(3), 282-287. doi:10.1016/j.archger.2013.03.012

Visser, H., Megen, H. V., Oppen, P. V., Hoogendoorn, A., Glas, G., Neziroglu, F., & Balkom, A. V. (2017). The impact of poor insight on the course of Obsessive-Compulsive Disorder in patients receiving naturalistic treatment. *Journal of Obsessive-Compulsive and Related Disorders, 13*, 42-48. Retrieved

from https://openathens.ovid.com/secure-ssl/home.oa?idpselect=https://kclidp.kcl.ac.uk/idp/shibboleth&entityID=https://kclidp.kcl.ac.uk/idp/shibboleth&?T=JS&CSC=Y&NEWS=N&PAGE=fulltext&D=emed18&AN=615380094
http://sfx.kcl.ac.uk/kings?sid=OVID:embase&id=pmid:&id=doi:10.1016%2Fj.jocrd.2017.03.003&genre=article&atitle=The+impact+of+poor+insight+on+the+course+of+Obsessive-Compulsive+Disorder+in+patients+receiving+naturalistic+treatment&title=Journal+of+Obsessive-Compulsive+and+Related+Disorders&issn=2211-3649&date=2017&volume=13&issue=&spage=42&aulast=Visser+H.&isbn=&__char_set=utf8

Wang, Y., Xiang, Y. T., Wang, C. Y., Chiu, H. F., Zhao, J. P., Chen, Q., . . . Ungvari, G. S. (2011). Insight in Chinese schizophrenia patients: a 12-month follow-up. *J Psychiatr Ment Health Nurs, 18*(9), 751-757. doi:10.1111/j.1365-2850.2010.01677.x

Weiler, M. A., Fleisher, M. H., & McArthur-Campbell, D. (2000). Insight and symptom change in schizophrenia and other disorders. *Schizophr Res, 45*(1-2), 29-36. doi:10.1016/s0920-9964(99)00215-7

Wibawa, P., Zombor, R., Dragovic, M., Hayhow, B., Lee, J., Panegyres, P. K., . . . Starkstein, S. E. (2020). Anosognosia Is Associated With Greater Caregiver Burden and Poorer Executive Function in Huntington Disease. *J Geriatr Psychiatry Neurol, 33*(1), 52-58. doi:10.1177/0891988719856697

Woon, L. S., Khoo, S. I., Baharudin, A., & Midin, M. (2020). Association between insight and internalized stigma and other clinical factors among patients with depression: A cross-sectional study. *Indian J Psychiatry, 62*(2), 186-192. doi:10.4103/psychiatry.IndianJPsychiatry_612_19

Xiang, Y. T., Wang, Y., Wang, C. Y., Chiu, H. F., Chen, Q., Chan, S. S., . . . Ungvari, G. S. (2012). Association of insight with sociodemographic and clinical factors, quality of life, and cognition in Chinese patients with schizophrenia. *Compr Psychiatry, 53*(2), 140-144. doi:10.1016/j.comppsych.2011.04.001

Yeh, Y. C., Yen, C. F., Li, C. W., Kuo, Y. T., Chen, C. H., Lee, C. C., . . . Chen, C. S. (2014). Altered neurochemical metabolites in Alzheimer's disease patients with unawareness of deficits. *Int Psychogeriatr, 26*(3), 393-402. doi:10.1017/S1041610213001944

Yen, C. F., Cheng, C. P., Ko, C. H., Yen, J. Y., Huang, C. F., & Chen, C. S. (2008). Relationship between insight and neurocognition in patients with bipolar I disorder in remission. *Compr Psychiatry, 49*(4), 335-339. doi:10.1016/j.comppsych.2007.12.006

Yoon, B., Shim, Y. S., Hong, Y. J., Choi, S. H., Park, H. K., Park, S. A., . . . Yang, D.-W. (2017). Anosognosia and Its Relation to Psychiatric Symptoms in Early-Onset Alzheimer Disease. *Journal of Geriatric Psychiatry & Neurology, 30*(3), 170-177. doi:10.1177/0891988717700508

Zhang, Q., Li, X., Parker, G. J., Hong, X. H., Wang, Y., Lui, S. S. Y., . . . Chan, R. C. K. (2016). Theory of mind correlates with clinical insight but not cognitive insight in patients with schizophrenia. *Psychiatry Research, 237*, 188-195. doi:10.1016/j.psychres.2016.01.044

Qualitative Only

Aalten, P., van Valen, E., de Vugt, M. E., Lousberg, R., Jolles, J., & Verhey, F. R. J. (2006). Awareness and behavioral problems in dementia patients: a prospective study. *International Psychogeriatrics, 18*(1), 3-17. doi:10.1017/S1041610205002772

Almeida, O. P., Levy, R., Howard, R. J., & David, A. S. (1996). Insight and paranoid disorders in late life (late paraphenia). *International Journal of Geriatric Psychiatry, 11*(7), 653-658. doi:10.1002/(sici)1099-1166(199607)11:7<653::Aid-gps380>3.0.Co;2-9

Amador, X. F., Flaum, M., Andreasen, N. C., Strauss, D. H., Yale, S. A., Clark, S. C., & Gorman, J. M. (1994). Awareness of illness in schizophrenia and schizoaffective and mood disorders. *Arch Gen Psychiatry, 51*(10), 826-836. Retrieved from https://jamanetwork.com/journals/jamapsychiatry/articlepdf/496809/archpsyc_51_10_007.pdf

Arbel, R., Koren, D., Klein, E., & Latzer, Y. (2013). The neurocognitive basis of insight into illness in anorexia nervosa: a pilot metacognitive study. *Psychiatry Res, 209*(3), 604-610. doi:10.1016/j.psychres.2013.01.009

Berg, A. O., Barrett, E. A., Nerhus, M., Buchman, C., Simonsen, C., Faerden, A., . . . Melle, I. (2018). Psychosis: clinical insight and beliefs in immigrants in their first episode. *Early Interv Psychiatry, 12*(2), 185-192. doi:10.1111/eip.12297

Bianchini, O., Porcelli, S., Nespeca, C., Cannavo, D., Trappoli, A., Aguglia, E., . . . Serretti, A. (2014). Effects of antipsychotic drugs on insight in schizophrenia. *Psychiatry Res, 218*(1-2), 20-24. doi:10.1016/j.psychres.2014.03.022

Buchy, L., Bodnar, M., Malla, A., Joober, R., & Lepage, M. (2010). A 12-month outcome study of insight and symptom change in first-episode psychosis. *Early Interv Psychiatry, 4*(1), 79-88. doi:10.1111/j.1751-7893.2010.00166.x

Burton, C. Z., Harvey, P. D., Patterson, T. L., & Twamley, E. W. (2016). Neurocognitive insight and objective cognitive functioning in schizophrenia. *Schizophr Res, 171*(1-3), 131-136. doi:10.1016/j.schres.2016.01.021

Chan, K. K. (2016). Associations of symptoms, neurocognition, and metacognition with insight in schizophrenia spectrum disorders. *Compr Psychiatry, 65*, 63-69. doi:10.1016/j.comppsych.2015.09.009

Chan, S. K. W., Chan, K. K. S., Hui, C. L., Wong, G. H. Y., Chang, W. C., Lee, E. H. M., . . . Chen, E. Y. H. (2014). Correlates of insight with symptomatology and executive function in patients with first-episode schizophrenia-spectrum disorder: A longitudinal perspective. *Psychiatry Research, 216*(2), 177-184. doi:10.1016/j.psychres.2013.11.028

Chen, E. Y. H., Kwok, C. L., Chen, R. Y. L., & Kwong, P. P. K. (2001). Insight changes in acute psychotic episodes: A prospective study of Hong Kong Chinese patients. *Journal of Nervous and Mental Disease, 189*(1), 24-30. Retrieved from https://openathens.ovid.com/secure-ssl/home.oa?idpselect=https://kclidp.kcl.ac.uk/idp/shibboleth&entityID=https://kclidp.kcl.ac.uk/idp/shibboleth&?T=JS&CSC=Y&NEWS=N&PAGE=fulltext&D=emed7&AN=32095341

http://sfx.kcl.ac.uk/kings?sid=OVID:embase&id=pmid:&id=doi:10.1097%2F00005053-200101000-00005&genre=article&atitle=Insight+changes+in+acute+psychotic+episodes%3A+A+prospective+study+of+Hong+Kong+Chinese+patients&title=Journal+of+Nervous+and+Mental+Disease&issn=0022-3018&date=2001&volume=189&issue=1&spage=24&aulast=Chen+E.Y.H.&isbn=&__char_set=utf8

Chen, Y. L., Yang, C. Y., Chen, S. J., Chen, Y. C., & Su, C. Y. (2018). Everyday memory problems in alcohol abuse and dependence: Frequency, patterns and patient-proxy agreement. *Psychiatry Res, 261*, 488-497. doi:10.1016/j.psychres.2018.01.016

Collins, A. A., Remington, G. J., Coulter, K., & Birkett, K. (1997). Insight, neurocognitive function and symptom clusters in chronic schizophrenia. *Schizophr Res, 27*(1), 37-44. doi:10.1016/S0920-9964(97)00075-3

Comacchio, C., Lasalvia, A., Bonetto, C., Cristofalo, D., Miglietta, E., Petterlini, S., . . . Group, P. V. (2020). Gender and 5-years course of psychosis patients: focus on clinical and social variables. *Arch Womens Ment Health, 23*(1), 63-70. doi:10.1007/s00737-019-0945-3

Cuesta, M. J., & Peralta, V. (1994). Lack of insight in schizophrenia. *Schizophr Bull, 20*(2), 359-366.

David, A., Buchanan, A., Reed, A., & Almeida, O. (1992). The assessment of insight in psychosis. *Br J Psychiatry, 161*(NOV.), 599-602. doi:10.1192/bjp.161.5.599

David, A., van Os, J., Jones, P., Harvey, I., Foerster, A., & Fahy, T. (1995). Insight and psychotic illness. Cross-sectional and longitudinal associations. *Br J Psychiatry, 167*(5), 621-628. doi:10.1192/bjp.167.5.621

de Assis da Silva, R., Mograbi, D. C., Silveira, L. A., Nunes, A. L., Novis, F. D., Landeira-Fernandez, J., & Cheniaux, E. (2015). Insight Across the Different Mood States of Bipolar Disorder. *Psychiatr Q, 86*(3), 395-405. doi:10.1007/s11126-015-9340-z

de Castro Zilli, B. B. C., & Damasceno, B. P. (2007). Anosognosia in Alzheimer's disease: A neuropsychological approach. *Dementia & Neuropsychologia, 1*(1), 81-88. Retrieved from https://openathens.ovid.com/secure-ssl/home.oa?idpselect=https://kclidp.kcl.ac.uk/idp/shibboleth&entityID=https://kclidp.kcl.ac.uk/idp/shibboleth&?T=JS&CSC=Y&NEWS=N&PAGE=fulltext&D=psyc5&AN=2013-38053-013

http://sfx.kcl.ac.uk/kings?sid=OVID:psycdb&id=pmid:&id=doi:10.1590%2FS1980-57642008DN10100013&genre=article&atitle=Anosognosia+in+Alzheimer%27s+disease%3A+A+neuropsychological+approach.&title=Dementia+%26+Neuropsychologia&issn=1980-5764&date=2007&volume=1&issue=1&spage=81&aulast=de+Castro+Zilli%2C+Barbara+Bomfim+Caiado&isbn=&__char_set=utf8

Duarte Gigante, A., & Castel, S. (2004). Insight into schizophrenia: A comparative study between pateints and family members. *Sao Paulo Medical Journal, 122*(6), 246-251. Retrieved from https://openathens.ovid.com/secure-ssl/home.oa?idpselect=https://kclidp.kcl.ac.uk/idp/shibboleth&entityID=https://kclidp.kcl.ac.uk/idp/shibboleth&?T=JS&CSC=Y&NEWS=N&PAGE=fulltext&D=emed8&AN=40185906

http://sfx.kcl.ac.uk/kings?sid=OVID:embase&id=pmid:&id=doi:&genre=article&atitle=Insight+into+schizophrenia%3A+A+comparative+study+between+pateints+and+family+members&title=Sao+Paulo+Medical+Journal&issn=1516-

3180&date=2004&volume=122&issue=6&spage=246&aulast=Duarte+Gigante+A.&isbn=&__char_set=utf8

Farias, S. T., Mungas, D., & Jagust, W. (2005). Degree of discrepancy between self and other-reported everyday functioning by cognitive status: dementia, mild cognitive impairment, and healthy elders. *Int J Geriatr Psychiatry, 20*(9), 827-834. doi:10.1002/gps.1367

Gilleen, J., Greenwood, K., & David, A. S. (2011). Domains of awareness of schizophrenia. *Schizophrenia Bulletin, 37*(1), 61-72. doi:schbul/sbq100

Goldberg, R. W., Green-Paden, L. D., Lehman, A. F., & Gold, J. M. (2001). Correlates of insight in serious mental illness. *J Nerv Ment Dis, 189*(3), 137-145.

Gomez-de-Regil, L. (2015). Insight and illness perception in Mexican patients with psychosis. *Schizophrenia Research: Cognition, 2*(1), 33-38. Retrieved from https://openathens.ovid.com/secure-ssl/home.oa?idpselect=https://kclidp.kcl.ac.uk/idp/shibboleth&entityID=https://kclidp.kcl.ac.uk/idp/shibboleth&?T=JS&CSC=Y&NEWS=N&PAGE=fulltext&D=emed16&AN=602811173

http://sfx.kcl.ac.uk/kings?sid=OVID:embase&id=pmid:&id=doi:10.1016%2Fj.scog.2015.01.002&genre=article&atitle=Insight+and+illness+perception+in+Mexican+patients+with+psychosis&title=Schizophrenia+Research%3A+Cognition&issn=2215-0013&date=2015&volume=2&issue=1&spage=33&aulast=Gomez-de-Regil+L.&isbn=&__char_set=utf8

Greenfeld, D. G., Anyan, W. R., Hobart, M., Quinlan, D. M., & Plantes, M. (1991). Insight into illness and outcome in anorexia nervosa. *International Journal of Eating Disorders, 10*(1), 101-109. Retrieved from https://openathens.ovid.com/secure-ssl/home.oa?idpselect=https://kclidp.kcl.ac.uk/idp/shibboleth&entityID=https://kclidp.kcl.ac.uk/idp/shibboleth&?T=JS&CSC=Y&NEWS=N&PAGE=fulltext&D=emed4&AN=21035272

http://sfx.kcl.ac.uk/kings?sid=OVID:embase&id=pmid:&id=doi:&genre=article&atitle=Insight+into+illness+and+outcome+in+anorexia+nervosa&title=International+Journal+of+Eating+Disorders&issn=0276-3478&date=1991&volume=10&issue=1&spage=101&aulast=Greenfeld+D.G.&isbn=&__char_set=utf8

https://onlinelibrary.wiley.com/doi/pdf/10.1002/1098-108X%28199101%2910%3A1%3C101%3A%3AAID-EAT2260100111%3E3.0.CO%3B2-7

Hanseeuw, B. J., Scott, M. R., Sikkes, S. A. M., Properzi, M., Gatchel, J. R., Salmon, E., . . . Alzheimer's Disease Neuroimaging, I. (2020). Evolution of anosognosia in alzheimer's disease and its relationship to amyloid. *Ann Neurol, 87*(2), 267-280. doi:10.1002/ana.25649

Hoth, K. F., Paulsen, J. S., Moser, D. J., Tranel, D., Clark, L. A., & Bechara, A. (2007). Patients with Huntington's disease have impaired awareness of cognitive, emotional, and functional abilities. *J Clin Exp Neuropsychol, 29*(4), 365-376. doi:10.1080/13803390600718958

Jolfaei, A. G., & Shabani, A. (2012). Mood disorders insight scale: Validation of Persian version. *Journal of Research in Medical Sciences, 17*(2), 186-189. Retrieved from https://openathens.ovid.com/secure-ssl/home.oa?idpselect=https://kclidp.kcl.ac.uk/idp/shibboleth&entityID=https://kclidp.kcl.ac.uk/idp/shibboleth&?T=JS&CSC=Y&NEWS=N&PAGE=fulltext&D=emed13&AN=365720052

http://sfx.kcl.ac.uk/kings?sid=OVID:embase&id=pmid:&id=doi:&genre=article&atitle=Mood+disorders+insight+scale%3A+Validation+of+Persian+version&title=Journal+of+Research+in+Medical+Sciences&issn=1735-1995&date=2012&volume=17&issue=2&spage=186&aulast=Vazmalaei+H.A.&isbn=&__char_set=utf8

Jong, S. K., Byoung, K. P., Gap, J. K., Sung, S. K., Jin, G. J., Mi, K. O., & Jang, K. O. (2007). The role of alcoholics' insight in abstinence from alcohol in male Korean alcohol dependents. *Journal of Korean Medical Science, 22*(1), 132-137. Retrieved from https://openathens.ovid.com/secure-ssl/home.oa?idpselect=https://kclidp.kcl.ac.uk/idp/shibboleth&entityID=https://kclidp.kcl.ac.uk/idp/shibboleth&?T=JS&CSC=Y&NEWS=N&PAGE=fulltext&D=emed10&AN=46293615

http://sfx.kcl.ac.uk/kings?sid=OVID:embase&id=pmid:&id=doi:&genre=article&atitle=The+role+of+alcoholics%27+insight+in+abstinence+from+alcohol+in+male+Korean+alcohol+dependents&title=Journal+of+Korean+Medical+Science&issn=1011-8934&date=2007&volume=22&issue=1&spage=132&aulast=Jong+S.K.&isbn=&__char_set=utf8

https://synapse.koreamed.org/Synapse/Data/PDFData/0063JKMS/jkms-22-132.pdf

Kemp, R. A., & Lambert, T. J. (1995). Insight in schizophrenia and its relationship to psychopathology. *Schizophr Res, 18*(1), 21-28. doi:10.1016/0920-9964(95)00018-6

Keshavan, M. S., Rabinowitz, J., DeSmedt, G., Harvey, P. D., & Schooler, N. (2004). Correlates of insight in first episode psychosis. *Schizophr Res, 70*(2-3), 187-194. doi:10.1016/j.schres.2003.11.007

Kim, J., Ozzoude, M., Nakajima, S., Shah, P., Caravaggio, F., Iwata, Y., . . . Gerretsen, P. (2020). Insight and medication adherence in schizophrenia: An analysis of the CATIE trial. *Neuropharmacology, 168*, 107634. doi:10.1016/j.neuropharm.2019.05.011

Klaas, H. S., Clemence, A., Marion-Veyron, R., Antonietti, J. P., Alameda, L., Golay, P., & Conus, P. (2017). Insight as a social identity process in the evolution of psychosocial functioning in the early phase of psychosis. *Psychol Med, 47*(4), 718-729. doi:10.1017/S0033291716002506

Kumar, A., Kumar, S., Khan, N. M., & Mishra, S. (2013). Course of insight in manic episode. *J Postgrad Med, 59*(3), 186-189. doi:10.4103/0022-3859.118035

Liu, J. L., Abdin, E., Vaingankar, J. A., Shafie, S. B., Jeyagurunathan, A., Shahwan, S., . . . Subramaniam, M. (2017). The relationship among unawareness of memory impairment, depression, and dementia in older adults with memory impairment in Singapore. *Psychogeriatrics, 17*(6), 430-438. doi:10.1111/psyg.12270

Lysaker, P., & Bell, M. (1995). Work rehabilitation and improvements in insight in schizophrenia. *J Nerv Ment Dis, 183*(2), 103-106.

Lysaker, P. H., Dimaggio, G., Buck, K. D., Callaway, S. S., Salvatore, G., Carcione, A., . . . Stanghellini, G. (2011). Poor insight in schizophrenia: links between different forms of metacognition with awareness of symptoms, treatment need, and consequences of illness. *Compr Psychiatry, 52*(3), 253-260. doi:10.1016/j.comppsych.2010.07.007

Lysaker, P. H., Gagen, E., Wright, A., Vohs, J. L., Kukla, M., Yanos, P. T., & Hasson-Ohayon, I. (2019). Metacognitive Deficits Predict Impaired Insight in Schizophrenia Across Symptom Profiles: A Latent Class Analysis. *Schizophrenia Bulletin, 45*(1), 48-56. doi:10.1093/schbul/sby142

Lysaker, P. H., Whitney, K. A., & Davis, L. W. (2006). Awareness of illness in schizophrenia: associations with multiple assessments of executive function. *J Neuropsychiatry Clin Neurosci, 18*(4), 516-520. doi:10.1176/jnp.2006.18.4.516

Marazziti, D., Dell'Osso, L., Di Nasso, E., Pfanner, C., Presta, S., Mungai, F., & Cassano, G. B. (2002). Insight in obsessive-compulsive disorder: a study of an Italian sample. *Eur Psychiatry, 17*(7), 407-410. Retrieved from https://ac.els-cdn.com/S0924933802006971/1-s2.0-S0924933802006971-main.pdf?_tid=59dc86a0-a974-4120-a89d-2236173e2f7b&acdnat=1547815517_770a0c79f996def979af6fa811735225

Maremmani, A. G., Rovai, L., Rugani, F., Pacini, M., Lamanna, F., Bacciardi, S., . . . Maremmani, I. (2012). Correlations between awareness of illness (insight) and history of addiction in heroin-addicted patients. *Front Psychiatry, 3*(JUL), 61. doi:10.3389/fpsyt.2012.00061

Martyr, A., Clare, L., Nelis, S. M., Markova, I. S., Roth, I., Woods, R. T., . . . Morris, R. G. (2012). Verbal fluency and awareness of functional deficits in early-stage dementia. *Clin Neuropsychol, 26*(3), 501-519. doi:10.1080/13854046.2012.665482

McCabe, R., Quayle, E., Beirne, A. D., & Duane, M. M. A. (2002). Insight, global neuropsychological functioning, and symptomatology in chronic schizophrenia. *Journal of Nervous & Mental Disease, 190*(8), 519-525. Retrieved from http://search.ebscohost.com/login.aspx?direct=true&db=cin20&AN=106982530&site=ehost-live

Mintz, E., Wise, T. N., & Helmkamp, C. (2004). Insight and alexithymia in hospitalized psychiatric patients. *Isr J Psychiatry Relat Sci, 41*(2), 111-117. Retrieved from https://www.ncbi.nlm.nih.gov/pubmed/15478456

Mograbi, D. C., Ferri, C. P., Sosa, A. L., Stewart, R., Laks, J., Brown, R., & Morris, R. G. (2012). Unawareness of memory impairment in dementia: a population-based study. *Int Psychogeriatr, 24*(6), 931-939. doi:10.1017/S1041610211002730

Moore, O., Cassidy, E., Carr, A., & O'Callaghan, E. (1999). Unawareness of illness and its relationship with depression and self-deception in schizophrenia. *Eur Psychiatry, 14*(5), 264-269. Retrieved from https://ac.els-cdn.com/S0924933899001728/1-s2.0-S0924933899001728-main.pdf?_tid=35b1905d-47e0-4a00-bb9f-e6d5e14eb879&acdnat=1548167488_b6caecd63887dc5a3a258a1d3a7b672a

Mullick, M., Miller, L. J., & Jacobsen, T. (2001). Insight into mental illness and child maltreatment risk among mothers with major psychiatric disorders. *Psychiatr Serv, 52*(4), 488-492. doi:10.1176/appi.ps.52.4.488

Parellada, M., Fraguas, D., Bombín, I., Otero, S., Castro-Fornieles, J., Baeza, I., . . . Arango, C. (2009). Insight correlates in child- and adolescent-onset first episodes of psychosis: results from the CAFEPS study. *Psychological Medicine, 39*(9), 1433-1445. doi:10.1017/S0033291708004868

Pia, L., Spinazzola, L., Garbarini, F., Bellan, G., Piedimonte, A., Fossataro, C., . . . Berti, A. (2014). Anosognosia for hemianaesthesia: a voxel-based lesion-symptom mapping study. *Cortex, 61*, 158-166. doi:10.1016/j.cortex.2014.08.006

Pijnenborg, G. H., Timmerman, M. E., Derks, E. M., Fleischhacker, W. W., Kahn, R. S., & Aleman, A. (2015). Differential effects of antipsychotic drugs on insight in first episode schizophrenia: data from the European First-Episode Schizophrenia Trial (EUFEST). *European neuropsychopharmacology, 25*(6), 808-816. doi:10.1016/j.euroneuro.2015.02.012

Pillai, J. A., Bonner-Jackson, A., Floden, D., Fernandez, H., & Leverenz, J. B. (2018). Lack of Accurate Self-appraisal is Equally Likely in MCI from Parkinson's Disease and Alzheimer's Disease. *Mov Disord Clin Pract, 5*(3), 283-289. doi:10.1002/mdc3.12606

Reed, B. R., Jagust, W. J., & Coulter, L. (1993). Anosognosia in Alzheimer's disease: relationships to depression, cognitive function, and cerebral perfusion. *J Clin Exp Neuropsychol, 15*(2), 231-244. doi:10.1080/01688639308402560

Sanchez-Torres, A. M., Zarzuela, A., Peralta, V., & Cuesta, M. J. (2015). The association of lifetime insight and cognition in psychosis. *Schizophr Res, 162*(1-3), 183-188. doi:10.1016/j.schres.2014.12.019

Sanz, M., Constable, G., Lopez-Ibor, I., Kemp, R., & David, A. S. (1998). A comparative study of insight scales and their relationship to psychopathological and clinical variables. *Psychol Med, 28*(2), 437-446.

Saravanan, B., Jacob, K. S., Johnson, S., Prince, M., Bhugra, D., & David, A. S. (2007). Belief models in first episode schizophrenia in South India. *Soc Psychiatry Psychiatr Epidemiol, 42*(6), 446-451. doi:10.1007/s00127-007-0186-z

Sasse, N., Gibbons, H., Wilson, L., Martinez-Olivera, R., Schmidt, H., Hasselhorn, M., . . . von Steinbuchel, N. (2013). Self-awareness and health-related quality of life after traumatic brain injury. *J Head Trauma Rehabil, 28*(6), 464-472. doi:10.1097/HTR.0b013e318263977d

Schwartz, R. C., Cohen, B. N., & Grubaugh, A. (1997). Does insight affect long-term impatient treatment outcome in chronic schizophrenia? *Compr Psychiatry, 38*(5), 283-288. Retrieved from https://ac.els-cdn.com/S0010440X97900614/1-s2.0-S0010440X97900614-main.pdf?_tid=87c9a341-5c7d-41a7-8289-0e9a539f0350&acdnat=1548167406_c7cda7a244998512df4689298094e1d9

Setkowski, K., van der Post, L. F. M., Peen, J., & Dekker, J. J. M. (2016). Changing patient perspectives after compulsory admission and the risk of re-admission during 5 years of follow-up: The Amsterdam Study of Acute Psychiatry IX. *International Journal of Social Psychiatry, 62*(6), 578-588. doi:10.1177/0020764016655182

Sherer, M., Hart, T., Nick, T. G., Whyte, J., Thompson, R. N., & Yablon, S. A. (2003). Early impaired self-awareness after traumatic brain injury. *Arch Phys Med Rehabil, 84*(2), 168-176. doi:10.1053/apmr.2003.50045

Sherer, M., Hart, T., Whyte, J., Nick, T. G., & Yablon, S. A. (2005). Neuroanatomic basis of impaired self-awareness after traumatic brain injury - Findings from early computed tomography. *Journal of Head Trauma Rehabilitation, 20*(4), 287-300. doi:10.1097/00001199-200507000-00002

Smith, C. M., Barzman, D., & Pristach, C. A. (1997). Effect of patient and family insight on compliance of schizophrenic patients. *J Clin Pharmacol, 37*(2), 147-154. doi:10.1002/j.1552-4604.1997.tb04773.x

Smith, L. T., Shelton, C. L., Berk, M., Hasty, M. K., Cotton, S. M., Henry, L., . . . Conus, P. (2014). The impact of insight in a first-episode mania with psychosis population on outcome at 18 months. *J Affect Disord, 167*, 74-79. doi:10.1016/j.jad.2014.05.055

Tariku, M., Demilew, D., Fanta, T., Mekonnen, M., & Abebaw Angaw, D. (2019). Insight and Associated Factors among Patients with Schizophrenia in Mental Specialized Hospital, Ethiopia, 2018. *Psychiatry J, 2019*, 2453862. doi:10.1155/2019/2453862

Turró-Garriga, O., Garre-Olmo, J., López-Pousa, S., Vilalta-Franch, J., Reñé-Ramírez, R., & Conde-Sala, J. L. (2014). Abridged scale for the screening anosognosia in patients with dementia. *Journal of Geriatric Psychiatry & Neurology, 27*(3), 220-226. doi:10.1177/0891988714527515

van Vliet, D., de Vugt, M. E., Kohler, S., Aalten, P., Bakker, C., Pijnenburg, Y. A., . . . Verhey, F. R. (2013). Awareness and its association with affective symptoms in young-onset and late-onset Alzheimer disease: a prospective study. *Alzheimer Dis Assoc Disord, 27*(3), 265-271. doi:10.1097/WAD.0b013e31826cffa5

Vanderploeg, R. D., Belanger, H. G., Duchnick, J. D., & Curtiss, G. (2007). Awareness problems following moderate to severe traumatic brain injury: prevalence, assessment methods, and injury correlates. *Journal of Rehabilitation Research & Development, 44*(7), 937-949. Retrieved from http://search.ebscohost.com/login.aspx?direct=true&db=cin20&AN=105769329&site=ehost-live

Vannini, P., Arnariglio, R., Hanseeuw, B., Johnson, K. A., McLaren, D. G., Chhatwal, J., . . . Sperling, R. A. (2017). Memory self-awareness in the preclinical and prodromal stages of Alzheimer's disease. *Neuropsychologia, 99*, 343-349. doi:10.1016/j.neuropsychologia.2017.04.002

Vasterling, J. J., Seltzer, B., & Watrous, W. E. (1997). Longitudinal assessment of deficit unawareness in Alzheimer's disease. *Neuropsychiatry Neuropsychology and Behavioral Neurology, 10*(3), 197-202.

Vigne, P., de Menezes, G. B., Harrison, B. J., & Fontenelle, L. E. (2014). A study of poor insight in social anxiety disorder. *Psychiatry Research, 219*(3), 556-561. doi:10.1016/j.psychres.2014.05.033

Welten, C. C., Koeter, M. W., Wohlfarth, T. D., Storosum, J. G., van den Brink, W., Gispen-de Wied, C. C., . . . Denys, D. A. (2016). Does Insight Affect the Efficacy of Antipsychotics in Acute Mania?: An Individual Patient Data Regression Meta-Analysis. *J Clin Psychopharmacol, 36*(1), 71-76. doi:10.1097/jcp.0000000000000435

Wiffen, B. D. R., Rabinowitz, J., Lex, A., & David, A. S. (2010). Correlates, change and 'state or trait' properties of insight in schizophrenia. *Schizophrenia research, 122*(1-3), 94-103. doi:10.1016/j.schres.2010.03.005

Wilson, R. S., Boyle, P. A., Yu, L., Barnes, L. L., Sytsma, J., Buchman, A. S., . . . Schneider, J. A. (2015). Temporal course and pathologic basis of unawareness of memory loss in dementia. *Neurology, 85*(11), 984-991. doi:10.1212/WNL.0000000000001935

Yen, C.-F., Chen, C.-S., Yeh, M.-L., Yen, J.-Y., Ker, J.-H., & Yang, S.-J. (2002). Comparison of insight in patients with schizophrenia and bipolar disorder in remission. *Journal of Nervous and Mental Disease, 190*(12), 847-849. Retrieved from https://openathens.ovid.com/secure-ssl/home.oa?idpselect=https://kclidp.kcl.ac.uk/idp/shibboleth&entityID=https://kclidp.kcl.ac.uk/idp/shibboleth&?T=JS&CSC=Y&NEWS=N&PAGE=fulltext&D=psyc4&AN=2002-11671-010

http://sfx.kcl.ac.uk/kings?sid=OVID:psycdb&id=pmid:&id=doi:10.1097%2F00005053-200212000-00008&genre=article&atitle=Comparison+of+insight+in+patients+with+schizophrenia+and+bipolar+disorder+in+remission.&title=Journal+of+Nervous+and+Mental+Disease&issn=0022-3018&date=2002&volume=190&issue=12&spage=847&aulast=Yen%2C+Cheng-Fang&isbn=&__char_set=utf8

Yen, C.-F., Hsiao, R. C., Ries, R., Liu, S.-C., Huang, C.-F., Chang, Y.-P., & Yu, M.-L. (2008). Insight into alcohol-related problems and its associations with severity of alcohol consumption, mental health status, race, and level of acculturation in southern Taiwanese indigenous people with alcoholism. *The American Journal of Drug and Alcohol Abuse, 34*(5), 553-561. Retrieved from https://openathens.ovid.com/secure-ssl/home.oa?idpselect=https://kclidp.kcl.ac.uk/idp/shibboleth&entityID=https://kclidp.kcl.ac.uk/idp/shibboleth&?T=JS&CSC=Y&NEWS=N&PAGE=fulltext&D=psyc6&AN=2008-14366-005

http://sfx.kcl.ac.uk/kings?sid=OVID:psycdb&id=pmid:&id=doi:10.1080%2F00952990802295220&genre=article&atitle=Insight+into+alcohol-related+problems+and+its+associations+with+severity+of+alcohol+consumption%2C+mental+health+status%2C+race%2C+and+level+of+acculturation+in+southern+Taiwanese+indigenous+people+with+alcoholism.&title=The+American+Journal+of+Drug+and+Alcohol+Abuse&issn=0095-2990&date=2008&volume=34&issue=5&spage=553&aulast=Yen%2C+Cheng-Fang&isbn=&__char_set=utf8

https://www.tandfonline.com/doi/pdf/10.1080/00952990802295220?needAccess=true

Yen, C. F., Chen, C. S., Yeh, M. L., Yang, S. J., Ke, J. H., & Yen, J. Y. (2003). Changes of insight in manic episodes and influencing factors. *Compr Psychiatry, 44*(5), 404-408. doi:10.1016/S0010-440X(03)00107-X

Young, D. A., Davila, R., & Scher, H. (1993). Unawareness of illness and neuropsychological performance in chronic schizophrenia. *Schizophr Res, 10*(2), 117-124. doi:10.1016/0920-9964(93)90046-l

Zimmermann, N., Mograbi, D. C., Hermes-Pereira, A., Fonseca, R., & Prigatano, G. P. (2017). Memory and executive functions correlates of self-awareness in traumatic brain injury. *Cognitive Neuropsychiatry, 22*(4), 346-360. doi:10.1080/13546805.2017.1330191

Other References

Borenstein, M., Hedges, L. V., Higgins, J. P., & Rothstein, H. R. (2009). Converting among effect sizes. *Introduction to meta-analysis*, 45-49.

(Amanzio et al., 2011; Ampalam, Deepthi, & Vadaparty, 2012; Ayesa-Arriola et al., 2014; Baier, Karnath, Baier, & Karnath, 2005; Bassitt, Neto, de Castro, & Busatto, 2007; Beland & Lepage, 2017; Bellino, Patria, Ziero, & Bogetto, 2005; Bivona et al., 2008; Błądziński et al., 2019; Bota, Munro, Ricci, & Bota, 2006; Castrillo Sanz et al., 2016; Cernovsky, Landmark, Merskey, & Husni, 2004; Chapman et al., 2019; Chapman et al., 2018; Chapman et al., 2020; Cherian et al., 2012; Chesnel et al., 2018; Cines et al., 2015; Ciurli et al., 2010; Clare, Whitaker, & Nelis, 2010; Cobo et al., 2020; Conde-Sala et al., 2013; Contador, Mograbi, Fernandez-Calvo, Benito-Leon, & Bermejo-Pareja, 2020; Cosentino et al., 2007; Cosentino, Metcalfe, Cary, De Leon, & Karlawish, 2011; Cuffel, Alford, Fischer, & Owen, 1996; Danki, Dilbaz, Okay, & Telci, 2007; De Berardis et al., 2005; De Berardis et al., 2008; De Carolis et al., 2015; DeFeis et al., 2019; Depp et al., 2014; Derouesne et al., 1999; V. Dias, Brissos, & Carita, 2008; V. V. Dias, Brissos, Frey, & Kapczinski, 2008; Diez-Martin et al., 2014; Donohoe, Donnell, Owens, & O'Callaghan, 2004; M. Dourado, Marinho, Soares, Engelhardt, & Laks, 2007; M. C. N. Dourado, Laks, & Mograbi, 2019; Elvish, Simpson, & Ball, 2010; Emami, Guimond, Chakravarty, & Lepage, 2016; Faget-Agius et al., 2012; Feher, Mahurin, Inbody, Crook, & Pirozzolo, 1991; Fennig et al., 1996; Fu et al., 2017; Fujimoto et al., 2017; Gambina et al., 2014; Garg, Cheema, & Raj, 2018; Philip Gerretsen et al., 2013; P. Gerretsen et al., 2017; Ghaemi, Stoll, & Pope, 1995; Gilleen, Greenwood, Archer, Lovestone, & David, 2012; Hamann, Kruse, Schmitz, Kissling, & Pajonk, 2010; Hannesdottir, Morris, Hannesdottir, & Morris, 2007; Hanyu et al., 2008; Heinrichs, Cohen, & Carpenter, 1985; Himle, Van Etten, Janeck, & Fischer, 2006; Jacob, Larson, & Storch, 2014; Jeong et al., 2017; Kalbe et al., 2005; Kao & Liu, 2010; Karadag et al., 2011; Karow et al., 2008; Kashyap, Kumar, Kandavel, & Reddy, 2012; Kazui et al., 2006; Kelly et al., 2004; Kishore, Samar, Reddy, Chandrasekhar, & Thennarasu, 2004; Kleim et al., 2009; Konstantakopoulos et al., 2013; Kortte et al., 2015; Lacerda, Santos, Belfort, Neto, & Dourado, 2018; Lacerda, Santos, Neto, & Dourado, 2017; Lamar, Lasarev, & Libon, 2002; Loebel, Dager, Berg, & Hyde, 1990; Lopez, Becker, Somsak, Dew, & Dekosky, 1994; P. H. Lysaker, Bell, Bryson, & Kaplan, 1998; Macpherson, Jerrom, & Hughes, 1996; Maeshima et al., 1997; Martyr, Nelis, & Clare, 2014; Mayelle, El Haj, & Antoine, 2019; McEvoy et al., 2006; Michel et al., 2013; Mohamed et al., 2009; Molina-Andreu et al., 2014; Moro et al., 2016; Noe et al., 2005; Onen, Karakas Ugurlu, & Caykoylu, 2013; Orfei et al., 2010; Othman & Huri, 2017; Ozkiris, Essizoglu, Gulec, & Aksaray, 2015; Ozzoude et al., 2019; Prus, Wiedl, & Waldorf, 2012; Rathod, Kingdon, Smith, & Turkington, 2005; Rossell, Coakes, Shapleske, Woodruff, & David, 2003; Schennach et al., 2012; Sedaghat et al., 2010; Senturk et al., 2017; Shad, Muddasani, Prasad, Sweeney, & Keshavan, 2004; Shimshoni, Reuven, Dar, & Hermesh, 2011; Silva et al., 2016; Sitman, Sela, Erez, Bloch, & Levkovitz, 2012; Sousa et al., 2015; Spalletta, Girardi, Caltagirone, & Orfei, 2012; Starkstein, Brockman, Bruce, & Petracca, 2010; Starkstein et al., 2006; Starkstein, Sabe, Cuerva, Kuzis, & Leiguarda, 1997; Szepietowska & Kuzaka, 2019; Therriault et al., 2018; Tolin, Fitch, Frost, & Steketee, 2008; Tordesillas-Gutierrez et al., 2018; Tremont & Alosco, 2011; Tumkaya et al., 2019; Turksoy, Tukel, Ozdemir, & Karali, 2002; Turró-Garriga et al., 2016; Turro-Garriga et al., 2013; Valiente, Provencio, Espinosa, Chaves, & Fuentenebro, 2011; J. J. Vasterling, Seltzer, Carpenter, & Thompson, 1997; Jennifer J. Vasterling, Seltzer, Foss, & Vanderbrook, 1995; Verhey, Rozendaal, Ponds, & Jolles, 1993; Verhulsdonk, Quack, HÃ¶ft, Lange-Asschenfeldt, & Supprian, 2013; Visser et al., 2017; Wang et al., 2011; Weiler, Fleisher, & McArthur-Campbell, 2000; Wibawa et al., 2020; Woon, Khoo, Baharudin, & Midin, 2020; Xiang et al., 2012; Yeh et al., 2014; C. F. Yen et al., 2008; Yoon et al., 2017; Zhang et al., 2016)

(Aalten et al., 2006; Almeida, Levy, Howard, & David, 1996; Amador et al., 1994; Arbel, Koren, Klein, & Latzer, 2013; Berg et al., 2018; Bianchini et al., 2014; Buchy, Bodnar, Malla, Joober, & Lepage, 2010; Burton, Harvey, Patterson, & Twamley, 2016; K. K. Chan, 2016; S. K. W. Chan et al., 2014; E. Y. H. Chen, Kwok, Chen, & Kwong, 2001; Y. L. Chen, Yang, Chen, Chen, & Su, 2018; Collins, Remington, Coulter, & Birkett, 1997; Comacchio et al., 2020; Cuesta & Peralta, 1994; David, Buchanan, Reed, & Almeida, 1992; David et al., 1995; de Assis da Silva et al., 2015; de Castro Zilli & Damasceno, 2007; Duarte Gigante & Castel, 2004; Farias, Mungas, & Jagust, 2005; Gilleen, Greenwood, & David, 2011; Goldberg, Green-Paden, Lehman, & Gold, 2001; Gomez-de-Regil, 2015; Greenfeld, Anyan, Hobart, Quinlan, & Plantes, 1991; Hanseeuw et al., 2020; Hoth et al., 2007; Jolfaei & Shabani, 2012; Jong et al., 2007; Kemp & Lambert, 1995; Keshavan, Rabinowitz, DeSmedt, Harvey, & Schooler, 2004; Kim et al., 2020; Klaas et al., 2017; Kumar, Kumar, Khan, & Mishra, 2013; Liu et al., 2017; P. Lysaker & Bell, 1995; P. H. Lysaker et al., 2011; P. H. Lysaker et al., 2019; P. H. Lysaker, Whitney, & Davis, 2006; Marazziti et al., 2002; Maremmani et al., 2012; Martyr et al., 2012; McCabe, Quayle, Beirne, & Duane, 2002; Mintz, Wise, & Helmkamp, 2004; Mograbi et al., 2012; Moore, Cassidy, Carr, & O'Callaghan, 1999; Mullick, Miller, & Jacobsen, 2001; Parellada et al., 2009; Pia et al., 2014; Pijnenborg et al., 2015; Pillai, Bonner-Jackson, Floden, Fernandez, & Leverenz, 2018; Reed, Jagust, & Coulter, 1993; Sanchez-Torres, Zarzuela, Peralta, & Cuesta, 2015; Sanz, Constable, Lopez-Ibor, Kemp, & David, 1998; Saravanan et al., 2007; Sasse et al., 2013; Schwartz, Cohen,

& Grubaugh, 1997; Setkowski, van der Post, Peen, & Dekker, 2016; Sherer et al., 2003; Sherer, Hart, Whyte, Nick, & Yablon, 2005; C. M. Smith, Barzman, & Pristach, 1997; L. T. Smith et al., 2014; Tariku, Demilew, Fanta, Mekonnen, & Abebaw Angaw, 2019; Turró-Garriga et al., 2014; van Vliet et al., 2013; Vanderploeg, Belanger, Duchnick, & Curtiss, 2007; Vannini et al., 2017; J. J. Vasterling, Seltzer, & Watrous, 1997; Vigne, de Menezes, Harrison, & Fontenelle, 2014; Welten et al., 2016; Wiffen, Rabinowitz, Lex, & David, 2010; Wilson et al., 2015; C.-F. Yen et al., 2002; C.-F. Yen et al., 2008; C. F. Yen et al., 2003; Young, Davila, & Scher, 1993; Zimmermann, Mograbi, Hermes-Pereira, Fonseca, & Prigatano, 2017)
(Borenstein et al., 2009)
